# *Nezara viridula* (Hemiptera: Pentatomidae) transcriptomic analysis and neuropeptidomics

**DOI:** 10.1038/s41598-018-35386-4

**Published:** 2018-11-22

**Authors:** Andrés Lavore, Lucila Perez-Gianmarco, Natalia Esponda-Behrens, Victorio Palacio, Maria Ines Catalano, Rolando Rivera-Pomar, Sheila Ons

**Affiliations:** 1 Centro de Bioinvestigaciones, Universidad Nacional del Noroeste de Buenos Aires, Pergamino, Argentina; 2 Centro Regional de Estudios Genomicos, Facultad de Ciencias Exactas, Universidad Nacional de La Plata, Buenos Aires, Argentina

## Abstract

Stinkbugs (Hemiptera: Pentatomidae) are of major economic importance as pest of crops. Among the species composing the stinkbug complex, *Nezara viridula* is one of the most abundant in Brazil, Argentina and the Southern USA. However, this species has been poorly characterized at the genetic and physiological level. Here we sequenced and analyzed the complete transcriptome of *N. viridula* male and female adults. We identified neuropeptide precursor genes and G-protein coupled receptors for neuropeptides in this transcriptome. Mature neuropeptides were identified in *N. viridula* brain extracts by liquid chromatography-tandem mass spectrometry. We also analyzed the neuropeptide precursor complement in the genome sequence of *Halyomorpha halys*, another pentatomid of economic relevance. We compared the results in both pentatomids with the well-characterized neuropeptide repertoire from the kissing bug *Rhodnius prolixus* (Hemiptera: Reduviidae). We identified both group-specific features (which could be related to the different feeding habits) and similarities that could be characteristic of Heteroptera. This work contributes to a deeper knowledge of the genetic information of these pests, with a focus on neuroendocrine system characterization.

## Introduction

Stinkbugs (Hemiptera: Pentatomidae) are of major economic importance as crop pests in wide regions of the world, being the most important agricultural insect pests. They feed on plants and seeds by means of their piercing-sucking mouthparts causing deformations, seed abortion, decrease in germination and survival, and transmission of plant pathogens^1,2^. The lower quality of seeds caused by stinkbugs is the main reason of yield reduction in crops, and results in economic losses^1^. Among the species of the stinkbug complex, *Nezara viridula* is one of the most abundant in Brazil, Argentina and Southern USA^2^. This species feeds on a variety of plants, the damage caused to soybean (*Glycine max)*, sunflower (*Helianthus annuus*) and corn (*Zea mays*) being of particular importance. Despite the economic relevance of *N. viridula*, this species has been poorly characterized at the genetic level; only a small number of gene sequences were reported^3–5^. The lack of genetic information makes it difficult to use *N. viridula* for many physiological experiments. Besides, *Halyomorpha halys* is an invasive pest that has rapidly spread across North America, causing severe damage to fruit and vegetable crops^1^. *H. halys* genome has been sequenced (GeneBank accession number GCA_000696795.1) and annotated, but a comprehensive analysis of the most relevant gene families has not been reported to date.

Stinkbugs are controlled by means of neurotoxic insecticides^6^. In Argentina, for example, the insecticide market has tripled in twenty years, stinkbugs being one of its main targets^7^. However, neurotoxic chemicals present serious disadvantages, such as high environmental impact and negative effects on human health and on beneficent species (i.e., pollinators and natural enemies of pests). Moreover, the continuous use of insecticides results in a high selection pressure that leads to the emergence of resistant populations. Hemipterans, in particular, are capable of developing extremely high levels of resistance to neurotoxics (see^8–12^). Cases of failures in the control of stinkbugs with insecticides have already been reported^13^. Hence, the identification of targets for alternative insecticides to complement or replace neurotoxics is an important goal in applied entomology. In order to protect food sources, human health and natural environment, an ideal insecticide should be species-specific, biodegradable and less prone to generate resistant populations.

Neuropeptides and their receptors (mainly G-protein coupled receptors; GPCRs), but also tyrosine-kinase and guanylate-ciyclase receptors) regulate critical physiological processes in insects (see^14^). Therefore, a growing interest in the study of neuroendocrine molecules as potential insecticidal targets does exist, given that they would satisfy the characteristics of new alternative compounds^15,16^. However, the use of neuropeptides and their receptors as pesticides remains underexploited.

The identification and characterization of neuroendocrine-related genes in genomes and transcriptomes have been proposed as the first step in the “genome-to-lead” strategy for achieving new insecticidal solutions^17^. This strategy is harnessed by the use of next-generation sequencing techniques in the study of harmful insects. Here we sequenced and analyzed the complete transcriptome of *N. viridula* male and female adults, and identified neuropeptide precursor genes and their receptors. We used a liquid chromatography/tandem mass spectrometry approach in order to confirm the presence of mature neuropeptides encoded in some of these precursors in brain extracts. We also analyzed neuropeptide precursors in *H. halys* genome, and compared the results in both pentatomids with the well-characterized neuropeptide repertoire from the kissing bug *R. prolixus* (Hemiptera: Reduviidae)^14,18–21^. Interestingly, we identified group-specific features (which could be related to the different feeding habits), and similarities that could be characteristic of Heteroptera. This work contributes with genomic information and structural knowledge on the neuroendocrine system, a necessary step for advancing in molecular, biochemical and physiological studies with these species.

## Results and Discussion

### *N. viridula* transcriptome characterization and completeness analysis

A total of 280.4 megabases of raw sequence data was generated, resulting in 299,148 assembled transcripts (Supplementary Information (Supp. Info.) 1). We used different bioinformatics tools to estimate the coverage (see Methods), obtaining values ≥94.4% (Supp. Info. 1). These coverage metrics indicated that the assembled transcriptome is sufficient for a meaningful analysis and the characterization of gene families.

Orthologue gene analysis among four Hemipteran species: *N. viridula*, *H. halys*, *R. prolixus* (Reduviidae) and *Oncopeltus fasciatus* (Lygaeidae) was performed (Supp. Info. 2). In order to conduct a transcriptome-composition representation analysis, we carried out a functional annotation of *N. viridula* transcriptome using BLAST2GO (Supp. Info. Dataset 3). Using a Kyoto Encyclopedia of Genes and Genomes (KEGG) database we did a pathway analysis and could identify 125 total pathways (Supp. Info. Dataset 3). The functional annotation for the 415 transcripts shared only by the phytophagous *N. viridula, O. fasciatus* and *H. halys* resulted in the assignment of 771 GO terms and the annotation of 294 transcripts (Supp. Info. Dataset 4). KEGG pathway analysis allowed for the detection of a short list of 36 enzymatic pathways, including 39 enzymes related to central metabolism and only present in these three phytophagous insects but not in *R. prolixus* (Supp. Info. Dataset 4). The BLAST2GO terms and KEGG pathway representation found in *N. viridula* transcriptome correlate with the ones observed in *H. halys*, in agreement with their close evolutive origin.

### Neuropeptide precursor genes in *N. viridula* and *H. halys*

Despite their economic importance and the potential of the neuroendocrine system as a source of targets for insect pest control^15^, the available information on the Pentatomidae neuroendocrine system is very limited. To our knowledge, the identification of products of only 8 neuropeptide precursors has been reported (pyrokinin (PK), myosuppressin (MS), corazonin (CRZ), adipokinetic hormone (AKH), short neuropeptide F (sNPF), PVK/CAPA peptides, allatotropin (AT) and tachykinins (TK))^5,22–25^. A comprehensive analysis of neuropeptide precursor genes has not been reported yet.

**Figure 1:**
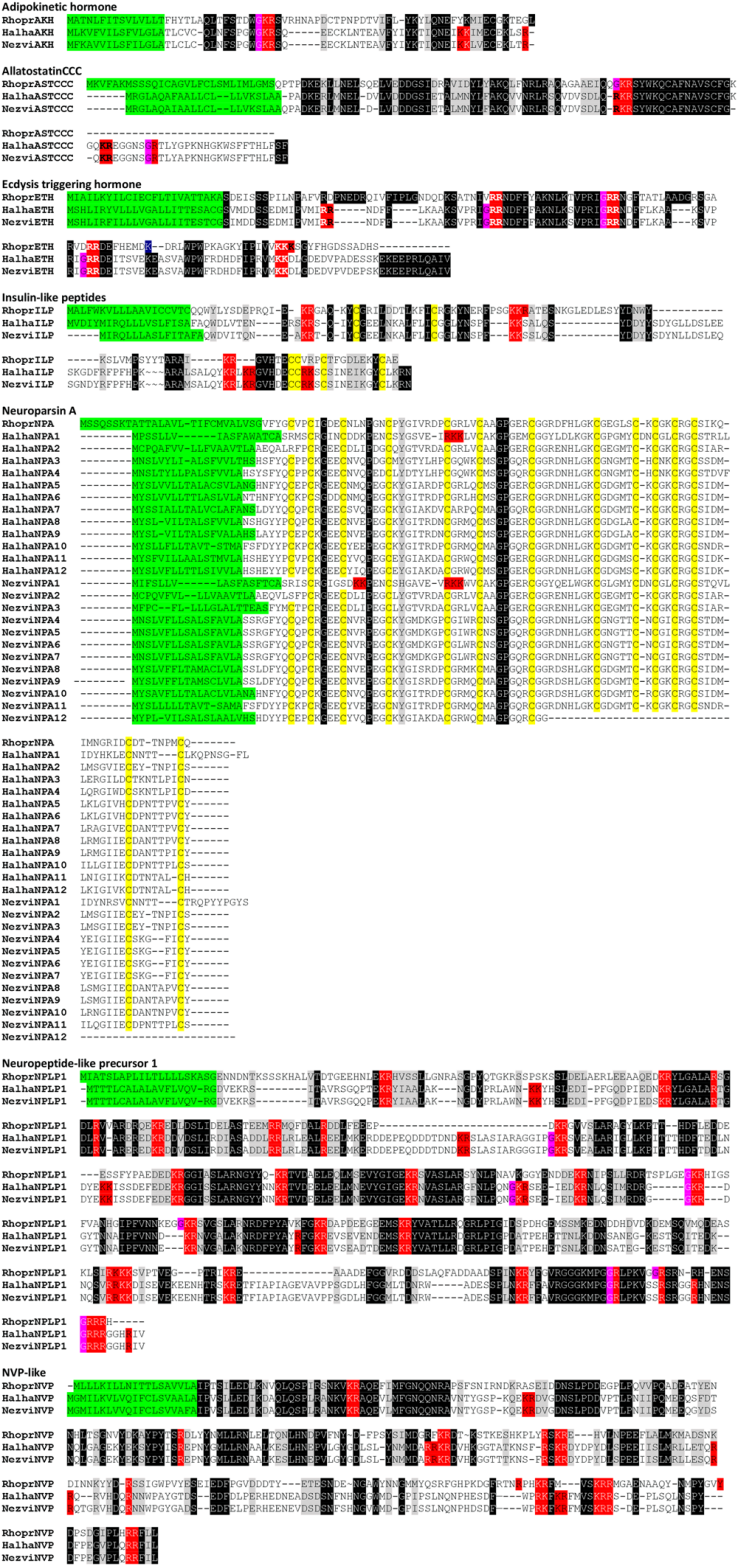
Multiple sequence alignment of neuropeptide precursors that are specific to *N. viridula* and *H. halys*. The sequences of *R. prolixus* were used as a reference. Predicted convertase cleavage sites, according to the rules proposed by Veenstra^84^, are shadowed in red. Glycine residues shadowed in pink indicate predicted amidation sites. The green shadows indicate the predicted signal peptides. The peptides found by mass spectrometry and deduced from transcriptomic sequences are underlined. Black background indicates a fully conserved residue, gray background indicates a conservative substitution. Conserved cysteine residues in neuroparsin A and insulin-like peptides are shadowed in yellow. In Insulin-like peptide, B chain, C peptide and A chain are boxed.

**Figure 2:**
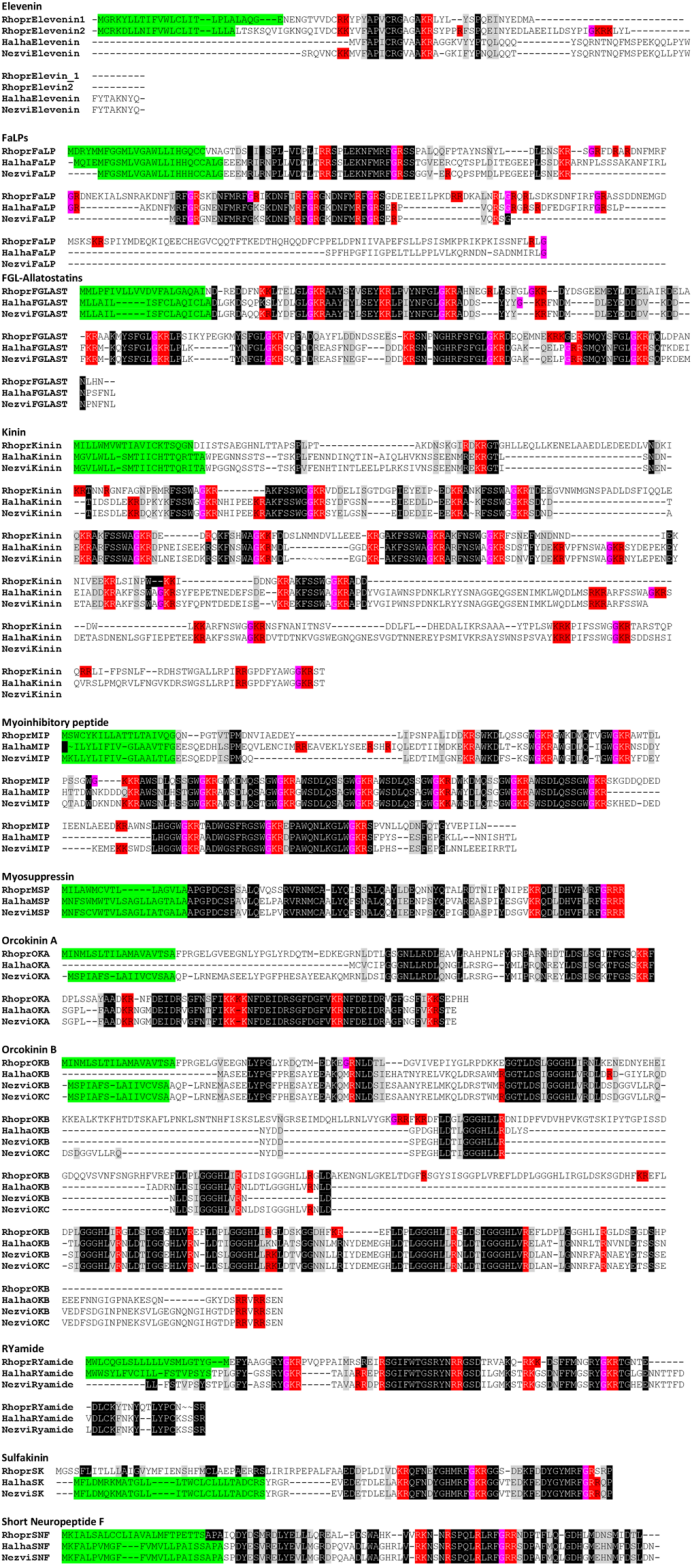
Multiple sequence alignment of neuropeptide precursors that are specific to the heteropteran species analyzed here. The sequences of *R. prolixus* were used as a reference. Predicted convertase cleavage sites, according to the rules proposed by Veenstra^70^, are shadowed in red. Glycine residues shadowed in pink indicate predicted amidation sites. The green shadows indicate the predicted signal peptides. The peptides found by mass spectrometry and deduced from transcriptomic sequences are underlined. Black background indicates a fully conserved residue, gray background indicates a conservative substitution.

In the *N. viridula* adult transcriptome we identified a total of 55 neuropeptide precursor and protein hormone transcripts belonging to 38 conserved families (Table 1; Figs 1, 2; Supp. Info. 5). In *H. halys* genome we identified 55 neuropeptide precursor and protein hormone genes, belonging to 42 families (Table 1; Figs 1, 2; Supp. Info. 5). These numbers are within the range of precursor genes annotated in the genomes of the hemipterans *R. prolixus* and *Cimex lectularius*, 42 and 46 respectively^14,26–28^. Table 1 lists the neuropeptide precursor and protein hormone genes found in *N. viridula*, *H. halys*, *R. prolixus*, *Drosophila melanogaster* (Diptera), *Bombyx mori* (Lepidoptera) and *Tribolium castaneum* (Coleoptera).

**Table 1:** Neuropeptide precursors and protein hormones detected in *N. viridula, H. halys, R. prolixus, B. mori, D. melanogaster* and *T. castaneum*.

	Accession Number in N. viridula	Accession Number in H. halys	R. prolixus	B. mori	D. melanogaster	T. castaneum
Neuropeptides						
ACP	MH311621	XM014430144.1	+	+	ND	+
Adipokinetic hormone	MH311622	XM014430144.1	+	+	+	+
Allatotropin	MH311660	XM014419360.1	+	+	ND	+
AST-CC	MH311623	XM014428577.1	+	+	+	+
AST-CCC	MH311668	JMPT02006736.1*	+	+	+	+
Bursicon alfa	MH971162	JMPT02005569.1*	+	+	+	+
Bursicon beta	MH971161	JMPT02000458.1*	+	+	+	+
Calcitonin-like diuretic hormone	MH311670	XM014417396.1	+	+	+	+
CAPA	MH311661	XM014428750.1	+	+	+	+
CCH-amide 1	MK105809	XM014438492.2	+	+	+	+
CCH-amide 2	MH311624	JMPT02000454.1*	+	+	+	+
CNM-amide	MH311669	XM024363300.1	+	ND	+	+
Corazonin	MH311625	XM014418652.2	+	+	+	ND
CRF like Diuretic Hormone A	MH311626	XM014427687.1	+	+	+	+
Crustacean Cardioactive peptide	MH311628	XM014429289.2	+	+	+	+
Eclosion hormone	ND	XM024358527.1	+	+	+	+
Elevenin	MH311662	XM024361019.1	+	ND	+	+
Ecdysis triggering hormone	MH311629	XM014420230.1	+	+	+	+
FLP	MH311667	XM024363450.1	+	+	+	+
FGL-amide AST	MH311630	XM014426897.2	+	+	+	ND
GPA2	MH971163	JMPT02001085.1*	+	+	+	+
GPB5	ND	JMPT02002909.1*	+	+	+	+
IDLSRF-like	MH311664	XM014434607.2	+	+	+	+
Inotosin	ND	ND	ND	ND	ND	+
Insect kinin	MH311631	XM014419897.1	+	+	+	ND
Insulin-like peptide	MH311633	XM014424698.1	+	+	+	+
Ion Transport peptide	MH311634	XM014418993.2	+	+	+	+
ITG-like	MH311635	XM014420270.2	+	+	+	+
Long Neuropeptide F	MH311636	XM014433721.2	+	+	+	ND
Myoinhibitory peptide	MH311637	XM014421349.2	+	+	+	+
Myosuppressin	MH311638	XM024358266.1	+	+	+	+
Natalisin	ND	JMPT02002493.1 *	+	+	+	+
Neuroparsin A1	MH311648	XM014435550.1	+	+	+	+
Neuroparsin A2	MH311643	XM014435546.2	ND	ND	ND	ND
Neuroparsin A3	MH311642	JMPT02001180.1*	ND	ND	ND	ND
Neuroparsin A4	MH311666	JMPT02001180.1*	ND	ND	ND	ND
Neuroparsin A5	MH311646	JMPT02001180.1*	ND	ND	ND	ND
Neuroparsin A6	MH311647	JMPT02002413.1*	ND	ND	ND	ND
Neuroparsin A7	MH311644	XM014424016.1	ND	ND	ND	ND
Neuroparsin A8	MH311645	XM014424022.1	ND	ND	ND	ND
Neuroparsin A9	MH311640	XM014424021.1	ND	ND	ND	ND
Neuroparsin A10	MH311641	XM014424020.1	ND	ND	ND	ND
Neuroparsin A11	MH311639	JMPT02001180.1*	ND	ND	ND	ND
Neuroparsin A12	KF774301.1	JMPT02001180.1*	ND	ND	ND	ND
Neuroparsin A13	MH311665	ND	ND	ND	ND	ND
Neuropeptide-like precursor 1	MH311649	XM014421103.2	+	+	+	+
Neuropeptide-like precursor 2–4	ND	ND	ND	ND	+	ND
NVP-like	MH311650	XM014434290.2	+	ND	ND	+
Orcokinin A	MH311651	XM014424872.1	+	+	+	+
Orcokinin B	MH311652	XM014424873.2	+	+	+	+
Orcokinin C	MH311653	ND	+	ND	ND	ND
Pigment dispersing factor	MH311654	XM024359646.1	+	+	+	ND
PTTH	ND	ND	ND	+	+	+
Pyrokinin	ND	XM014434450.1	+	+	+	+
Proctolin	MH311655	XM014427746.1	+	+	+	+
RYamide	MH311663	XM014420545.2	+	+	+	+
Sex peptide	ND	ND	+	ND	+	ND
Short Neuropeptide F	MH311656	XM024364219.1	+	+	+	+
SIF-amide	MH311657	XM024359063.1	+	+	+	+
Sulfakinins	MH311658	XM014419008.1	+	+	+	+
Tachykinins	MH311659	XM024361213.1	+	+	+	+
Trissin	ND	ND	ND	+	+	+

ND: no detected; *contig number in *H. halys genome*.

Below we describe and discuss the particular characteristics of neuropeptide precursor genes and protein hormone identified in *N. viridula* transcriptome and *H. halys* genome, in comparison to the hematophagous heteropteran *R. prolixus*, whose neuroendocrine system has been extensively studied (for a review see^14^). A detailed description of the known physiological role of neuropeptides in hemipterans has been discussed in recent publications^14,29–31^. For the analysis, we grouped genes according to their structural particularities: (a) those with unique features in the pentatomids (Fig. 1); (b) those that are specific to *R. prolixus*, *N. viridula* and *H. halys* with respect to other insect species, probably reflecting conserved features in Heteroptera (Fig. 2); and (c) those that are highly conserved compared to most insect species (Supp. Info. 5).

### Neuropeptide precursor genes with specific characteristics in *H. halys* and *N. viridula*

#### AKH

NezviAKH and HalhaAKH precursors encode the core peptide pQLNFSPGW-amide, which was previously sequenced by mass spectrometry^22,24^, and has been reported as characteristic of Pentatomidae^22^, different to RhoprAKH with the sequence pQLTFSTDW-amide (Fig. 1).

#### AST triple C

The arthropod genomes have one to three genes encoding AST-C paralogue genes, and some chelicerates have even more^32^. The existence of three AST-C type paralogues was first detected in arthropods by J.A. Veenstra in 2016^32^. The author classified the three paralogues according to their conserved core peptide in AST-C (or PISC-AST), AST double C and AST triple C^32^. According to this classification, the characteristic of AST triple C is the core peptide SYWKQCAFNAVSCFamide^32^. These molecules have been detected in hemipterans, even though in some cases (such as RhoprASTCCC, TriinASTCCC and NilluASTCCC) they are not predicted to be amidated^20,28,31,33,34^.

For most insect genomes and transcriptomes, except for *L. migratoria* that has all the three^35^, two ASTC paralogues were reported^32^. Whereas PISCF-AST and double C paralogues have been described in Diptera, Coleoptera, and Lepidoptera^32^, double and triple C-type paralogues were reported in most Hymenoptera^32,36,37^ and in Hemiptera^20,28,31,33,34^. It is interesting to note that AST triple Cs in hemipterans have been classified as PISCF-ASTs in the literature, given that the detection of the three types of AST-C paralogues was reported later than the neuropeptidomic analysis for some species. However, according to the taxonomy proposed with the detection of three paralogues in arthropods^32^, they would be better categorized as AST triple C.

As was described in other Hemiptera^20,28,31,33,34^, we found AST double C and AST triple C in *N. viridula* transcriptome and *H. halys* genome. RhopASTCCC, HalhaASTCCC and NezviASTCCC are conserved throughout the sequence of the precursor. However, the core peptide of pentatomids differs in the C-terminus (the most conserved region) from all the AST triple C reported to date^32^ (Fig. 1). As in other hemipterans, this peptide is not predicted to be amidated. Furthermore, in the pentatomids the precursors themselves are extended in the C-terminus. AST-Cs and somatostatin (its orthologue in vertebrates) precursors contain a single copy of the core peptide at C-terminal end^32^. Both NezviASTCCC and HalhaASTCCC precursors are C-terminal extended after the core peptide, with a sequence EGGNSGRTLYAPKNHGKWSFFTHLFSF (Fig. 1), which is not conserved in insect genomes. Hence, ASTCCC in pentatomids have significant differences with respect to their orthologues.

#### Ecdysis triggering hormone (ETH)

In most insect species, including *R. prolixus*, the ETH precursor encodes one or two paracopies of the core peptide^38^. A remarkable characteristic in HalhaETH and NezviETH is that they encode three core ETH neuropeptides (Fig. 1).

#### Neuroparsins (NPA) and insulin-like peptides (ILPs)

**Figure 3:**
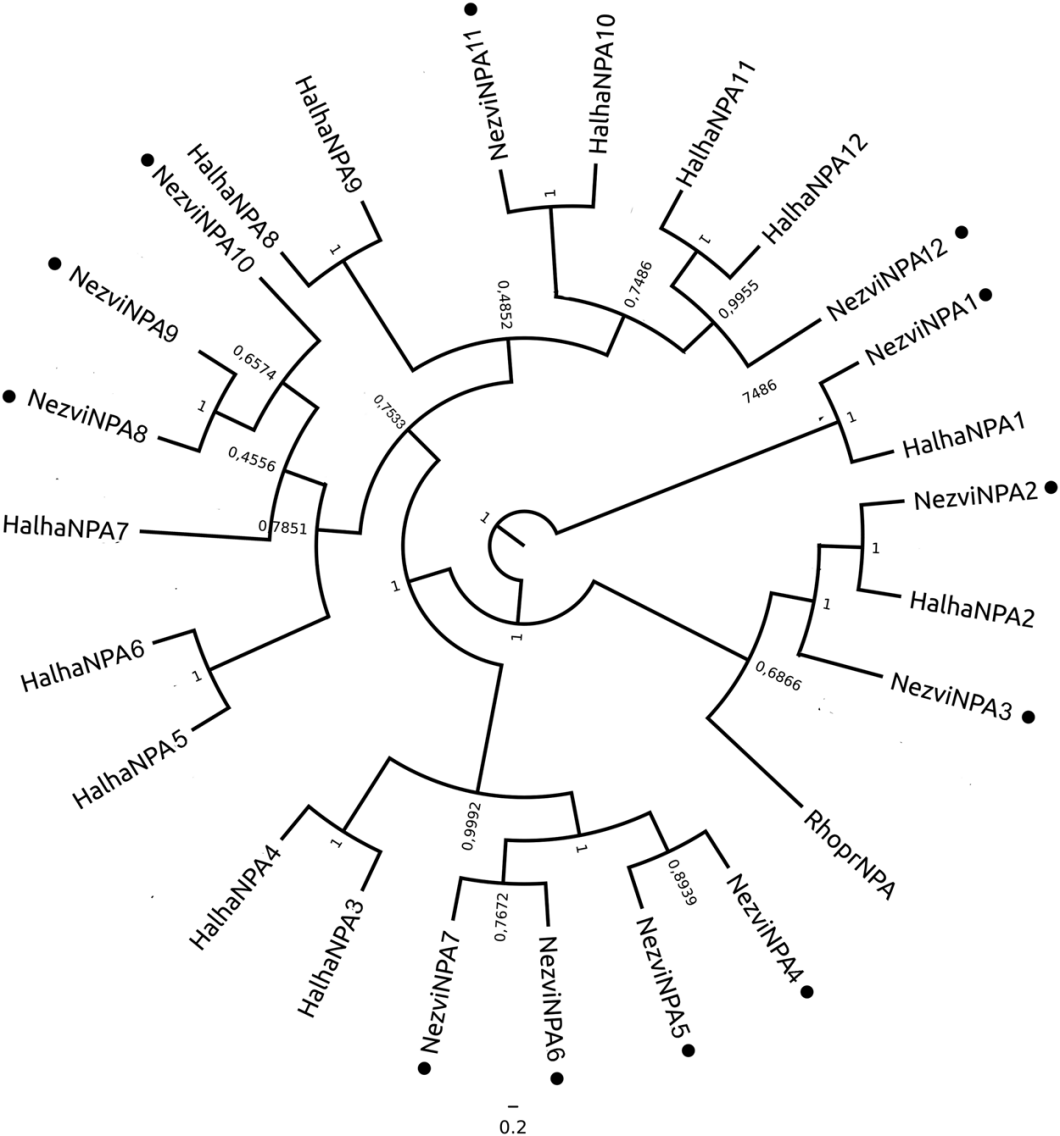
Bayesian phylogenetic analysis of neuroparsin A precursors from *R. prolixus*, *H. halys* and *N. viridula*. *N. viridula* transcripts are indicated with a black circle. The scale bar represents genetic distance. The number at each node indicates the posterior probabilities.

Both in *R. prolixus* genome and in most insect genomes sequenced to date, only one NPA gene exists, even though the recently published *C. lectularius* genome encodes four NPA paralogues^27^. An interesting finding of our analysis is that the genome of *H. halys* possesses twelve paralogue NPA genes (Fig. 1). Nine of them are encoded in the same contig (number 1180), indicating that this expansion is due to gene duplications. Besides, an expansion in NPA gene was also suggested by *N. viridula* transcriptomic analysis, indicating that this could be a particularity of Pentatomidae. For *N. viridula*, 11 complete and 1 partial open reading frames (ORFs) encoding NPA precursors were detected (Fig. 1). One of them has been previously cloned and sequenced^5^. Phylogenetic analysis indicated orthologies among the different members of this gene family (Fig. 3). The expansion of several neuropeptide gene families, such as AST-C and AKH^32,39^, occurs in insect genomes, but the expansion in NPA gene family is unusual given the number of paralogue genes detected (with 12 members/genome). The size of these expansions is comparable to the expansion of ILP gene family in *B. mori*^40,41^ (32 gene copies in the genome). Conversely, only one gene encoding ILPs was detected as both in *N. viridula* transcriptome and in *H. halys* genome (Fig. 1). This represents a reduction, compared with most insect genomes.

In a recent paper, J.A. Veenstra^42^ observed an inverse correlation in the numbers of ILP and NPA paralogues in a given genome. Those species having several insulin paralogues (such as *D. melanogaster*) usually possess zero or one NPA gene in their genome. Conversely, those species having several NPA transcripts, such as decapods and *Locust spp*., have a small number of ILPs. The results presented here support this observation, which suggests a complementation between both hormonal systems. Interestingly, ILPs and NPAs are one of the few neuropeptides in insects acting on tyrosine kinase receptors^43,44^.

#### NPLP1

**Figure 4:**
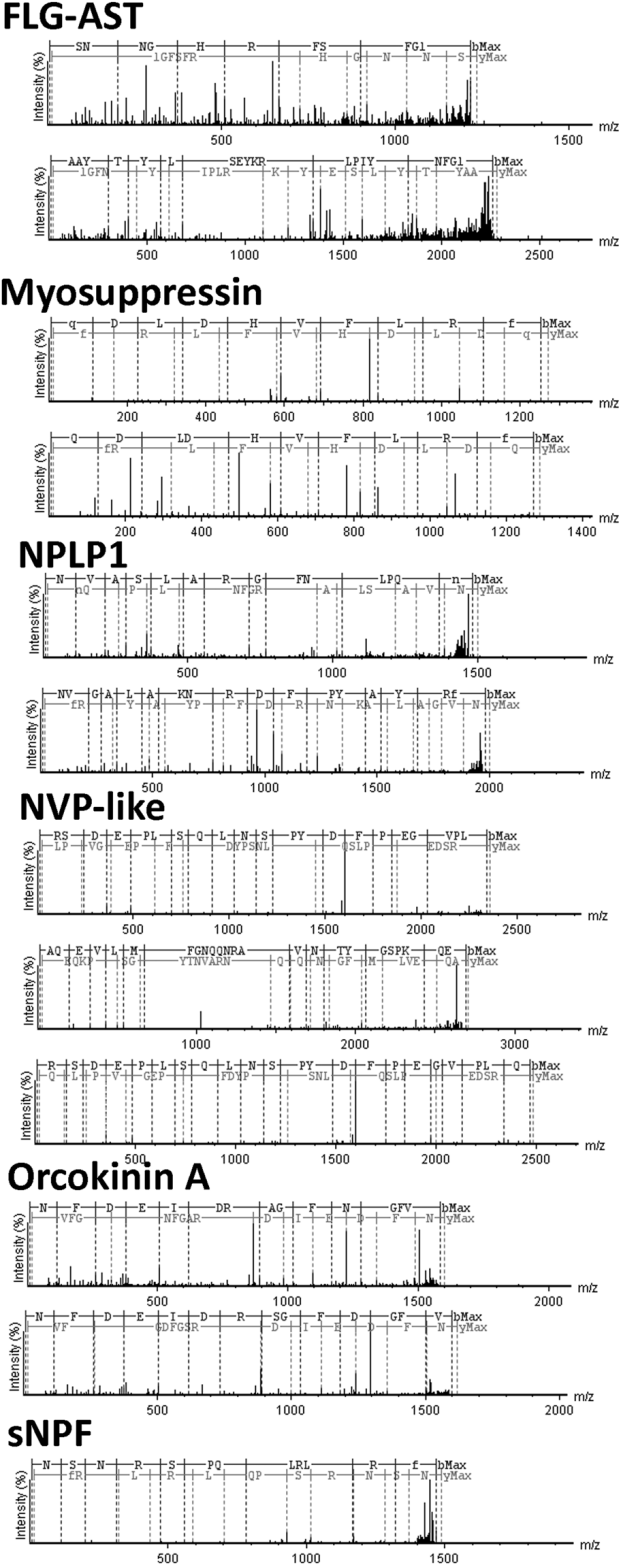
Mass spectrometry spectra verifying the presence of neuropeptides encoded in the precursors described, in brain extracts from male and female adult *N. viridula*.

HalhaNPLP1 and NezviNPLP1 are highly conserved, encoding 22 putative mature neuropeptides, from which three are predicted to be amidated. Two of the amidated peptides could be detected in *N. viridula* brain extracts by mass spectrometry (Fig. 4), suggesting that they would be more stable than the unmodified ones. As in other insect species, NPLP1 paracopies do not present a conserved motif among them. Furthermore, no high conservation with their orthologue in *R. prolixus* was observed, with the exception of NPLP1-4 and NPLP1-10 (Fig. 1).

#### NVPamide

The conservation between RhoprNVP-like precursor and its orthologues in *N. viridula* and *H. halys* is low, even in the region of the core peptides (Fig. 1). We detected two peptides encoded in NezviNPV-like precursor by mass spectrometry. One of them was found either complete or truncated in one C-terminal residue (Fig. 4).

### Neuropeptide precursor features that are specific to *R. prolixus, N. viridula* and *H. halys*

Previous neuropeptidomic studies in *R. prolixus* indicated that several neuropeptide precursor genes are unique in their conserved domains with respect to other insect species^14^. Here, we found that many of these particular domains are also present in *N. viridula* and *H. halys*, suggesting that they could be characteristic of Heteroptera.

#### Elevenin

Different to *R. prolixus*, which has two, *H. halys* possess one gene encoding elevenin. Besides, only one NezviElevenin gene was detected (Fig. 2).

#### FMRDFamide

NezviFMRFamide seems to be incomplete in the transcriptome, when compared to HalhaFMRFamide and RhoprFMRFamide (Fig. 2). Most of the peptides encoded in these precursors have the usual sequence FMRF-amide. Four peptides encoded in RhoprFRMFamide precursor have an infrequent FIRF-amide C-terminal domain^25,28^. This domain is also present in one of the peptides encoded in HalhaFIRF-amide precursor, and in some of the peptides encoded in CimleFMRFamide precursor^37^, indicating that this domain is extended in Heteroptera.

#### FGL-AST

NezviFGL-AST and HalhaFGL-AST encode six core peptides each. As in RhoprFGL-AST^25^, the first HalhaFGL-AST in the precursor has the unusual C-terminal sequence LGL-amide (LTELGL-amide in *R. prolixus*; DLGKDSQPKSLYDLGL-amide in *H. halys*), instead of the conserved FGL-amide, which is present in all the paracopies of NezviFGL-AST and in most insect FGL-ASTs (http://neurostresspep.eu/diner/seqlogopage?neuropeptideID=11). Besides, NezviFGL-AST and HalhaFGL-AST have a putative amidated peptide with sequence DGAKQELP-amide, which is not conserved in other species (Fig. 4). We confirmed the expression of two core NezviFGL-AST/ASTs in the brain by mass spectrometry (Fig. 4).

#### Kinin

For most insect kinin neuropeptides, the C-terminal FXXWG-amide is well conserved (http://neurostresspep.eu/diner/seqlogopage?neuropeptideID=7)^45^. RhoprKinin precursor encodes the conserved core peptides, and five peptides with the unusual C-terminal pentapeptide FSXWA-amide^20,46^. NezviKinin as HalhaKinin precursors encode both kinds of core peptides, suggesting that the FSXWA-amide could be extended in Heteroptera (Fig. 2).

#### Myoinhibitory peptide (MIP)

MIPs were originally characterized by the core C-terminal motif W(6×)W-amide. However, MIP precursors in *R. prolixus* and other hemipterans encode both peptides with the conserved motif, and other paracopies with the unusual motif W(7×)W-amide^20,28,31,34^. Our analysis revealed that NezviMIP and HalhaMIP have six W(6×)W-amide and other six W(7×)W-amide each (Fig. 2).

#### Myosuppressin (MS)

In the triatomines *R. prolixus* and *Triatoma infestans*, the MS precursor is unusual among their orthologues. It possesses an Ile in the third position, instead of the usual Val, and the sequence FMRF-amide in the C-terminus instead of the more conserved FLRF-amide^20,31^. In *N. viridula* and *H. halys*, the third residue of MS neuropeptide is also particular (Leu), but the C- terminal is FLRF-amide (Fig. 2). This indicates that FMRF-amide C terminal could be restricted to triatomine MSs. Using mass spectrometry, we detected the NezviMS core peptide with two kinds of post-translational modifications: C-terminal amidation and N-terminal pyroglutamic and C-terminal amidation (Fig. 4). MS was also detected in triatomines in these two modified forms^19,20,47^; this fact could indicate that both forms have a physiological role, or that a stable intermediate form is being detected by tandem mass spectrometry. Until now, the reported functional analysis with RhoprMS has been performed using the synthetic pyroglutamated peptide^48–50^; bioassays using the non-pyroglutamated MSs would be necessary in order to clarify this point.

#### Orcokinins (OK)

Like RhoprOK, NezviOK gene is expressed in three splicing variants: two of them encoding OKB neuropeptides (NezviOKB and NezviOKC) and another encoding OKA (NezviOKA) neuropeptides (Fig. 2). RhoprOK, HalhaOK and NezviOK precursors are highly conserved. Two mature neuropeptides encoded in *NezviOKA* gene were detected by mass spectrometry (Fig. 4).

#### RYamide

RYamides are conserved neuropeptides in insects, first reported in the parasitic wasp *Nasonia vitripennis*^51^. In *R. prolixus*, the presence of a RhoprRYa precursor has been recently described^14^. NezviRYa and HalhaRYa encode a highly conserved core peptide with the sequence GSDNFFMGSRYamide. Pentatomids also encode another amidated conserved peptide with the sequence FY3(X)RY-amide (Fig. 2) that is less usual when compared with the RYa sequence in most insect species (http://neurostresspep.eu/diner/seqlogopage?neuropeptideID=41). Besides, the three precursors have a conserved non-amidated peptide with the sequence SGIFWTGSRYN, which is also present in *Triatoma dimidiata*^31^. The phylogenetic conservation could suggest a relevant physiological role.

#### Sulfakinin

Like RhoprSK precursor, NezviSK and HalhaSK precursors encode two conserved sulfakinin (SK) neuropeptides. In the three species, the SK precursor encodes the frequent C-terminal sequence GHMRF-amide, and another peptide with the unusual GYMRF-amide. The presence of a conserved and unusual SK was also observed in *C. lectularius*^28^, indicating that this could be a characteristic of Heteroptera. In several insect species, including *C. lectularius*, SK wasfound to be sulfated in a Y residue located N-terminal to the core sequence^28,52^. Hence, Heteroptera SKs would have two potential targets for sulfation. The particular Y in the GYMRF-amide core SK from Heteroptera has not been found to be sulfated in tandem mass spectrometry studies reported to date^19,28^. However, Predel *et al*.^28^ observed weak ion signals supporting sulfation of both Y residues in *C. lectularius* by MALDI-TOF mass spectrometry.

#### Short neuropeptide F

RhoprSNPF, NezviSNPF and HalhaSNPF precursors are shorter than most of their orthologues in insects described to date. The mature peptides predicted in RhoprSNF, NezviSNF and HalhaSNF are highly conserved (Fig. 2). We were able to detect the core NezviSNF by mass spectrometry (Fig. 4).

### Highly conserved neuropeptide precursor genes

#### ACP

NezviACP and HalhaACP precursors are probably incomplete in our predictions, given that a signal peptide in their N-terminal region is absent (Supp. Info. 5). However, the core peptides are complete and highly conserved compared to RhoprACP^53^ (Supp. Info. 5).

#### Calcitonin-like diuretic hormone (CT-DH)

Only one isoform of CT-DH was detected in *N. viridula* transcriptome and predicted in *H. halys* genome, even though three isoforms were reported in *R. prolixus*^20,54^. These precursors encode an identical 31 residue bioactive peptide, which is highly conserved among insect species.

#### CAPA and pyrokinin

Unlike *R. prolixus* genome, which encodes two CAPA paralogue genes^55^, *N. viridula* and *H. halys* seem to have a unique CAPA peptide precursor. In the three species analyzed, these precursors encode two CAPA and one PK peptide (Supp. Info. 5). Although we could not identify the PK precursor in the transcriptome of *N. viridula*, Predel *et al*.^24^ reported a NezviPK mature peptide that is not the one encoded in NezviCAPA precursor, indicating that NezviPK would be present in the genome. Furthermore, a HalhaPK precursor gene was detected in the genomic sequence (Supp. Info. 5).

#### CCHamide

Like other species, *R. prolixus* possesses two *CCHamide* precursor genes in its genome (RhoprCCHamide1 and RhoprCCHamide2)^56^, even though only RhoprCCHamide2 has been identified in its whole lenght^20^. The automatic prediction revealed one HalhaCCHamide gene (CCHAa 1), but we were able to detect also a fragment of HalhaCCHa2 in the contig number 454. As much NezviCCHa1 as NezviCCHa2 transcripts are present in the transcriptome; both possessing a highly conserved core peptide (Supp. Info. 5).

#### CNMamide

NezviCNMa and HalhaCNMa were found in the databases. The precursors identified lack signal peptide, suggesting that the sequences are incomplete in their N-terminal region. The conservation between RhoprCNMa and the pentatomids is low, except for the ASYMSLCHFKICNM-amide core peptide, which is identical in the three species analyzed here.

#### CRF-like diuretic hormone (CRF-DH)

In most insect species, a single isoform of *CRF-DH* gene exists. However, in moth, beetles and *Schistocerca gregaria*, this gene presents splicing variants^57^. Our analysis detected two variants for *NezviCRF-DH* (Supp. Info. 5), a fact that was not reported previously in hemipterans. The isoforms differ in their 3′ region; the ORF of one isoform is 303 bp shorter than the other, not affecting the predicted bioactive peptide (Supp. Info. 5). The predicted mature peptides encoded in NezviCRF-DH and HalhaCRF-DH are both 44 amino acids in length, whereas the one in RhoprCRF-DH is 46 amino acids long^20,58^.

#### Tachykinin (TK) and natalisin (NTL)

NezviTK and HalhaTK precursors encode seven core TK peptides (Fig. 4). HalhaTK precursor is around 50 amino acids longer in its N terminal region compared with NezviTK and RhoprTK (Supp. Info. 5). This does not seem to be due to an error in the predictions or in the assemblies, given that the three precursors encode a signal peptide.

*Natalisin* was not detected in *N. viridula* transcriptome, and could only partially be reconstructed from *H. halys* genome (Supp. Info. 5). The structure of the core neuropeptides in HalhaNTL is conserved compared with *R. prolixus* and other species^59^.

Other neuropeptide precursor genes identified in *N. viridula* transcriptome and *H. halys* genome were ASTCC, allatotropin, crustacean cardioactive peptide, corazonin, eclosion hormone (only in *H. halys*), IDLSRF-like peptide, ITG-like, long neuropeptide F, SIFamide, pigment dispersing factor and proctolin (Supp. Info. 5). Ion transport peptide (ITP) is usually processed in different splicing variants in insects, including *R. prolixus*^20^. We detected only one isoform (ITPB) in *N. viridula* transcriptome (Supp. Info. 5). Likewise, the orthologue of RhoprITPA seems to be absent in the *H. halys* genome. We also detected the glycoprotein hormones NezviGPA2, HalhaGPA2, HalhaGPB5 and the protein hormones NezviBurs alfa, NezviBurs beta, HalhaBurs alfa and HalhaBurs beta (Supp. Info. 5). All these genes seem to be highly conserved in pentatomids compared to their orthologues in other insect species (http://neurostresspep.eu/diner/insectneuropeptides).

### Neuropeptides not detected

EH, PK and GPA2 are highly conserved in insect genomes. The three of them were detected in *H. halys* genome but not in *N. viridula* transcriptome, probably due to the incompleteness characteristic of transcriptomes when compared to genomes.

The existence of a basal and variable set of neuropeptides in insects has been proposed^51^. The basal set would reflect the involvement in survival-related processes, whereas the variable set would regulate specialized events related to particular adaptations. Even though NTL does not belong to the basal set, its presence in *H. halys* genome suggests that it should also be present in *N. viridula*, although it was not detected in the transcriptome. The remaining components of the variable set (inotosin, NPLPs 2–4, sex peptide, trissin and PTTH) have not been identified in triatomine databases to date^14,31^, even though PTTH has been reported in other hemipteran species such as *N. lugens*^33^ and *A. pisum*^60^, and inotosin was detected in *N. lugens*^33^. We did not detect these precursors in our genomic, transcriptomic or peptidomic approaches.

### Genes encoding G-protein coupled receptors for neuropeptides in *N. viridula*

**Figure 5:**
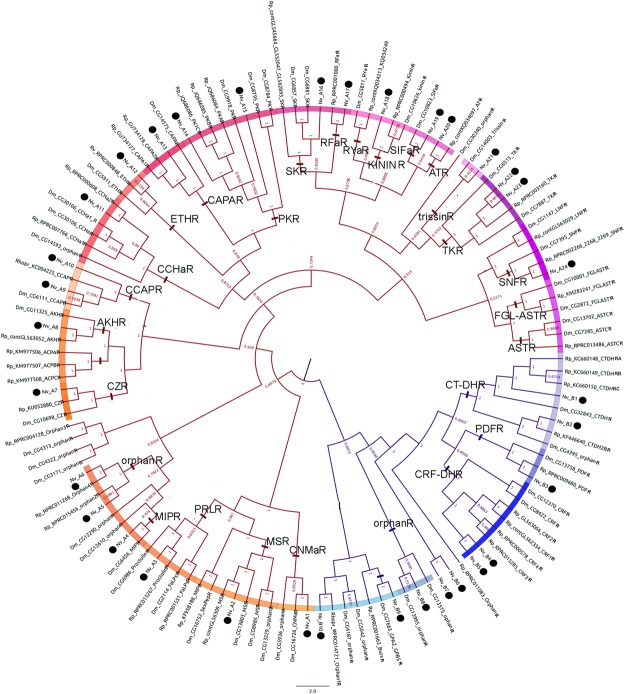
Bayesian phylogenetic analysis of GPCRs for neuropeptides and protein hormones from *D. melanogaster* (Dm), *R. prolixus* (Rp) and N. viridula (Nv). *N. viridula* transcripts are indicated with a black circle. The scale bar represents genetic distance. The accession number in FlyBase is indicated for *D. melanogaster* GPCRs; for *R. prolixus*, either GeneBank accession number, contig number or transcript number in vectorbase/VectorBase (www.vectorbase.org) are indicated (for reconstructed R. prolixus GPCRs see predicted sequences in^14,18,14,18^). Family A GPCRs are indicated by red branches; family B GPCRs are indicated by violet branches. Each GPCR family is indicated by different color shadows. The names of the receptors are indicated in the base of each clade. The scale bar represents genetic distance. The number at each node indicates the posterior probabilities.

A total of 34 transcripts encoding putative GPCRs for neuropeptides and protein hormones were identified in the *N. viridula* transcriptome (see sequences in Supp. Info. 6). From them, 24 belong to family A and 10 belong to family B GPCRs (Fig. 5). Possible ligands of these GPCRs were deduced by phylogenetic analysis and sequence similarity, comparing with the GPCRs for neuropeptides and protein hormones in two species were this gene family has been well-studied (for reviews see^14,18,61,62^): *D. melanogaster* and *R. prolixus*. Most of these receptors have been functionally deorphanized and characterized in both species^53,56,63–75^, whereas RhoprASTCR, RhoprATR, RhoprPDFR, RhoprBursR, RhoprFalpsR, RhoprPrlR, RhoprSKR and RhoprRFaR are phylogeny-based predictions^14,18^. A clear orthologue could be assigned for most of the GPCR transcripts identified in *N. viridula* transcriptome (Fig. 5).

For family A GPCRs, phylogenetic analysis allowed the identification of CNMamide-R (NvA1), MS-R (NvA2), Proctolin-R (NvA3), MIP-R (NvA4) CRZ-R (NvA7), AKH-R (NvA8), CCAP-R (NvA9 and NvA10), CCHamide-R (NvA11), ETH-R (NvA12), CAPA-R (NvA13), PK-R (NvA14 and NvA15), SK-R (NvA16), RFamide-R (NvA17), kinin-R (NvA18), SIFamide-R (NvA19), AT-R (NvA20), TK-R (NvA22 and NvA23), SNF-R (NvA24) (Fig. 5). The transcripts NvA5 and NvA6 encode family-A GPCRs that are grouped with orphan receptors from *R. prolixus* and *D. melanogaster* (Fig. 5). The transcript NvA21 is closely related to DromeTrissin-R; considering that trissin neuropeptide has not been detected in Hemiptera to date (including the present results), we propose that NvA21 could be a TK-R, given its sequence and configuration in the phylogenetic analysis (Fig. 5).

Regarding family B GPCRs, 2 orthologues were identified for CTDH-R (NvB1 and NvB2; closely related to RhoprCTDH-R1 and RhoprCTDH-R2 respectively), 1 for PDF-R (NvB3) and two were related to CRFDH-R (NvB4 and NvB5) (Fig. 5). One *N. viridula* GPCR (NvB9) seems to be the orthologue of DromeGPA2/GPB5 GPCR (Fig. 5). Four family B GPCRs from *N. viridula* were classified as orphan receptors (NvB8 and Nv10), or have no clear orthologue in *D. melanogaster* nor in *R. prolixus* (NvB6 and NvB7) (Fig. 5).

### Concluding remarks

The combination of transcriptomics, bioinformatics and peptidomics has produced relevant advances in entomology, particularly regarding neuroendocrine system characterization. Nevertheless, these combined studies have not been used for a comprehensive characterization of the neuropeptidergic complement in pentatomids to date, despite their economic relevance. For *N. viridula*, neither genomic nor transcriptomic information has been made publically available until the present work. Even though *H. halys* has a sequenced genome, neuropeptide precursors were only automatically annotated. Here, we present the analysis of a highly complete *N. viridula* transcriptome *de novo* generated in our laboratory. Furthermore, we performed comprehensive comparisons and analyses of the neuropeptide precursor complement in *N. viridula*, *H. halys* and *R. prolixus*, and identified GPCRs for neuropeptides in *N. viridula*. Several neuropeptide systems are well conserved among the three species analyzed, and many of them are also conserved throughout the class Insecta. However, a few neuroendocrine systems possess characteristics that could be particular for/specific to pentatomids. The most remarkable is the case of NPA, a gene family that was expanded by gene duplications, both in *N. viridula* and in *H. halys*, with 12 NPA-encoding transcripts identified in each database.

The data presented here provide necessary tools for functional studies involving RNA interference, tissue specific gene expression, quantitative peptidomics, etc. Our study provides a promising starting point for physiological studies in pentatomids, oriented to basic entomology as much as to the design of next-generation insecticides based on neuroendocrine targets, which are expected to be species-specific and environmentally friendly.

## Materials and Methods

### Insect rearing

Adult male and female specimens of southern green stinkbug *N. viridula* were obtained from an established colony at Centro de Bioinvestigaciones, Universidad Nacional del Noroeste de Buenos Aires (UNNOBA), Argentina. This colony was originated with insects collected from soy and pea fields in the environs of Pergamino City (33°53′S: 60°34′W; Buenos Aires, Argentina). Insects were reared under controlled temperature (27 ± 2 °C), with a 16 h light:8 h dark photoperiod, and supplied with food (pea or soy beans) and water (soaked cotton wool) *ad libitum*. Tissue paper was provided for egg deposition. Deposited eggs were separated from the adults, and nymphs were divided according to their stage.

### Transcriptome preparation and sequencing

Total RNA was isolated from 10 adult insects-both females and males in the same proportion-using Trizol Reagent (Invitrogen, Carlsbad, CA, USA). A cDNA library was constructed with 1 µg of total RNA and it was barcoded and subjected to the 100 pb pair-end shotgun sequencing using HiSeq. 2000 platform (Illumina) at the Novogene sequencing facility (California, USA). The raw sequence dataset is available at the NCBI-SRA SRR7184294.

### Data filtering, trimming and assembly

Before *de novo* assembly raw reads were processed with FASTX-toolkit software (http://hannonlab.cshl.edu/fastx_toolkit/) to remove those with quality scores lower than 30 and to trim adaptor sequences. In order to avoid contaminants, we tested the persistence of adaptor sequences using BLASTn and the UniVec database (ftp://ftp.ncbi.nlm.nih.gov/pub/UniVec) from NCBI with the following command options: -reward 1, -penalty −5, -gapopen 3, -gapextend 3, -dust yes, -soft_masking true, -evalue 700 and -searchsp 1750000000000.

*N. viridula* dataset was assembled with Trinity-V2.3.2 software package^76^ using a pair-end assembly strategy and 25 bp long kmers. To avoid the inclusion of unique kmers that could possess sequencing errors we used the min_kmer_cov = 2 as minimum coverage parameter. The assembled sequence dataset is available at the NCBI-TSA GGPJ00000000.

Related assembled genes (paralogues or gene fragments) have the same cluster number (_c# coordinate in the transcript identifier) when transcripts are generated in Trinity, where all reads corresponding to a gene would end up in such cluster. In this way, to avoid the potential effects of transcript redundancy in the complete dataset, we built an additional transcriptome dataset for the assessment of statistical representation in later analysis. In this way, a non-redundant dataset (nr_dataset) was created, discarding alternative contigs belonging to the same cluster, and keeping only the largest contig (transcript) per cluster.

### Transcriptome completeness analysis

The assembled transcriptome dataset was used to identify the proportion of the core eukaryotic genome coverage using HMM profiles for 458 core eukaryotic proteins^77^ and HMMER3 searches with the hmmscan command and the -T 40 and–domT 40 filters, as described previously^18^. At the same time, a BUSCO set for arthropod^78^ was used to evaluate transcriptome completeness. To estimate the proportion of reads coded by the mitochondrial genome and to identify mitochondrial-encoded genes, the software Bowtie2^79^ was employed to map raw reads of *N. viridula* transcriptome generated to the *N. viridula* reference mitochondrial genome (GenBank accession: EF208087.1).

As another transcriptomic completeness metric, we used NCBI-BLASTX (-e 1.0E-05) and the non-redundant UNIPROT database (UNIPROTnr db) to evaluate the number of proteins we could identify in the transcriptome comparing with other organisms.

To estimate the proportion of the generated database that is homologous to *R. prolixus* (httpd://www.vectorbase.org/organisms/rhodnius-prolixus/cdc/rproc3;26), *H. halys* (https://i5k.nal.usda.gov/Halyomorpha_halys), and *O. fasciatus* (https://data.nal.usda.gov/dataset/oncopeltus-fasciatus-genome-assembly-10) predicted proteomes, we used NCBI-BLASTX (–e 1.0E-05). Putative 1:1 orthologue identification among these datasets was done using the BLAST RBH strategy as described^80^. The orthologues shared by three or more species were calculated using a bash script to simplify this operation (bash script is provided in Supp. Info. 6).

### GO analysis

Only the transcripts shared among all the hemipterans analyzed above and the orthologues identified in phytophagous insects–*O. fasciatus*, *N. viridula* and *H. halys*–were annotated using Blast2GO (B2G) platform^81^. The annotation was performed using NCBI-BLASTx (e value < 1.0E-3) program inbuilt in the B2G program and InterProScan. Both BLASTx and InterPro searches were merged and mapped with gene ontology (GO) terms, and only the GO terms with an e value lower than 1.0E^−6^, annotation score lower than 55, and GO weight more than 5 were finally annotated. We searched for KEGG database using Blast2GO to distinguish the core pathways in which *N. viridula* presumed proteins are involved.

### Neuropeptide precursor and GPCR gene identification

In order to identify GPCRs and neuropeptide precursors, we performed iterative tBLASTn searches in the *N. viridula* transcriptome using local BLAST^82^. As queries, we used a database including all the family A and Family B GPCRs known in *R. prolixus* and *D. melanogaster* (see Fig. 5) and a database including orthologues from *R. prolixus*, *D. melanogaster*, *T. castaneum*, *B. mori* and/or *Plautia stali* for all the insect neuropeptides. For *H. halys* neuropeptide identification, tBLASTn searches were performed online in the NCBI server (https://blast.ncbi.nlm.nih.gov/Blast.cgi), in *H. halys* whole genome sequence and Nr databases. Aminoacidic sequences of neuropeptide precursors from were used as queries. For the structural analysis of the neuropeptide precursors SignalP3 (identification of signal peptide)^83^ and the rules previously proposed for the prediction of convertase cleavage sites^84^ were used.

For GPCRs, the resulting aminoacidic sequences were used to perform an InterProScan^85^ search using the Gene3d, PfamA and SuperFamily applications to obtain the proteins of interest, which were further analyzed to confirm their identity.

### Phylogenetic analysis

Phylogeny for NPA precursor and GPCR family was based on sequence alignments generated by CLUSTAL Ω^86^, using the software BEAST v1.8.350 in the CIPRES Science Gateway^87^. Beauti v1.8.350 was used to generate the BEAST input files. We used 30 million generations for each run, combined with LogCombiner v1.8.350 discarding the first 10% of each chain as a burn-in. The maximum clade credibility tree was generated using TreeAnnotator v1.8.3^88^. The result was visualized with Figtree and Itol tools^89^.

### Peptide extraction, liquid chromatography-tandem mass spectrometry and peptide identification

Brains from adult male and female *N. viridula* (N = 13) were dissected and immediately pooled in 100 μl cold methanol/water/acetic acid (90, 9, 1, v/v/v). Peptide extractions and desalting were performed as described previously^19^. The resulting peptides were separated by reverse phase nanoflow liquid chromatography (ProXeon EASY-nLC II, Bruker, Bremen, Germany) and spotted onto AnchorChip matrix-assisted laser desorption/ionization targets and analyzed on an UltrafleXtreme MALDI-TOF/TOF mass spectrometer (Bruker, Bremen, Germany). Tandem mass spectrometry spectra were processed using the software msconvert (http://proteowizard.sourceforge.net). For peptide identification we used Peaks Studio 7.0 (Bioinformatic Solutions, Waterloo, ON, Canada) with the same specification used previously^31^. Searches in a database containing the neuropeptide precursor sequences of *R. prolixus*, *H. halys* and *N. viridula* were performed.

## Electronic supplementary material

Supplementary information

Supplementary Dataset 3

Supplementary Dataset 4

## Data Availability

All the data presented are publically available. We provide the GeneBank accession number for each sequence. The raw sequence dataset is available at the NCBI-SRA SRR7184294.
