# Supplementary material for: Nezara viridula (Hemiptera: Pentatomidae) transcriptomic analysis and neuropeptidomics
Source: Sci Rep. 2018 Nov 22;8:17244. doi: 10.1038/s41598-018-35386-4 (PMC6250713; doi:10.1038/s41598-018-35386-4)
Supplement: Supplementary file 1 — Supplementary information [file 41598_2018_35386_MOESM1_ESM.pdf]

Supplementary information for the article: Nezara viridula (Hemiptera: Pentatomidae) transcriptomic analysis and neuropeptidomics

Authors: Andrés Lavore[1], Lucila Perez-Gianmarco[1], Natalia Esponda-Behrens[2], Victorio Palacio[1], Maria Ines Catalano[1], Rolando Rivera-Pomar [1,2] and Sheila Ons[2]*

1- Centro de Bioinvestigaciones. Universidad Nacional del Noroeste de Buenos Aires. Pergamino. Argentina.

2- Centro Regional de Estudios Genomicos. Facultad de Ciencias Exactas. Universidad Nacional de La Plata. Buenos Aires. Argentina.

* Correspondence to: Sheila Ons. Bvd. 120 1459 (1900) La Plata. Buenos Aires. Argentina. Tel: +54 221 423 6332. E-mail: sheila.ons@presi.unlp.edu.ar/ sheilaons@gmail.com

Supplementary information 1

Table s1. Sequencing and de novo assembly metrics	
**Raw sequencing**	
Raw reads	560,886,454
Number of bases (Mpbs)	280.4
Filtered reads (FASTX-Toolkit)	558,876,616 (99,6%)
**Assembly**	
Assembled bases (Mpbs)	193.2 (68,9%)
Isotigs num	299,148
Isogrups num	221,206
Isotig Average Size	646
Largest Isotig Size	15,451
N50 Isotig Size	1,093
% GC	32.53
N. viridula nr-dataset	96,169
**Transcriptome completeness**	
N. viridula mitochondrial genome& coverage (Pb)	16,657 (98.6%)
CEG#	457 (99.5%)
BUSCO+	2,527 (94.4%)
BLASTx vs Uniprot hits	14,375
& ***N. viridula*** **mithochondrial genome in pb: 16,889**	
# **hmmsearch score ≥ 40; n= 458 CEG's.**	
+ **hmmsearch score ≥ 40; n= 2,676 BUSCO's.**	

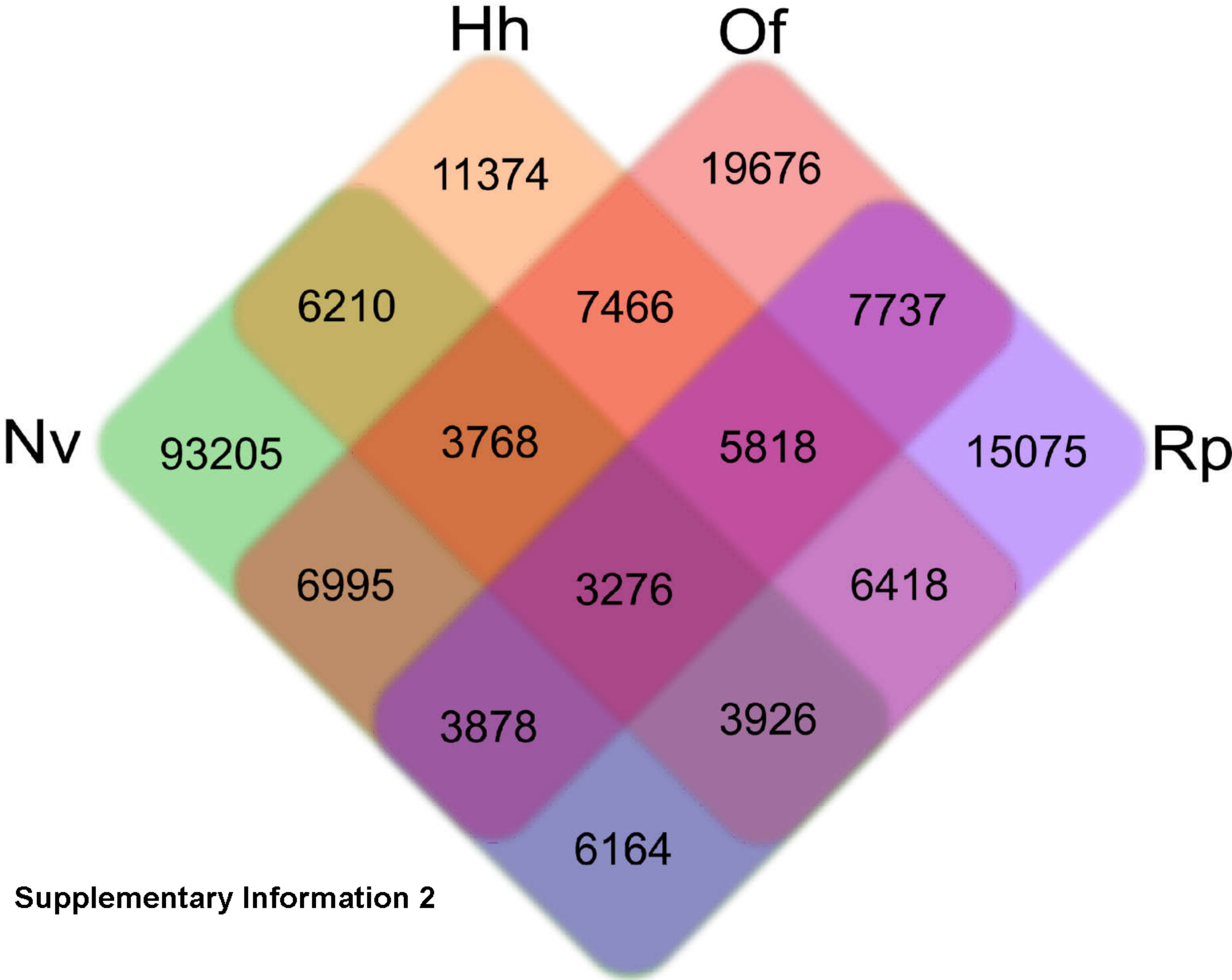

Supplementary Information 2

Figure 5 Supplementary information: Multiple sequence alignment of conserved neuropeptide precursors in *H. halys* and *N. viridula*. The sequences of *R. prolixus* were used as a reference. Predicted convertase cleavage sites, according to the rules proposed by Veenstra[70], are shadowed in red. Glycine residues shadowed in pink indicate predicted amidation sites. The green shadows indicate the predicted signal peptides. Black background indicates a fully conserved residue, gray background indicates a conservative substitution

ACP

```
RhoprACP   -------------------------MGEEDSSQDHHFSIYTGQMDYSSIIFEMDRSKTVRRLSTVALVYLIFINIFLVEAQVTFSRDWNAGKRSNNIPDC
HalhaACP   MSNQLALYPCLYFFPLKRALGVLYKSGTPGNSPSH-------------PDPDMDGRKVATSYAVIFVAFILFQEISNSYAQVTFSRDWNAGKRAVSE-CN
NezviACP   ----------------------------------------------------MDGRKIAASYAVIFATFILFQEIANSYAQVTFSRDWNAGKRSVSE-CS

RhoprACP   AIAIKSAAAICQMLLNELRAIATCEMHSLTSQRLNEDVENSQDVFGSQHNG
HalhaACP   QMSIKSAAAICQMLLGELKSLATCEMRSLLSTRVSEEVDNPPDVFSART-G
NezviACP   QMSIKSAAAICQMLLGELKSLATCEMRSLLSTRVSEEVDNPPDVFSSRT-G
```

Calcitonin-like diuretic hormone

```
RhoprCTDHA   MVTNIAVVGVSLMLGTLIVLSAASENIPYIGHRASYFGDMDNEPDSEVMLEILAKLGRTIMRANDLEKPMIYSREASNPWTAVNKLRPSNLPYNIELAEN
RhoprCTDHB   MVTNIAVVGVSLMLGTLIVLSAASENIPYIGHRASYFGDMDNEPDSEVMLEILAKLGRTIMRANDLEN--------------------------------
RhoprCTDHC   MVTNIAVVGVSLMLGTLIVLSAASENIPYIGHRASYFGDMDNEPDSEVMLEILAKLGQTIMRANDLEKPMIYSREASNPWTAVNKL--------------
HalhaCTDH    MVSNVILVTFSLTLGTLILLSTAAESVPYIGHR-GYLGDLESEPDPEVILEILAKLGQTIMRANDLEN--------------------------------
NezviCTDH    MVSNVILVTFSLTLGTLILLSTAAESVPYIGHRGGYIGDLESEPDPEVILEILAKLGQTIMRANDLEN--------------------------------

RhoprCTDHA   PDSIYSKRGLDLGLSRGFSGSRAAKHLMGLAAANYAGGPGRRRRQA
RhoprCTDHB   -----SKRGLDLGLSRGFSGSQAAKHLMGLAAANYAGGPGRRRRQA
RhoprCTDHC   ----SSKRGLDLGLSRGFSGSQAAKHLMGLAAANYAGGPGRRRRQA
HalhaCTDH    -----SKRGLDLGLSRGFSGSQAAKHLMGLAAANYAGGPGRRRRQA
NezviCTDH    -----SKRGLDLGLSRGFSGSQAAKHLMGLAAANYAGGPGRRRRQA
```

CAPA

```
RhoprCAPA     MSYTVGTILVTAVLVSTCAVINSAEQTNEDKTNTTLRI----KRSPISSVGLFPFLRAGRARNFPATWGMLVGDD-----KN-KREGGFISFPRVGRSGPKRNG
HalhaCAPA     MSNV-FCLVASLFLLVSCSRAAPGQKT-------ISQPCTREKRDA----GLFPFPRVGRT-SFPFTWSFLPLIDPESGERQIKREQL-IPFPRVGKSGPKRNG
NezviCAPA ----NV-FYLVASLFLLLSCSRAAPGLKT-------VSQACTREKRDA----GLFPFPRVGR--TFPLTWSFLPLVEPESGERQIKREQL-IPFPRVGKSGPKRNG

RhoprCAPA     GGGNGGGLWFGPRLGRNQKRGDSWTLEQLQPNLLPGYPAYN----EEEKENQFSEELSDESVSN-KIV
HalhaCAPA     ASGN-GGLWFGPRLGRLNKRMELIPVAYRQENNVPETLLKSVLKNLPLKGAGNSND-VDD-YIDGKQ-
NezviCAPA   ..ASGN-GGLWFGPRLGRLSKRMELVPISYRQENNVPENLL----KNSPVKGIGNSND-IDD-YIDSKQ-
```

Pyrokinin

```
RhoprPK   MVSVSLVG--LLLVALQLITNGCTQGTRRTLSKWHGDNDLTRQEETVMELLKDNPWAHFSVR----EGGRNTVNFSPRLGRDEEVVFTETSRSPPFAPRL
HalhaPK   MAPHCWFSFSLLLITSLISSYGAEED---RVQRAEE------DDDLETAIVGIAPWAAILPISGGERGSRQLVSFRPRLGREEEV-LAESSRSPPFAPRL

RhoprPK   GRIV--FRPRFGRLTLAAQH
HalhaPK   GRFYAPFSPRLGRRRS----
```

CCHamide1

```
HalhaCCHamide1   MHHLYTLLVMATIIASAPTYASGSCLSYGHSCWGAHGKRSGDLQPAQFIVPKANSLSDRQARLTEYLLSLSQLQQQKNPEDAVDDTGREEVILMEQPLRL
NezviCCHamide1   ~HHLYTLLVMASIIASAPTYAAGSCMSYGHSCWGAHGKRSGDLQPSQFIVPKANSLADRQARLTEYLLSLSQLQQQKSPEDTADDIGREEVILMEQPLRL

HalhaCCHamide1   YQILDQLPRSYEGKSSINN
NezviCCHamide1   YQILDQLPRSYEGKSSINN
```

CCHamide2

```
RhoprCCHamide2   -----MICSR-----------KMIVTLLLVSLLLTVHGAAFKGARDGDASFRKKPLRRVFYFLLLSGGCSAFGHSCFGGHGKRSDDYMAQIQSR[illegible]LQRL[illegible]
NezviCCHamide2   MKAPGHLAVILILAIVVFQAFAFPYKGLTHAFLRLVKNSSLTSERDGESSFRKKPLRR--------GGCSSFGHSCFGGHGKRSEDYFSQIRRFQ-KVSP

RhoprCCHamide2   PADIVRQWMKSLHNVPP~LLE*---------------------------------------------
NezviCCHamide2   STDIIRQLLKAYHSSSPSLLE*--------------------------------------------
```

CNMamide

```
RhoprCNMamide  ----MNGK-VWLQFLYEGVSRQVMACAVLLGLLTQVPASSPSSTRTQELQESSPSLQQLISLFDYLQNRELEALASDGTQNDSGGNNGLLESNSNKNRL
HalhaCNMamide  ----LWYIISFVLLIFAGSYGRLLLIAVAMAVAIA-------------APLQEPSLQEALAYINTLHSQEVT----NG------KLRDLYEAL-RRG-
NezviCNMamide  MVSSLNELLQAIGFLLNEGSYRRLLLIAVAMAVLSGAIA----------APLQEPSLQEALAYINTLHSQEVS----NG------KLKDLYEALLRRG--

RhoprCNMamide  RELANAYDELLRQNML--QQSAAQHLSAEKRASYMSLCHFKICNMGRKRTPYEAVHKK
HalhaCNMamide  -GDDIEDEALLQAKIIEYLQTNNLLNKADKRASYMSLCHFKICNMGRKRTLWPRN---
NezviCNMamide  -GEDIEDDALLQAKIIEFLQAHDLQNKADKRASYMSLCHFKICNMGRKRTLWPRN---
```

CRF-like diuretic hormone

```
RhoprCRFDH  MWIKCVWVACLVGAVHGSLQDTRAHNLLQDKWEPPLPPAHNTRYYFIDQPLRPQPPLQESEIDDEVSWRGKRMQRPQGPSLSVANPIEVLRSRLLLEIAR
HalhaCRFDH  MWVKVLL-----AWLLAALALV----AASSDWSQSSS------------------SHLYSPLLQDDEWRGKR----TGPSLSVANPIEVLRNRLLLEIAR
NezviCRFDH  MWVKVLL-----AWLLAALALV----AASSDWSQSSS------------------SHLYSPLLQDDDWRGKR----TGPSLSVANPIEVLRNRLLLEIAR
NezviCRFDH  MWVKVLL-----AWLLAA ALV----AASSDWSQSSS------------------SHLYSPLLQDDDWRGKR----TGPSLSVANPIEVLRNRLLLEIAR

RhoprCRFDH  RRMKEQDASRVSKNRQYLQQIGKRHT--NRLTNEDKT-VNMDYYNSGDFDWDSA-
HalhaCRFDH  RRLQKQG-GQVEQNRAFLQNIGKRHTMPNRSFDRRFLDSSD-YRLSSG-EWESP-
NezviCRFDH  RRLQKQG-GQAEQNREFLKNIGKRHTMPYR-------------------------
NezviCRFDH  RRLQKQG-GQAEQNREFLKNIGKRHTMPYRSFDRRFLDSSD-YRLSSG-EWETP-
```

Tachykinin

```
RhoprTK   ----------------------------MPV--------------------GSLLVMSCVLAACLAQERRAMGFVGMRGKKDTPDMEEYKRAPSTMGFQG
HalhaRK   MSVIISKYIFGLWVAYCHSLVGYKEQLCLPNGVEQVNFKHFTSVRISSRSRRRFSAIECLYNVINKITAIVWAAVAVTVLADVATAQEVRRAPS-MGFMG
NezviTK   -----------------------------------------------------------------MRITAIVWAAVAVTVLADVASAQEVRRAPS-MGFMG

RhoprTK   VRGKKDDLIGEPDDTFLEEFKRAPAAMGFQGMRGKKTPAMGFMGMRGKKDSDYGWWEEDKRAPASGFFGMRGKKAPASGFFGMRGKKGPSSSAFFGMRGK
HalhaRK   MRGKKDIFP--EDDNSIEEFKRAP-LMGFQGVRGKKAPSMGFMGMRGKKEEGQDFWDEEKRAPSSGFFGMRGKKAPAAGFFGMRGKKGPSS-GFFGMRGK
NezviTK   MRGKKDIFP--DDENSIEEFKRAP-LMGFQGVRGKKAPSMGFMGMRGKKDEDQDLWDEEKRAPSSGFFGMRGKKAPAAGFFGMRGKKGPSS-GFFGMRGK

RhoprTK   KGPSGFMGVRGKKDSPDDLNHLLQLLRESALKQEMEEMLEDGRGLKRFAGLSDSFEDYP--AELL
HalhaRK   KGPSGFLGMRGKKESIDDIDSLLQYLRDSEARQDIEDMMETRA--KRFAD--DSFQEPQSEQDFL
NezviTK   KGPSGFLGMRGKKESIDDIDSLLQYLRDSEARQDVEDMMETRA--KRFVD--DSFQEPQSQQDFL
```

Natalisin

```
RhoprNTL  MNPVHTLLVALISVGLQETSSVGEKCDKAVCGEERRSDV-RAVLGSSEAEPGFWPTRGRRGDSSSTEEVQPPFWAHRGREE-RPCDSSSVNSLNHLYAQEP
HalhaNTL  -------------------------------CEERRTDVMRAVL-SGPAEPGFWPSRGRRSDTSS-EEEPPFWAHRGRGLNAPCPGSDLNRY--------

RhoprNTL  KFLLIHRRDTMEQDPFWVSRGRKRSQKSNFAYLEETSLTAGEGGGELITVTNSFAGEMRGLWSLGRLPVQVKSHLNIQYKWHLSLANITNTQPEQVP
HalhaNTL  --LVL-----HQEEPFWLSARRKRSQDH-----------------------------------------------------------------------
```

Allatostatin double C

```
RhoprASTCC  MWLPSA---YISRLSWWCFALVFTTLLAATCVL----TSPAPSYNDYQQVGVSYDEYPVVVPKRAALLLDRIMVALQKAVDEENSVKSTKNRIPIEETMD
HalhaASTCC  MCVPQDISLFMRSVVRW-------TLLVTICLIIPPPTAAAPKFTDYKEMGVQYDEYPVVVPKRAAMLLDRIMVALQKAVDED-SAKTVKNRIPIEDTMD
NezviASTCC  --MEKKCLIVMRSVVRW-------ALLVTICLIIPPPTAAAPKFTDYKEMGVQYDEYPVVVPKRAAMLLDRIMVALQKAVDED-TVKTAKNRIPVEDTMD

RhoprASTCC  LQRRGQQKGGRIYWRCYFNAVTCF
HalhaASTCC  LQRRGQQKGGRIYWRCYFNAVTCF
NezviASTCC  LQRRGQQKGGRIYWRCYFNAVTCF
```

Allatotropin

```
RhoprAT  MRWSSLLVLVALASIINCIKAGSPSSALYSSAARASGRTRTIRGFKNVQLSTARGFGKRTYPDSQLQPDLIPADWMAEELSSNPELARFIIRRFIDVDQD
HalhaAT  MRSLPIVILV------LCG--------AVCLARPPQTRPRATRGFKNVALSTARGFGKRDGLQ-LQ-QPRLKAEWLANELTNNPQLAGMFVRRFLDVDSD
NezviAT  ------------------------------------------------VALSTARGFGKRDGLPPLQ-QPRLKAEWLANELTNNPELAGMFVRRFLDVDAD

RhoprAT  GLVSPVELLRNTVCQEPN
HalhaAT  GFISPNELLATPH-----
NezviAT  GFISPNELLARTH-----
```

CCAP

```
RhoprCCAP  MQLLVPCFLLFTALVFAVLTDDVFLQKRVYFPGEIAEPIDPKMKKPFCNAFTGCGKKRS-DESMATLVDLNSEPAVEELSRQILSEAKLWEAIQEARMEL
HalhaCCAP  MLL---YVSILLSVACLAMADDVILQKRMFYPGEATEPFDPKVKKPFCNAFTGCGKKRSGGDSMATLVDLNSEPAVEELSRQILSEAKLWEAIQEARMEL
NezviCCAP  MLL---YVSILLSVVCLAMADDVILQKRMFYPGESTEPFDPKVKKPFCNAFTGCGKKRSGGDSMATLVDLNSEPAVEELSRQILSEAKLWEAIQEARMEL

RhoprCCAP  LNRKQQQSDRIPLQPLPLTTIRKRSHYLYT
HalhaCCAP  NNRKQQEE-QMHPLPLPLTGVHKRSQYLYT
NezviCCAP  NNRKQQEE-QMHPMPLPLTGVHKRSQYLYT
```

Corazonin

```
RhoprCZ  MNFRSSCLLIFIIYSIVHVFGQTFQYSRGWTNGKRAGIPSKEVTACQLQRIKSLLEGKTIPQLYW-PCEWSPFMEAALSRQMKTSELTSLPVVAPL-TPE
HalhaCZ  MWCRLQSLL-LIAILVGSALAQTFQYSRGWTNGKRSFPG--SQTACQIQRLRAMLQGKPISPSYHLLCDLYRLPEEELK----MDQLDKT-LRNPGPEDA
NezviCZ  MWFRLQSLL-LVALLIGSALAQTFQYSRGWTNGKRSFPG--SPTVCQIQRLRAMMQGKPFPPSYHLLCDLYRLPEEDLK----MDQLDKTQLRNPGAEDT

RhoprCZ  IEENCC
HalhaCZ  IEK---
NezviCZ  IEK---
```

Eclosion hormone

```
RhoprEH  --MK--KLLLVILLTSFLA-EISGRQIGVCIRNCAQCKKMFGVYFEGQMCADTCLKYKGKLIPDCEDIASIGPFLNKL-
HalhaEH    MDFSKLLAVMLLSCLCAELVPANQVGVCIRNCAQCKKMFGAYFEGQLCADTCLKFKGKLIPDCEDVASIGPFLNRID
```

IDSLRF peptide

```
RhoprIDLSRF  ------------------MFHNILLVLA-EACHPYEPFKCPGDGACISIQYLCDGAPDCLDGYDEDSRLCTAAKRPPVEETSSFLQSLLASHGPNYLEKL
HalhaIDLSRF  -MDDGTLKMDSPRNSPDEDLEGRNQEMG-EACHPYEPFKCPGDGACISIQYLCDGAPDCLDGYDEDSRLCTAAKRPPVEETSSFLQSLIASHGPNYLEKL
NezviIDLSRF  -SRLYGHVSAKRNS-EACHPYEPFKCPGDGACISIQYLC---DGAP-----------DCLDGYDEDSRLCTAAKRPPVEETSSFLQSLIASHGPNYLEKL

RhoprIDLSRF  FGSKARDALAPLGGVDKVAITLSESQTIEDFGAALHLMRSDLEHLRSVFMAVENGDLGMLKSLGIKDSELGDVKFFLEKLVNTGFLD
HalhaIDLSRF  FGSKARDALAPLGGVEKVAITLSESQTIEDFGAALHLMRSDLEHLRSVFMAVENGDLGMLKSLGIKDSELGDVKFFLEKLVNTGFLD
NezviIDLSRF  FGSKARDALAPLGGVEKVAITLSESQTIEDFGAALHLMRSDLEHLRSVFMAVENGDLGMLKSLGIKDSELGDVKFFLEKLVNTGFLD-
```

ITG-like peptide

```
RhoprITG-like  MLSVILLLTIGVQAAFGWGGLFNRFSPEMLSNLGYGGHGSSYRVQPFLQNNGAMETLQELQEAEMEGPCYGKRCTANEHCCPGSVCVDVDG-MGSCLFPY
NezviITG-like  MLPAVLLLSAMLHPALGWGGLFNRFSPEMLSNLGYGGHGTSLRVQPFLQNPGAMETLQELQEGEMEGPCYGKRCTANEHCCSGYVCIVVDGASGSCMFPY
HalhaITG-like  MLPAVLLLSAVLHPALGWGGLFNRFSPEMLSNLGYGGHGTSLRVQPFLQNPGAMETLQELQEGEMEGPCYGKRCTANEHCCSGYVCIVVDGASGSCMFPY

RhoprITG-like  GLGQGELCRRHSDCDTGLICSDTGDGAKTCQPPFTAPKQYSEECTMSSECDIHRGLCCQFQRRHRQAARKVICSYFKDPMVCIGPVASDQVKDDIERTAG
NezviITG-like  GMGQGELCRRHSDCDTGLVCSDTGEGSKTCQPPFSAPKQYSEDCTMSSECDIHRGLCCQFQRRHRQVARK-MCSYFTDPMVCIGPVASDHAKVEIERTAG
HalhaITG-like  GMGQGELCRRHSDCDTGLVCSDTGEGSKTCQPPFSAPKQYSEDCTMSSECDIHRGLCCQFQRRHRQVARK-MCSYFTDPMVCIGPVASDHAKVEIERTAG

RhoprITG-like  EKRITGKTAAFNHLRR
NezviITG-like  EKRITGKTPVFL----
HalhaITG-like  EKRITGKTPVFL----
```

Long neuropeptide F

```
RhoprLNF  MNCWLLWLWTGLMACNAAMAMAQPIPADAMARPARPKSFASPDDLRTYLDQLGQYYAVAGRPRFGKRAGGINPRLHLAVDGVNYRYPL-ADASDLYDLLF
HalhaLNF  ---------------MVMACQADPLPADAMARPARPKSFASPDDLRTYLNQLGQYYAVAGRPRFGKRTSMHSPRLHIPTDGMNYRYPAVPDASELYDFLY
NezviLNF  MRTWIVCSSI---CLMVMACQADPLPADAMARPARPKSFASPDDLRTYLNQLGQYYAVAGRPRFGKRTSMHSPRLHIPTDGMNYRYPAVPDASELYDFLY

RhoprLNF  QQQSTE
HalhaLNF  QPLAE
NezviLNF  QPLAE
```

SIFamide

```
RhoprSIFamide  MSRTLFVCCFTLVVALIFLDAAMATYKKPPFNGSIFGKRAGPSSDYETAGKALSTMCEIAAEACSAWFPVQDNN--
HalhaSIFamide  MPRALFLSFLVIAFAMLVFDVASASYKKPPFNGSIFGKRAGPPTDYETAGKALSTMCEIASEACAAWFPVQENNLN
NezviSIFamide  MSRVLFLSFLVIAFAMLVFDVASASYKKPPFNGSIFGKRSGPPTDYETAGKALSTMCEIASEACAAWFPVQENNLN
```

Pigment dispersing factor

```
RhoprPDF  -------------------------------------MNSGSVSVKELASWLLYLSQHHDQPHKRNSEIINSLLGIPKVLIDAGR
HalhaPDF  MKMIGVIFGG-LVCLAMFTDVSSLPAYTLNDKMLDKLLLQTLSGKETPIWLSELLRSEDHTHKRNSEIINSLLGIPKVLNDAGR
NezviPDF  MKTMICVIFGALVCLAMFTDVSSLPAYTLNDKMLDKLLLQTLSGKETPIWLSELLRSEDHTHKRNSEIINSLLGIPKVLNDAGR
```

Proctolin

```
RhoprPRL  MATTTQSKVMSREVIVVAVLMMVLLSSSMVQSRYLPTRGADDRILRLRQLLKDLMENDLDPIMEHPAAPNGQYDPRLYKRAAPPVQWDAVGAQFAGN
HalhaPRL  ---------MYKQSL--ACFLALMMLLAFTEARYLPTRSQDDRLLRLRQLLKDLLENDIDPV-EHP---MGGFEPRLYKREAVAYDRPMQYLH----
NezviPRL  ---------MYKQSV--ACLLALMMVLALTEARYLPTRSQDDRLLRLRQLLKDLLENDIDPV-EHP---MSGFEPRLYKREAVAYDRPVQYLH----
```

Ion transport peptide

```
RhoprITPA    MHQERRALAGLVVASTLLSWAVAGPSSRLVLSHPLNKRSFFDLQCKGVYDKSIFARLDRICEDCYNLFREPQLHSLCRSDCFASKYFAGCLEALLLREEE
RhoprITPB    MHQERRALAGLVVASTLLSWAVAGPSSRLVLSHPLNKRSFFDLQCKGVYDKSIFARLDRICEDCYNLFREPQLHSLCRKNCFTTDYFKGCLEVLLLQDEM
HalhaITP     MHQERRALAGLVVASTLLSCLVAVPTSRSIMGHPLNKRSFFDLQCKGVYDKSIFARLDRICEDCYNLFREPQLHSLCRKNCFTTDYFKGCLDVLLLQDEM
NezviITP     MHQERRALAGLVVASTLLSCLVAVPTSRSIMGHPLNKRSFFDLSCKGVYDKSIFARLDRICEDCYNLFREPQLHSLCRKNCFTTDYFKGCLDVLLLQDEM

RhoprITPA    NKFFQMVEFLG------
RhoprITPB    ENIQTWIKQLHGAEPEV
HalhaITP     ENIQTWIKQLHGAEPEV
NezviITP     ENIQTWIKQLHGAEPEV
```

Bursicon alfa

```
RhoprBursalfa   ------- --MASALSSSEHKAQQIDECQVTPVIHVLQYPGCVPKPIPSFACTGRCSSYIQPFKFGIYSCILKLLLSVRLEDLADGEIVHVLSGEWRARG
HalhaBursalfa   MRIFAFIAQLLVVAYCQSDDSKRPADECQVTPVIHVLQYPGCVPKPIPSFACTGRCSSYIQVSGSKIWQMERS-------------------------C
NezviBursalfa   MRIFAFLAQLLVIAYCQNDGTKRPADECQVTPVIHVLQYPGCVPKPIPSFACTGRCSSYIQVSGSKIWQMERS-------------------------C

RhoprBursalfa   QCCLVLSESQAWRKKVQKGMKVNLCI--KRNVCACLQVTTKAPLECMCRPCTSVEESAIIPQEIAAYADEGPLNNHFLKPQ
HalhaBursalfa   MCCQESGEREAS--------VTLFCPKAKPGEKKFRKVMTKAPLECMCRPCTSVEESAVIPQEIAGLTDEGPLNQHFMKPQ
NezviBursalfa   MCCQESGEREAS--------VTLFCPKAKPGEKKFRKVMTKAPLECMCRPCTSVEESAVIPQEIAGLTDEGPLNQHFVKPQ
```

Bursicon beta

```
RhoprBursbeta   -------------------------MPSEIHLVK-EEFDELGRLHRTCSGDIAVNKCEGACSSQVQPSVITPTGFLKECYCCRESYLRERLITLTHCYD
Halhabursbeta   MEWYNAALILLVVVSAAAESEEACETLPSEIH--TKEEFDELGRLQRTCSGDIAVNKCEGACNSQVQPSVITPTGFLKECYCCRESFLRERTVRLEHCYD
Nezvibursbeta   MEWYNAALILLAVASATADNDEACETLPSEIH--TKEEFDDLGRLQRTCSGDIAVNKCEGACNSQVQPSVITPTGFLKECYCCRESFLRERTVRLEHCYD

RhoprBursbeta   PDGMRLTQNGHSTMEIKLKEPSDCKCFKCGDYTR
Halhabursbeta   PDGVRLSHN-MGVMDVRLREPADCKCYKCSDYTR
Nezvibursbeta   PDGVRLTSN-VGVMDVRLREPADCKCYKCSDYTR
```

GPA2

```
RhoprGPA2    MSMVKILCMFLILTCLDNVFCKDAWGKPGCHKV--GHTRKVSIPDCVEFHVNTNACRGYCESWSVPSPLETVLHNPRQAVTSIGQCCNIMDTEDVEVSVL
HalhaGPA2    MVNKFLWCMLLVSSFPGLLSKQDAWGKPGCHRVGIGHTRIVRIPDCVEFNVTTNACRGYCESWAVPSPKETLVSNPRQAITSVGQCCNIMETEDVEVRVL
NezviGPA2    MVNKFLCCMLLLSSFPGLLSKQDAWGKPGCHRV--GHTRKVRIPDCVEFEVTTNACRGYCESWAVPSPKETLISNPRQAITSVGQCCNIMETEDVEVRVL

RhoprGPA2    CLDGTKDLVFKSAKSCSCYHCKKD
HalhaGPA2    CLDGLRDLVFKSAKVCSCYHCKKD
NezviGPA2    CLDGLKDLVFKSAKSCSCYHCKKD
```

GPB5

```
RhoprGPB5    LDPSSTLDCHRRIYSYKVTQADSEGRICWDTINVMSCWGRCDSNEVNFQISDWRFPYKRSYHPVCIHGVRELRRVVLKHCEEGAEPGTEQYDYQHATTCR
HalhaGPB5    VDPSSTLDCHRRVYNHKVSKADSQGRLCWDTISVMSCWGRCDSNEI--QISDWRFPYKRSHHPVCLHGAREQRTVVLQHCEEGAEPGTEIHQYLQAVSCH

RhoprGPB5    CHVCRSSEASCQGLR
HalhaGPB5    CHVCRSS-ASCQGLR
```

Supplementary Information 6:

GPCRs transcript sequences identified for *Nezara viridula.*

1. Neuropeptide receptors Family A

CNMamide receptor

>Nv_A1
AATTATTGTTCCCTCCATATGTCTATACAAATATATAAAACAAAATGAAGTAAAACAGAAAGTGTG
AATATATGTCCTTTGGATAAATTGTCACATGCACTTTGTCGAGGGGTATATCATTTTAATTTTTCA
AGAGTTTATTTATTTATTTTTCACCGGAATACGAGTAAAAAGAAGGAAAGGGGAGGAGGAGCGAGC
GGTGGTGGTGATCCAGCAATATGCACAATTGCTCTTCTACATCAACTTCGGAATCAACTTCGTCCT
CTACTGCGTCAGCGGGCAGAACTTCAGGAGGGCATTGATATCGCTCTTCTGCCCGGACATCAGGAG
GAGGGCTGAGACCACCCAAGTAACAACAGTTGTGTCAGAGTACACGAGAAGCGCCAGCACAAGAAG
GACAGTGACAGTGAACGGCAATTGGAGAGAAGCACACGAGATGGTACCCATCAACAGACCCCATCT
CCACTAGAGAAATAGCTGTCTGCCATCTAGCGCATAGAGGGCCTCGTGTGCAAAGACACTAAACGG
ACACGTGTTTTCCACAAACTTTTAAGAGTAATGCTTTCTTTCTGGTACTAGAAGAGGAAAAAAAAA
ATCAAAGATGACTAAATCCAATGACAAATAAATGTTGCATGTTGGTAAATTGTTGTCTTATTCACT
TCAATCGGGGTTCGACAATATTGACACGCATTTATCAACATGGCAGCTCATCATAAAACTCCAAGT
GAATTGAAGATTTTGAGGTCCAAGAAAATTTAAATAGGTAAGTAAATATTAGGACGAACAAATTTG
ATGGCCAATCGGATGTGAAGAATACAATAAGAAGTTAGGACCGGCCTTATAAATAAACATCTCTAA
GAAAGTTCAATAGGAACAAAATACTGATGGTTACCTTTAAAAGTATATCTTTATAGCCTTATTTAT
GGACCTTTATAAGGGAGGTTAGAACATCTCATATAAAAGGTTTCATTTAATTTCACGCATTTAGCT
TCATTTAACAGTATTTCTGGTTAATAAATTTACCTATTATATTTTTCATAGTTAAGATACCTTTAG
ATCAGTAGCAGATTCAGGATTTAATTTTGGAGGAACTTCTTTTTAAATACTTTTGAAATAATAATA
AAAAATCTACCAACATAAAATCGGTAATTACGGGAAAATAATGCTTTTAGAGATTCTTGACACTTG
TTTCCATTTAGTAGCCCTATATGCATAATAATTTAAAAATAAAATGTATTGTGATTGAAGTATTTT
GTACTGTTCTCTATGGTATTCCTTGACGATTAAACCGTACATGTAGAAGAGATATGCATGGAGAAA
GACGTACTTACTTTTCGGATAGAAATGGAAAAAGGTAAGATTGTGAAGCATAGAAAGAAAACATTT
AATTTAAGACACTTATGCGTTCCAACTGTAAACGAACATATACGGACCGAATACAAACGATTCATC
CTGATAACGAATGGAAAATAGACATAAACTTGATATATGAGGGATATTCTATTAAAAATGTATAGC
GACAAAGGAAAAATATACGTTAAATATTAAACAAATAAAAATATAATACAAAGTATGAGTAACGCA
TCTGAAGTAGCTTATATCAGTTATTCTCACAGTGCTTGAATAATAATGAAATTGATAAATAAAAAT
CAAAGAGTAGATAAACTAAGAGCTGTCGAATCTATGAGAACATCCATAGTAGAAACAAAAACATAG
TAGAAGATTTGAATACTGGCGTATTCAGACAAACGTAGATTTGAAAACACGAATCATATTAAACGC
TGTACGAAATCAGAACTTTCTTCTTTGCAATTTATACATTGCAGAAGTCTAAATCACCTTTCTCAA
ATAATTGACAGGACTAAGAAGTTCACAATTGAAACTGAAAGTTCTAAAAATTAGTTAATACTATTG
ATATTTTAAAAATATATTTATTTTACTTCTCCTCCATCATTTCTTTAAAAGAAGGAAGGCATTACT
CCAAAACCGCCAAAATGTTCACTAAGAACGACATATAAGCATTACCT

Myosupressin receptor

>Nv_A2
TGGTAGCAATCGGTGAAGAACTCCTGGCCACGATAAATGGACAAGAGGACGAGGACGCCCTGGGGA
AACTCCGTGATCAAGAAGAGGAGAAGGACTGCTAGGAGCATCCTCGTCGTCCTGTCGGTTTGCCGC
TCCTTCTCGGCTATCCTAGGAGTCTTTCGGCTACCGCTGGTCAGAGCTTCCCGTCGTCTCTTGGTG
TCGATCAAGGCCGAGATGAGCCTGAGGCTGAGTATGGTCAGAGCGATGCAGGGTATGATTTTGATC
ACTATGCTGTACACCCAGAAATTTATGGTGGCCAGTAGGTTGTCGTTAGCTCTGCCTAGCTCACTG
AGGTCCACGTAGTATAAGGTAGTATTTGTGAACTTTGTCGTATTGACTGTCGGTTTGTTGCCGAAT
TCGTCCAAAATGGTTCCCCTCGGCTGGATATCGAAGGCTAAGTAGAGCGGTATGCAAAGGATAGGG
CAGATCAAGTAGCCACTGGTTATGGCAATTATAGTCTGCTGCATACCGCACCACTCTCTGTTCCGC

TGGGGATAGACGACAGCTATGTAGCGCCACACGGCCAGCGTTATGGTCAGCCAGATGGATATCGTG
TGGAAGACTTGAGAGAAGTGCGCATGCAG

Proctolin receptor
>Nv_A3
AGGTCGGTAAGAGCTGTTTATATGACCCGACTTCCTACCTCCCTATCCAACTTGTCGCATCGTCTA
AACGGCCTTGAAGAATAACTACAGTGTGGTAAATTACAGATTAGGGGAGTTGTGATTTCTTCATTT
TCTAATTATTGCAAAAAACATTATTATTATTGAAACTTTATTACAAAATTTACAAATAAATACTGT
TTTTAAATAGCATTTACATATATTTATTCAACTAACTTATAAAATGTCCTTTATATACAAATAACT
ACAATGAATCGATTTATTAAATTATTAAATATAAAGTAGACAATAATTACATTTTTTAGTATATGC
TATCTGTGCATTGAAGAACGATAGCTTGCATTGGATGAAAATGTATTTGCTCTCTGATGATTAATG
GCCAAGGATGGGCAAAAAGTCCTATACATCGTTTGTCTATATTTATCACTCATAGCACAGTATAAT
AAAAAATTAAAAGCTGCATTAACAGCAAGTAGTAAATTAAATATGTTTCCGACGCCATGCACTATA
TTGTAACCATCTGTACCTTCTTCTGGATTGTAAAATATAGATAATATTAATGTAATAGCAGAAGGT
CCTTGACAAAATAGAAATAAGAATACGACTGCTACCAGAGCTGTTGTAATTTTGTTCTCTTGTTTT
TCTTTATAATTCTTCCTTGAATTTCCCTCTGTTAAGTTCTTTCTTTTCTGTTGACTTATTCTTACT
GCATTTATAAGTAAATAGTTTAATGTTCCCAGAAGTATCAATGGAATAAACACAAATGTTATACCT
GAGAACCAGTAAAATATTGTTTTATAAGTTTTATTTTTTCCCAAAGATGAGTACTGGATAACAGTT
GTTTTATTTGTTTCTGGATTTATGTTTATTTGTGTATTCCATTCAAATGGAGTAGTAACAGTTGAT
ATTAAGCAAAACATTACTACAAAACCGATAACTTTTCTTGCTCTTGCTTCTGTACAAACAACTTTT
CCTTTTAACGGATGGCATACTGCTATATATCTCTCCAATGTAAATGATACAGTCAGCCAAGTTGAT
ATAGCAACTGTAGCATCTGCAAACCACACTGCAAACCTCCAATATTTCCAATATAATATATACTTT
GTATCACTCATGTGATGTTCTAAGCTCAGTGTCATATAAAATATTAAATACAATAGATCTGATACA
GCGAGTGCTGTTAGGTATGTATTTGTTGAACCAGACATTCTCTTTCTAGTTAAAACAATTATAGTC
ATGACATTTCCGAACAAACCAATGATGAAAATAATTGGTGCTACCACATTTGGAATCCAGAAGCGG
CATTGTTGTAAAAAATATAATGATTTGGACTTTGGGTTAACTAAAGTGTTTTCTGTGCCATTCATT
TCAATAAGAATATTTTTTCCTGGTGTAACATGACTACAAATTTCTATTTATAGAGGTTTATAGTTA
AATAAGTATTTTCAATGAAGATGATGATGTCCTTTTTCTTCATCTGAAAATTTTTCATTTTAATTT
TGTAACTATATTACTTGAATTAACATATTTGTTTTCTATATTTGTCTTTTCTACATTGCTCATCAA
TTCATGTCTAACTAGACTTTAATGTTAAACCACAATCACAACAGTCAATTCAAAATATTATGTATA
GTATGTTTTGTTGTTTATAATGTCAGCACTAAGCACTATTATAAGTATACTGGGCTGTCTTTCAGA
CCTCCGGGCTGTAATTAGA

Myoinhibiting peptide precursor
>Nv_A4
GTGGATTTTTAATTAGGTCGAGGATTACAGAGTTCTTCTGATTTCTTCCTCAAGATTATTTAAACC
AGGTTCGAAGGATTCGGAGTGGGGGAGACTGCGTTTGCCCCACAATCCTTTGAGATTTTGCCAAGC
CGGATCACGTTTTCCCCATGAACCTCGGAAACTCCCCCAGTCTGCTGCTCTCTTTCCCCAACCGCC
GTGAAGGGAATCCCAACTTTTCTTCTCCATTTCTTTGTCTTCATCATCTTCATGTTTAGATCTCTT
TCCCCAGCCTGAAGATTGGAGGTCAGACCATGACCGCTTACCCCATCCACTGGTTTGCAAGTCAGA
CCAAGCTTTTTTTCCCCATCCAGTAGATTGCAGATCAGACCATCCTCGCTTTCCCCATCCAGCAGA
TTGTAGGTCAGACCATCCCCGCTTGCCCCAGCCGGTAGATTGGAGGTCGGACCATGCTCTCTTTCC
CCATCCGGAAGAATGAAGATTGCTCCAGGCTCGCTTTTTGTTATCATTCTTGTCCCAATCAGCGGT
TTGGTATTCATCAAAATATCGTTTGCCCCAACCTGTTTGAAGATCCCCCCAGGCTCGTTTGCCCCA
GGATTTTGAGAAATCTTTCCAGGCTCTCTTCTCATTTCCTATCATTATTGTGTCTTCAAGTTGTTG
CATTGGTGAAATAGGATCCTCTTGCGATTCTTCTCCAAGTGTTAAGGCAGCTAATCCCACTATGAA
GATTAAATAAAGTAACTTCATTTTATTGTATATGGGCTGGGTGTGCCCAAGGATGCTTTCACGATC
CCAGGGTCGATCCATGGCTAACGTAACAGAATAAATATTGATCTGCCGGACTCGAACCTTGCACCA
GAGTTTGCGAAGGTGATTCCG

>Nv_A5

TGTCATCTTGTATCAGTTTCTATGTGCCCTGTATTGTGATGTTGGGTATTTATGCACGCCTATACT
GTTATGCTCAGAAACATGTTAAAAGCATTAAAGCTGTTACAAGACCATTGACCTCACCCTCTATTC
CAGCAGTTCATCCTTCTTCCTCTCCTTACCATGTGTCTGATCACAAAGCTGCCATTACAGTCGGTG
TTATTATGGGTACCTTCTTACTATGCTGGGTTCCTTTCTTTTGTGTAAATATTGTAGCAGCATTTT
GCAAAACATGCATTCCTGGAATGGCCTTCAAGATCCTCACATGGCTGGGATATTCCAATTCAGCAT
TCAATCCAATTATTTACTCTATATTCAATACTGAATTCCGTGAAGCTTTTAGAAGAATTCTCACTG
CACATTACCCTCCATGTTGTTGCAAAGGTTATTCATCAGTCACCATAACTCTTGGCGAAACAAAAC
CCAGAATCGGAGATCAGATGAAACAAAATGGGCGCAGAGATTTTTCTCCCCGATCGTCAGTAGGCT
CCTTACGTCAAATAAGAGTAAACTCAACAGATAAGGTAATG

>Nv_A6
AGCAACTCCACACCCTGCCAGCTAACTTCGCAGCAGGGTTATGTAATATATTCGTCCTTAGGCTCC
TTTTATATTCCTCTCTTAACAATGACTATTGTTTACATAGAAATATTTATAGCCACCAGGAGAAGG
CTACGCGAACGGGCGAGGGCGTCTAAACTCAATGCTGTAAAACAGAACCTACAACAGAACAATTCT
ATGAGGGAGAAACATTCTCCAGTCGATGGTGAATCGGTG

Corazonin receptor
>Nv_A7
CTCCATGATCCGGGAATACCGGGGAAGTGGTCTGGCAGGTCAGTCTGTGCCGAACAGCCGGGAGTC
GAGGAGTGTCCGATCTGTGTCCTCTAGTGCCAAGGATAACAGGAGTCTCTGGAGTAATGAAGAGGA
AATCTGGGTGGGATAGAGGAATACTATAGGAAGTAAAGGAGGCTGTGACAAAATTATTTGGAGGTT
GAAAACATCCTCCGCTTGAATTAAAGAACATATCAGCCATTGCAACGTTGGAATGAACGAAAGGTG
ATGGGTAAGGACACCTACCTGCGGGACCTGGGGGCCGGCAGGTACGCCCTCCCGCTGGAGCTGTGC
GAGCGGTTCAACGAGAGCGGAGTGAACCTGACCAGGATCGAGTGCCTGGAGCACGCTCCGACCCTC
ACCTCGGAGGCCCTCACCAGGGCCATCGTCTTGGCCGTCATGGCCATCATCTCCCTCGTCGGCAAC
CTCCTCACCATCTTCAGCATCGCGGGCACCAGGGCGGGCCGGCGCCGCAAGAACCAGACCTGGTCC
GCTGTCTACGCCCTCATCCTCCACCTCTCCATCTCCGATCTGTTCGTCACAGTTTTCTGCATTGCT
GGAGAAGCCCTGTGGTCGTACACCGTCCAATGGCGCGCAGGTAACGTGGCCTGCAAGGTCTTCAAG
TTCTTGGAGATGTTTTCCCTCTACCTCTCCACGTTCATCCTCGTCCTCATCGGATTGGACAGGTTC
ATCGCCGTGCGGTACCCGATCCGTGCCATCAGCACAGCCAAGAGGTGTTCCAGGTTCGTCGCCATT
GCCTGGGTCCTCAGTATTATCCTCAGCATCCCGCAGCTTGTCATATTTCATGAAGGCAAAGGTCCA
TTTTTCGAAGACTTTTACCAATGTGTGACGTACGGTTTCTACACAGAACCTTGGCAAGAGCAGCTT
TACACGACTTTTAGTTTCGTTTGCATGTTTATGCTACCGTTATTCATTCTCATCACATCTTATGTA
TCAACAATAGTAACGATATCAAAAAGTGACAAAATCTTTCAAAACGAAAGCATCAACTCTGTGAGA
AAATAT

Adipokinetic hormone receptor
>Nv_A8
AAATGATTAAGTGTTAGTTCAAGAAACACTCTTCTAGCCGTTCAGTATTTCATCATACGTGGGCGG
ATTTATGATTTTCTGCTCAACACGTCCATCAAGAAGAAGCAGCCACCCTAGAACTAAAATATGGGA
AATGGTCCAGATTACAGGCGTGTGTGGTTTCCAGACTGGCCAGTGCATGTCACAGAAGACAACAAA
ACATTCCATATACCAATCGATATGCGTTTCAACGAAGGGCATAAGGTGTCATTGCTATTTTATTCA
ATTTTAATGGTCATTTCGTCGGTAGGCAATATAAGCGTCCTAGCGATATTATTGAAAAGGATGAAG
CACAGCAGGTCGAGAATCAACATGATGCTGATCCATCTTGCTATAGCAGATCTATTGGTAACGTTC
ATCTTGATGCCAATGGAAATCGTATGGGCAGCCACAGTGTCTTGGTGGTTTGGTGACATTGCATGT
CGAGTTGCTGCTTTTTTCAGAACGTTTGGCTTGTACCAGTCATGTTTTGTCCTAGTTTCCATCGGG
ATCGATAGGTACTATGCAGTACTCAAACCCTTAAAGTTGTCTGCAGCGAATAGAAGAGGAAAATTC
ATAATTACCTGCGCATGGATGGCTTCCGCTTTATGCAGTCTGCCACAGACAATCATTTTTCACGTT
GAGACACATCCTAATGTCACGTGGTTTGAACAGTGCATAACAATTAATTCTTTTTCATCATATGCA
CAAGAATTCGCCTATTCATTCTTTGGAATGTTATCGATGTACTTGCTGCCGCTGATATTTATAACC
TATTTCTACGGCTCAATATTCATCGAGATATGTAAAAGATCAAAAGATCATCATGTTAACTCAGAC
AAACTCAGGAGGAATAACTTAGCTTTCTTTGGAAAAGCCAAAACCAGAACGCTTAAAATGACCATT

ACAATAATATCTGCTTTTTTTATATGCTGGACACCATACTATATTATGGCTTTTTGGTACTGGGTA
GACAGAGATTCTGCTTCAGAAGTGGATCAACGTGTTCAAAAGGCACTATTCTTGTTTGCTTGCACG
AATTCGAGTATTAACCCCATTGTATATGGAGCTTTCAATATCCGTAAACGTAAGAATGTTTCAAGA
AGAACAAGAGAGAGTAACACATGTTCAACAGAAATGAAACTCCAACAAACAGGACAGAAAAGCCCA
ATTCAATGAGTGAACCTAGTCTTGATTTGATGCTCTATTTTATAACAACACCGCTGAAAAACAGAA
TAATGATGAATTTCTTCATTACATTAAAAACTATTTGAACTATCTGAAGGAACCTGAATAATGGAA
ATTCTTTTATTTATTCTTTCATTCCATCCTTACAGAGATAATATATTAGTGATGATGTGGTAATTG
AAACCGGAAAAACAATATCAGAAAATAAATTATGTTTACCTAGAGGTTTGGAGAGCTGAGAAGATA
TCACAAATATTTGTTCCAAATATTCTGCTATAATTTTCAGGGTAAAAGTAAAAAATAAATATATAC
ATAAATATGTATATAAGTATCATGATTATTCTGTAACTATTCATGAATTAGAGGGGCTATAAATAT
TAACCTCATTAAAATAATGTATTTTGTAAAGTTTGAATAATTAAAAATAATTCATACCATAGCAAC
CATCAGAAATGTAAAAATAGACCATGTGAGTAATTTTGATGTTATCGGATTTTAATCATTTGCTTG
AAATGTTGATAACTGACATGTTATCATGCATATGATGACATGAACTTAGGTAGCGTAACAAGAATT
TGATAAATTTTTATTTTATTTTTTGCAAAGATATTTTTATAAAAATTAGAAATCCTGATAGTTTCT
TTTTTTTTTTATTATATTTTATTGTAAGTTAGCAGTGCTGGCCTTAGGGCCAGGCAACCAGGGCTA
TTGCCCAGGAGTGCCAAGCTAGAGCTAAGGGCGCCAGCCATTTGTTATGAATAAAGAAAAAAAGTA
AAACGTATGAGAATAATTTATTTTAGCCTATTAATAAAGCGACTGAATTAGAGACATATTGAATTA
AATAAAATTGAAATGAAATAGATTACGAATTAATCTTGATTGATGTATTATATTTTTTGGTGGGAG
ATGATGACATTGCCCGAAGGCATAAAATGTCTAAGGCCGGCATTGAGAGTTAGTCAAAGTATGCTC
TTACAGATTCAATAACCTTTGCCTGTCTGTAACACAAAATAAAAAAAAATTCCAGTGAAATAATAA
TACAGCTAAAAGTGGAGTTCAATAATTGATATTATTATCATTAATATCTGGGCTATCTAGAGATTC
TGGCAGAAATTTTAGAAAATTTTAGAAAAAAATTGAATTTCAATTAAAAAATATTTGATTCATTCC
AACGAAACCTGAAGTTAATAAAATAAAAATAATGCTT

Crustacean cardioactive peptide receptor

>Nv_A9
TGAGTTCTAGGGATGTAGCCGTATACTTGAAGAAGGTCAAAGACAATATATGGGCTCCAGCAGAGG
ACAAAAACTAAAACAATGATAAATGTCATCTTAACTGTTTTCACTTTTGCCTGAGGTATAATACCC
CTGGAACTTGCTCTCCTTGTATCTTCACCGGTTGCTTTGTGGGTATACTGCTTGCCCTTTGCCCAG
ATTGTGAATACAATAAAGGCATAACAGATCGATATGATAACAGCCGGAAGCACAAACACA

>Nv_A10
CGGCTTCAGTCCGACCCCAAAGCCCGAGCTAGAGGTATGGCGAGCTTTTGATCCTCAAGCCCGTAA
AATACGCAATCCCTATGATGTTCATATAAGTATAATTATCAACTACAGTGTAACATCAGGATAGAA
TGGAGAGCCTTGGGAAGCAAGTTTAAGTCGTCAAGAATAGAAGAGCCGGTCAAGGATGGGCGAGGA
GGAAATAGCATTGCAAGTGGCCCTAAATGAAACAACAGAAATCAATACATATGACTTCTATGCCAC
CGAGCAGCTGGCTGTACTTTGGGTCTTGTTTCTCCTCATTGTTCTGGGAAACTCCTCCGTACTTCT
GGCTCTGGCTTTCAACAAGAATAGGAAGTCACGTATGAACTACTTCATCATGCAGCTAGCAATTGC
AGATTTAGCAGTTGGATTGATAAGCGTCTTAACAGACATCGTCTGGAGGGTCACGATAGCCTGGCA
CGCTGGAAACGTAGCCTGCAAAGTTATAAGATATTTACAGGTATTGGTTACATATGGATCAACCTA
TGTCCTAGTGGCCCTTAGCATCGACAGATATGATGCAATAAAACATCCGATGAAATTCTCAGGAAG
TTGGAGAAGAGCCAAACTTCTAGTTGTTATCGCATGGGCCGTCAGTGCTATATTCTCTTTACCCAT
CTTGGTTCTCTATGAAGAAAAGCTTGTTCAAGGTCAGCTGCAATGCTGGATCGAGCTGCCATACCA
GTGGCAATGGCAACTCTACATGACTGTCGTCTCGGTAGCCCTATTCTTCATACCCGCAATAATAAT
TACAGCCTGCTACACAGTCATAGTGTCTACCATCTGGAGGAAAGGGAGTACTATCATAGTCATCAA
GAGGTCATACAAAGGTATTTCCACTGACAAGGTGAGACTCGAACATGATCATGAATCTAGACGCGC
AAGTTCCAGAGGCCTCATACCCAAAGCCAAAGTCAAGACGGTCAAGATGACCTTCGTCATAGTTTT
CGTATTCATTGTTTGCTGGTCTCCGTACATAGTATTCGACCTCCTTCAAGTGTATGGCTACGTCCC
AAAGACCCAGACTAACATTGCAGTCGCTACCTTCATCCAAAGCCTTGCACCTCTCAACTCTGCGGC
TAATCCCCTCATCTACTGTCTCTTCTCGACGAGGTTATGCAGGACTCTAAGACGGGTCCCCCCATG
CTCCTGGCTCAACGACCTATTTTCCCAGTGCTGCCCTGGACTAGCCTCAGCAACCTGCCTGACGAT
GGATCACAGCTCTACGCTAACATCTTCCCTTCAGTCGTCCAGGGGTTCGAGGCTGTCTTCTAAGCA

CGTCACTGTACTCTAAAGCATAGGAGATAAAAGAGGAATGAACCTGTTGAGGAACAGAAATGGAAC
TGTGCAACCTAAGACATTCCAAAACTACAAGGAGTGCATTTTCTTTCGCACCATCTGTTGTTATAT
GTGTAGTGATATTTATGTGTACATTAATAAACATGACAAATATACTATTAAG

CCHamide receptor
>Nv_A11
ACAGTGAAAATACGAGAAGTTGGAACAGAAACCACTTTAAAAAAAAAATTTTAATAACATTTAGTG
ATTTCTTAACCTATCAACAGCTCTTGATGACTTTTGCATTTAAATGCGGTCGGGACTAACTATTTT
TCCCTATATATATAATTTTTTTTTATATTTTGTTTCATATAATTTTTATACAAAATTGAACAGGTA
GAATAAAATGTAATGTGCATTTCATAAAGAGAAGAAATTGCTTTAATTGTAAATATAATACAACTG
TACAATATGTTTAGAAAGCGATTTTATAAAAACTTTACACTATCAATATATATTTCTTAAAATTTA
TTGAGTGGATTACATTGATTTTCGATATGAGAGAAAGTTTGAATATTTTCCAAAAAATATTTTCAT
TTCGACCTCGTACTTATAAGATATAGTTTTATAGTAATTATTAACAGTGAGTCATTATAATTTTAT
AGTTAATTATAAATAAGCATGCTAATAAATAATACCATACAAAACAAAATGATATATTTTTCAAAT
ATTTACATTAGTTTAATTTTAGAATAGAATTTCAGTACACATTCTTATTAACAATTTACAGATTTT
ATCCAACTCAATACCGAATACGGACAAACGAGATATGACAAGATATTTTATTAGTAAGATTTCTAA
AAGTTTTTATGTGTGTTATTTATTTGTATGATATTTGATTTTTAGTCATAAGATATATAGATATTG
ATTATTATGGAATGATGAAATCCAAATTTTATGACACCAATGAAACGGAACATTCAAGCAACTAAA
GTCAAAGATTTCAACAATGATTCTATTGTTGATAAAATTGTTTTCATGTCTCATTTGTCTTTCTCA
GATAGTACTCCTGATTTATCAACAATATGTTACTCTACAACTACAACATATACCATTTCTAATTGG
TGTTAATGATTTATCTACAAGGAAAAACTATTAAAATTTATTAATCAAGTGAAATAGTTATTGAAT
TTAAGTTTTTAAAAATCTAAAATTAAACTTCAATTTTGAAGAAGAGCAAACCAGATTAAACATTTC
ATTCAACAATTTTTGGACCAAAATATTGAAATTTTAACTCTTTTAATGGTAGTTTTAAAATAACAG
AATTACTTACCGCCTTTGATTCAAATAATTAAACATAGTCTTAAATGATTCTAAAATAAAAGCCTT
ATAGTTAATAAGTAAAGAGTTTTGTTTTGACAATTGAAGCATTGTAATTACATAATCATTATCTTT
AATCAGGAGTGGATTGTATGGCATAGAAAGGGACTGTATGGCACAAATCGTACCTTCAAAATTTTA
GAGGAGAGATACAATTTTTAAGAGATTTGAGGAGAAACATTTTGACAGGGTTAGGAATTTTTGGAT
GTCCATATGTCAAGAATGGCTTGCCTAGAGATCCCAAATTCTTCAGAAAAACACATGGGATAAGGA
GGCTTGGAAGATCGAAGATATGATGGTAAGAGTGGCCCATGATTCCATAAGAATGAACTGTGAGGA
ACTGGAAGGCAGCGGCACACAACCGAGACGACTGGTCTTGATTGATTTTGGAGACCATGACTCATT
AGTGGGTTGTATTGCTTAGGAAGTGAATAAGGAAAGAATTTTTGCAGGGAATTATATATGGAGTTT
CAATTTGAGTGAAACTTAAAAACCAGGGAATAAGGGGAAAAGACTAACAATTTATGATTTATCATT
TTTCTAATTTATATTCCCTTGCACTTTTTTTAAGAAGAGTAATGAAACTTCTTGGAATTGGGGTGG
GGTGTGCCATCAACCTTGAAGAGATGAGCGTCTCTGTTTTAAATAATATTGAATACAAGTGAATAC
TTGTGATTACAATACTTGAAATAATGTTTGTTTGGTGATGTGAGATGGGCTATGTTATTTAATCTA
ACTGAGGCTTTTATTTTTTAATCTTCCAAATATGAGCTGATTTCAAGTTTTAAATTACAGAATTTG
AATGAAAATACAAATTAATACTATAATAAGCTGTTGCAGGTGACTCATAAAATTCATGATGTCGTA
ATATTAAAGATATTAACAAGGACAGCACTTAGAGTGCTGTGGTGTTCAGGAGCTAAGACGGCTTCC
TGGTGCAGCAGAGTGGATGTCAAATCTTTTCAGCATTGCTCAGAGTGTAGTGACTTGTAATAACAG
AGTTCTGCCGATGGTGCTTCATGGAACTGCTTTTGTGTGTCATGGTGACATCGGTGCAAGGCGTAT
CGGAACGCTTACAGCACAACAGGTACCTGTTGAAGTACCTTCGGAATGCTTTACTGACACAGTAGA
GAGCGATAGGATTTATGCAAGAATTAATGAAACTCAAGCAGAAACCGACGATCCTGTATCCATGCC
AGAATTCATTGTAATCCTCTTCAGCCGTAGGATAGAAGTGGAACCATAACATGAAAGTGTGATATG
GAAAAAAGCATACGAAAAAAATTATAACGAAAGCGAGAACCATCTTCGCCACTTTCTTTCTAGCGT
TTATCTGGTTCGACTGGCCTTGCATTTCACCAGGCATATTCTTCGTACTTAGGACAAGGTGTCTAG
CCATTAATATGTAGAAACCTGCAATGATGCAAAGAGGGCAAGCGTAATATGCGAGAAATTTGAACA
AAACTATGGACTGTTTGTAGGGTGGTCCATATTGTGGAGGGAATGGAGAACAGTACTCGATAGTAT
GGTTGTCTGCGACTATGGCGATCTGGACGTTAGAGAAGATGGCGGCCGGCAGGGCTAGAATGAAGG
ATACGACCCAGATGACGAACGCCATTACGATGGTCAACGGCTTGGTGGATATGTGCCTGCGGATGG
GGTTGACGATGGCGCAGTAGCGCTCAGCGCTGAGAGCCGTCAATGTGAAGACGGAGACCCCAATGG
AAATGTCCTTGGTGGCCTCCGAGAGCTTGCAGACGAAGTCGCCATAGGGCCAGGACTCGATGGTGT
AGAGCGTGGAGGTGAAGGGGAGACTGGTCACTATGACCAAGAGGTCACCCAAGGCCAGGCTCATGA
TGTAGGTATTGGGGATGTTGCGCATCTTGGTGTGCCTCACGAAGATGACCACCAGCGTGCCGTTGC

CGAGGACCCCGACCAGGAAGATCACCGCGAAGAGGACGGGCACGAGGTAGGTCTCCAGCCGCTCTG
AGTAGGGCACGTACTCTCCGCACTCCGTGCAGTTGTCCATCGTGAAGCGTATCTCCTCTCTAGTAA
CCTCCGTAATTTTCTTGATTAGTACATGATTAATAGTCCCCAATGTTCAACTTTCTTCTTCGATAC
CATGTTTCATTATGCTCAGGTGAGTAATCCATTTTATTAGTATTAACTGAAGATTAGGTAGGTGCC
CTTCAATCACAGGTCTAAATCAATCTATTTTCTGCGTATCGACCTTTCCGGTATTCTCGCGAAAAC
AAATCGTTGGCCTTCCTCTTTGGAATTCTCGATAAGACGAATCGTTGACTATTTCCGTACGGGAAT
AATATAGTGAGAAGGAGTACTAATCATTCCTTCCGGAAACGTAAACCGAAGCAAAT

Ecdysis triggerring hormone receptor
>Nv_A12
GGGAAACAATGACCCAAAATGTAAATACTCGAAATGGAAGCAGGCATAGAAAGAAGGAAAGCACAA
CAGTGGCGAGCATAAGAACAACCTGCTTCCTAGCACGTATGTTATCTCCACTATTAGCGGTTCCAG
GGTCACGGATAAGATGCCTTGCTATAACTGTGTATAGAACTATCAGAATAGATAATGGTAGGATGA
AGAAGACAGTCAGTATGCTGTAGAAGAAGAATTTCCGCCAGAAGCTATCAGGATCTGCATAACAAC
CAGCCTTACCATTATTCTTCTCAGGTACAGAGTATTCAGTAATATGAGCGATTGGACTGGTGAACA
GAGCTGCGAAAGCCCATGCAAGTAAACAAATCAACATGGCTCTTGCTTTAGTACAAACATAGCCTG
CTCTCAATGGTTGGCAAATGGCATAGTACCTCTCAAAGCTTATGGCAAGTATCGTTAGTACGGATG
CATGGGCTACTGTCAACTCGACGAACGGCACAGCTTTACCTATATGTGGTGCTCAATATTTCATGT
TACATAATGTTTGAAGGAAAGAAACTTTTTTAGACTGTTAGTATGTATCGCAGATGTGATG

Capability receptor
>Nv_A13
GGTGGAATGGAAGGGCGGAGGCGAGCGAAGATAATACGGTGACATGCGCGCATCCCAGAGGCAGCT
TCAGTCCCGCTCAACACTCGCGCCAGAATCTGTCACTTCTTCGCCCGCTCAGAGGTTTCGATTTCC
GCTATGGTCATTTCTGTTACCCAAGATGAGAAGTGGTAGAAAGATCAGATCTTGCCGGACGGTGCG
AAAACGGCGGATACGGTAGCCAAAATGGTGAGGAACGAGAGCGATATTCTGGAGCAGCTGATACTC
TTTTTGAACAGAACTAAGGAGAACTGGACATTGGACGACTACATCGAACTGACGCGAGGATCTAAA
TACCTTCCCATCGCCATTGTACTCCCAATAACTATTATATATGTCATAATCTTCATCACTGGTATC
ATCGGCAACACGCTTGTCTGCTGGGTCATCATTAAGCACAAGATGATGCATTCCGCTACCAATTAC
TATCTCTTCAGCCTTGCAGTTTCAGATCTCATGCTTCTTATCCTTGGCCTTCCTAACGATTTAAGT
GTCTACTGGCAGCAGTACCCTTGGCCGTTCGGAGAGAGCTTGTGCAAAATAAGAGCTCTTGCTTCG
GAAATGTCGTCTTACACATCGGTACTGACCATAGTGGCCTTTACGATGGAGCGATATATAGCCATT
TGCCATCCTCTGTACTCGTACACCATGTCCAGCTTGGCAAGAGTCCTCAAGATCATTGTAGCAGCT
TGGCTGGTGTCTCTTATGTGCGCTCTGCCCTTCGCAGTCTTCACCACCGTCAACTATATCGATTAC
CCTCCATACTCGGGAAACATACTCGAGGAAAGTGCTTTTTGTGCTATGTTAGAAGCAAACATACCT
GAATGGTTGCCGATATATGAGCTGAGTACTCTGATGTTTCTTATCATTCCAATAATAATTATAGTG
ATCCTGTATACTAAAATTGCTATAAAACTGAGGGAAAGGAACGACTACTCACTTGGCACTAGGGTG
GGAGGATCAGTGCATTCAAGAAAGAGACATTCGAAGTCGACGAAGCCTATAATTCGGATGCTAGTT
GCCGTTGTGAGTATGTTCTTCATCTGCTGGGCCCCTTTCCACGCTCAAAGACTGATATATCTCTAT
GGCAAATCTTGGCCCAACTACGTCACACTGAATGAATGGATGTACTATATCACTGGAGCCCTGTAT
TTTTTTTCTTCAACGGTCAATCCAATATTGTACAATCTGATGTCAGTAAAATACAGAAAGGCCTTT
AAACAGACACTTTTCGGGGGGCAAAATGTGAATAAAAGGGGTCATCAAAGCAGCTTCCGAGAATCA
TCTCAAGGAGCAGCGGAAATTTCTGAGGATACTCAGAAAAATACCAATAAATGCACTCCATGTGAA
AAGGTTAAAAGTGATACTATTGTGAGGGTTAACAATAGCAATGTAACCAATGGAAATTGCGTTTTG
ACAGTAAAAACCGGATTTTTTCCTAAGAATCTCTTTATTCTTCCACCAAATCCTCTCGCTAATAAA
GAACAGGGGATAAATCCTCAGAAAATAGGAACACCGCCATTATTTATTTCCAATTGCAACACTGCA
AAAGAAACTTATATATAAATTATTGCGATGCCAATTAATAAATCTTACTTTATATCATTTATACAG
TTTATTATTCAACGCTGGAAAAATATTAGATACAAAGCATAAATAAAATTGGCAAGTTGTTAGGGA
AGTAATTCATCAAAAGCAATGAAGGTTATACGATTGCTTCCTATCTATCAATTTCAGAAATTTATA
ATAGACCAAGTTTAATCTGTATTCCTATTATATTGTAAACAAAAAAATATATACATAAATATTGCT
GTGAAACTGTAATTAAGCCTCTGTTATTTATTTCAAAGGTTAATTTATTATTAAACGCAATGTAAC
TACTAGACTTAATTTGAACACTTAATTACTGATATTGATATTTCTGATATAAATATTGCTATATAT
TTTATTTTTA

Pyrokinin receptor

>Nv_A14
GGAGGTCTGAGATCGCCAGCGAGAAGAGATAGTAGTTAGTGGCAGTGTGCATGTGCCTGTTCCTCC
CGATGACGACGCAGGTGCTTACGTTTCCAATGAGGCCCGTCACGAAGATCAAGGAGTAAAGTATAG
TCATCGGCACCACGATGTACAGCGGGTCTCTCTTCGGCCAGTCCGTGATGGTGTCATTGAAGAACT
CGTAAGTTTCAG

>Nv_A15
CGTCAAGGACCTATTCATATCACACTTTCCTCCATTAACAACAATAGTATATCAACTAAAAGAATA
ATAGATATCTTTTATCTTGTATCTGGAAATTGTAAATTTCTTTAAACCTTTCTCCATTTTGATAAC
TCTTTCATAGTGTAGATTTCAACTGGAAAATTAATTAAAAGTCATCGTTTTTCAACTAAAATATAG
AAATATTCAATATACATATTAACGAAAATAAAACTACTCACTTTTTTTTTAAATATATTTTTTTCT
GTCCATGAATCATTTCTTTCACAAGGATTTTTGTAACATTCCGTAGAATTTATTTCTCTGACGGGA
TCAGAATCACTACACTATAGTTTTGCTATCTATAAGTCGCTTGTTTACCTATAATTAATATTTTGC
AAGTACCATCTTGATTAAGATACCGTTAACCATTCCTCCTGTTGCAGTCGCAGTTGTCGTCGGTTT
CTTCATCTGCTGGGCGCCCTTCCACGCACAGAGGCTGCTCGTCATCTACTCGCCCACCGTGGACCG
CAGCAGTCTCCTGGTCACAGTCTATACAGCACTCACCCACATCTCAGGTGTCCTCTACTACCTTTC
CACCACCATCAATCCTCTACTTTACAACATTATGTCTCTCAAGTTCAGAGAAGCTTTCAAGGTATG
TATTCACTTAGAAGTAAAGAGTTAAGTTCGGGGTATATATGACAACACGTCCGAGCCTCACCATGT
CCGTATGATACCAAACATGGCTGTAGCTAGTCCTCATATGTTCTAACCTAATCTCAGTGAGTTAGG
TTCCATACAAGCTTGGTATGGTTCGGACGCGGTGCGATCATTTGTGTCCTAAGATACTTTTCTATT
ATTGCCCTGCCAGTAAAAAAACTATTTTTTTTTAAATTATAATTTCAATATATGCCATAAAAAAAA
C

Sulfakinin receptor

>Nv_A16
AAATAAATTTTTTTTTTTTTTGTGTGCGCTCTCTGACTTCAACCCCGAATAACTTCCCAACCATAA
AACATATTATTCATTTATAAAGTTTCAATACATAATGTATGTTTTTCTCCTCTGTATTTAATTATA
TTACATTTTTAAAATATTCAAAAGGCTGCTTTATGCAATAAATAAAGAAGCTATTTCTTTGCTGTT
CAAAAGGAGTTTCACTGTACTTCTACGTTGCGACATGATTTTCTCATTCTCTTATGCTCACGTAAA
ATGCAAACCTACGATCTCTTATTCAGTACCATCTTTATTTCCAGGCCAGACTTGAATAAACACTCC
AAGCTGAAGATTACACGAACAATTCTTTAAAATTGGTTTCTGTTGAATTCTGTCTCTTGGATGTTT
CCTTGCCTCTGCAGACCCTTGCTATTTCTACTGCAGCTCAGCTTTATTGACTGTCAGCTGTTCATC
TTCTCTTCTCGAAGGCAGCCAAACAGCTTGAGGAAGGCCTTTCTAAAACTGGAGTTCATAAAGCAA
TATGTGATCGGGTTGCAGCAGCTAGAGCAGTAGGCAAGCAGGTGAAGGAAGCTAATACCTTTGTAG
CCCAGTCTTTCGTAGACAGCCGCTGGAGCGAACAGAGCAATCGTATTAATGGCATAAAGGGGGGTC
CAACATATAAAGAACTCCAAGACTACAGCAAATAACATTTTTACTACTCTCTTCTTCTTTTTGAGT
GCTTTCTCTGTGTTTGAACGCCTCAGGAGGTTCGGGCTTCCCCTTCTGGAGCTGTGATTCCACGTT
ATCTTTTTGTGTTCGTTGTTCTCGTTACCCTGAGGAGGCTGCTTCTTGATTACTCTCCATAGGGTT
CGCGCCACCATCGAGTAAGTTGTGACGAGTACAATAAGAGGAACAACAAGAAGGAGCATGTCGAGG
AGGATGGTGTATCCTTTTTCGTAGTCTATGGTTGGCCACCGCTCCCGACACTTTTTATGCCCGTTG
TTGATCGGAATAATTTCGCTGAAGGCTGGAATCGGTGACATAACGAGGAAGCTGCCAAACCAGATG
GCAGCGATGAGACGATATGCATGATCCAGAGTCTGCCATGCCCGCGACCTGAGAGGGTGGCATATG
GCGTAGTAGCGCTCAACTGAAAGGCAGACTAAGGTCCAACCGCTCACCGCAACTGAGGAGGTGTTT
TTACGTAGATCGTTGTAGGTTGCGAGATCACATCTGCACTGGCTTGATTAGTATTATGACATAGAG
CATCAACTTCAGTTGATAGAACATTAAAGGGATTTGATAATTCGAAACTAACCTGTCTCTCTGGAT
CAGTAGTTGACGAGACCAAATGACACACAGGAAACCCCTAATATCATTGGTAACATCGTTGGTACA
GCATGTTCTTCCAAACCTTACCTAATGAAAGGACTAAAAGTAATTCTCTGGAATACTAATGTTGTA
TTATAA

Rfamida receptor

>Nv_A17

TGAAATAATGTTTCACTTTTATTTCATTTAATATGTATCTAATTCAGTTGCTTTATTAATAGTTGA
AAATAATTAATTCTCATATATTCTGTAATTTTTTTATTTATTCATAACAACTGGTGGGCAACCCCC
TCCAGCTTGGCATCCTCCCTCCCCCTTAGGGCCAGCACTGTGTCTCACTATAAATATTGAATAATA
AATACAATTTATGTTTAGATAATACTCTAAAAAGATTTATTTGAATTTTATACCCAACACTGCCAG
TAATTTTCCTATTACTTCAACTGAATCACAGTTATTGTCTGAAATGAACTGTAAACTCGAAAAAAA
TATTTGAAAGGGTTATTTAACATTCTGTACTTTTTTATCTTGATTCTAAAATCATTTCACAATCAT
GGGAAAATCATTAAATTATTTTCTTGATAAGCAATATTTAAATTTTCATGATTAATTTTTGAAGAA
GTTGCAGTCAACTAATGCGTTATTTTACTTAGCTTTCAAGGAAAAGCATATTTTGTCCAGAGGCAA
TTAACAAATTACAGCCAATTGATGAGCAAATTTAAATAGCAAGTAGATGGAATATTATTAAGACAT
TTATTTAGTTATTTCTTTCTTTAGAAAAAAAAATAGGAATTCCTATTTAATTGAGAAATTATAGGTA
AATAAGTAAATATTGTTTGAGTTTTAACATTTGAATTCTACTCATTCAAAATTATTATTGATATTC
CTGTGGTTTCTACTAGGGTAGAAGAAGTTCTAGGGAACAGATTTCAAAGGGAAAAGTCCGGATATT
CCAAAAAACAAAACAAAAAAGAAATGAATACTGGCCAAATTGAGTTATATACTGAAATCTTTGCCC
CAACTCCTGCATATAGTGAGAGAATCATTAACTCACAACTCTAATTTTTTAGAGGAGAGCTGTGAG
TTAACTAATTGACTGCTTGAAGACTTCACATATGATCATAATTCATTTGGCCATTTTTCAACTTCG
ATTATTTCCTTTGAAAGCTCAAAGATTTATACTTTTATTAAAAGTTAATGGTAAGTGTTCAGAATA
TTTTGGAGCAGAAAACTTTATGATTTTATATCAATAAACTCATTTTAAAATTTTTAACTTTTATAT
TTTCATGGGTTTTATTATTATGAATTTCGGGGAAAAGTAATTCAGTAAGAACATAAATATTTAATT
ATATCATAGCCTCAGTAAATTCCTTTACATTATTGACCAATCAATGGATCATATATTTAATGGACT
AGGACCTAGCTACACAGTAACATCTATCATAGTGGGTAGGATAAGAGAGCTAGTAATAGTAAATAA
AAAAATGAAAATCTAACATTCCTTATTCAGGAATTGTAACTATCAAACAAACGCAAATCATTGCAA
TAAAAATAATTATGTTTTTAGACCTATTATATTGTTTTTTGAATTTCGAAAAGCTTTACTTATTTA
TTGGTAACGAATGAAAATGAAATACAAAATTAATTGTTTTCAATATTGAAGAAAAAGGGATTTGTG
GCAAACAACCAAGGCCAAAACAATTTTTATTATGATTTTATATGTGTAAGCTATTATAATTATTTA
TGTAGTTTTACAGTACCGATTTGTTGTACTATTTGATGCCCAGCTCCACATTTAGAAAATATTAAA
TTGGTTGAAAAAAAAAATTGAATTGAATTGTATTTCTTGAAGTTAGGAAATATGGATGGATAATCTT
TCAATGTACAAATGAGGTTCATTCCATAGCCAGAACTAGGATATTTGCCGGATGGGGAACAAGCCT
AATTTTTTTTATCAATTCTTTCACCCTTCCTCAAAGTTAAATATTTAATATCAGTACAATATTTTA
GGTAATATATTCAGAATATATATCATCATCTCATTATACTAACTCATTTTATAGGCTTAATCAAGA
TCAAAACAATTAAAAGAGATATTTAAACAAGCAAAAAATAAATATTTTTAAATTAACCTAAAATAA
AATTTTGCTTGAAATTGGTTGTCTTCCATCCCCAGAGCAAGGGGCATATCTGGCTTCTTTAGTTCC
GGTCTTGTTCGATACTTTCATCAGTATTTCTAAAGTTGAAATTTAATCTTTCTTGTGCTACATAAA
ATAGAAAAAAATATTTACCCTCCAAAGCTCAGAAGATAATTGTTAAAGCATTTAAGACAATTATTA
GTTTGTTTTTCATTTGGTTAATAACACTAAAATGAATTTCAATAAATTGAGAAAATAACTAACCAT
ACAAATACTATTTCTCTTTTCATAAAGTTACATAAGAGGTATTGTCATGGGAAAGTTAAGGAATAA
AATTTACCTAAATAAGATGAATGTTCTTAAATAAACATTTTATTATCCTATCTTATGATTAAGCTG
TTATCTTATCTATAAGTTTATATCTGATTACGATGTAATTGTGTTTATTGATGGCCATGGTGATTT
AAAATCATGAAATGCAGAAATGATTGTTCAAGAAATTGGAGATATTGAAGAACCAATGACCAATAG
TCACTTTTTACATAATTTATTAGTCATTAAGTTCTTTACCTTCTTCTGAAATAAAACTGATATGTG
TTTAGTTGATTTTATAAAAAAATAACCTTTATCATGAATTACATCAGCAGTCAAATTATTAAAATT
AATAAGACGTTGAAATGAATAAAATGACATACCCTTCATTAATGACATAAAATTTAAAATGCAAGC
TCTGAAATAACTAGCAGATTCCTATAACATGTTATATATTTACATTTCATGCAGCCTATCATGATA
CTAAAGATTCTATACAGATCAACTATAATTTAAGAGGAAAATTAGCCCTTTGGATTCAAGGGCACC
CTGAAGCTAAAATTATCGATATGAAGAATTACTTATATTGCGTACATGATATAAATACATTTTTAT
ATTTACAGGCTTAACTATATATATAACATTAATACCCTTAGTTTTGGTAATGAGGTTTGGGCAAGA
TAGTGACTAGTACAATGTTCACACCAGCTCGACTAACATTTTTTTTTTGTTGATAATAGTAGATTA
TTTATTTACAATTTCAGTTCTGAAAATCACCTCCTAACCTTTAAATTTGTACACTATTATTTTATT
ATATACATTACAACATGTAAAATTAATCAAGTACATACAATGCATACATGATCCCAAAACTAACAA
TTAGATAATAGTACTAAAATTATATAATTATTCTGGTATTTTCTTGAACATAGGAATTGATGCTGG
TTCAAATCTGAGCTTTACTACTCATGGCCGGTAAAAGCTATTTAAAAATGTATTCACTGGCTTCCC
ATTTACTCAAAGATTTTCTAATTTTGTTTAAAATGCATTATTAATTTCAGTTTGGCTTTTGCTCAG
TTTTTATTTTAACCTGCATTTAAAAGTTGTCCGATTAATAATACTTAACAATTAGTATAAATCTAG
CCAACAAGCATTACTACTGTGTATTGTATAAAATATATTTTTGTCAATTCATTTTTTTTTTTTTAA

TTTTTTAAGTGTTTGAATAGGGAAATTATTATCTTTATAAAATTATAATTTAATTTAACAAATAAA
ATAATAATGTAATATATTTGTGCTTAAGGTGAAGGTAAGTAATTTAGGGATCCCTAAGACCAGAAA
AACAACTTCACCACTCAAACAGAAAGTTTTTTTGGGTGTGAGACCATCATCAGAGGATTTGGTAGA
AGAAAAAGAGCTGCTCTGCATAGCCATTTTTTTTTTTTTTTTTTTAATTTAACCCAGCTATCTAC
AAAAGCTTTTCCTTTACACTATCAAAACAACTGATAATGGCCTGACAGCTGAAATGTCTTGAAAAT
AAAAAAAAATTGAATGTTGAAGTTCATCTGGTCTCAGGAATCCCTAGTAAAATAATACTTCTATAT
TTTTATCTTCACGATATGATTGAGAATATGATATTGGAGATTCGAACTGGTGCTAAATTTTTAATG
ATAGAAGTTTTAACTGAGTTGCTTAAACATCAATATAGCAGAAATTTACTTTAATAAGGATTTTGG
TGGAACGAGGACATATTGTTCCAGAAATACAGTGATTGCTTAATGAAAAGTTTTGTAATGGACTTG
ACAAAGCAACCTGATTTCTCTCAATAGTTTAGAATAGACCGCCTCTTTTTCATTTTTTTTTTTCTTT
TATACAAATCACAGACATTTTATTCTAAGACTAAACACATAAAATAATACATTTGAAAAATATATC
TCTAATTACATATAGATAAAACTATTAAGTTGAAGGTACAGAACTTTACAACACTTAATTTAACCT
CTGAGCATAATCATTCCATAATGTAGTTTGCCTTTAGGCTTTTGATACAGAATGTCACTAGGCCTA
GAAACGACAAGCAGTTTCGCCATCTTTTTAAAAACAGTTTTGTCTTTGATAAATTACACTTTGGTG
TAGACTGTCATTACCGAATCTCTTCTTTGGGACGAAAGGTTCCGAAGAAGCATTGCCTGGTGCTAG
TTGATATAGGCCCGGGTGTCCTGAGTCCTGAGGGAGGTTGTCCTGGTGTGGGAGACGGAGAGCTGG
CGGCGGGGGGCGCGCCCCCTGCAGCAGAGCGCCTTGCTGAAGCTGTCCCTGAAGTTCTTCGACATG
AACCCGTACACGATCGGGTTAACGCAGCTGTTGAAGTAGGCCATGAGGTGGAAGGCGGTGGCCATG
TGCTTGAGCTCGTTGGTGCGGGTGTGGGGGAGGACGTTCCAGGCGGTGAGGAGGTTGTCGGTGAGC
ATCGGGCCCCAGCACACCACGAACACCACCACCACTGCCACCAGCATCTTTATCACCTGTCTTTCG
CTCTTGTAGTTAGACATCTGCCTCACCGTGTGGCTGTCTTCTTCCAGTCGCATTTTCGGTTTGAAA
CCAGTTCGTTTTGAGCTCAGCGGGTAACTCTCGCAATTCAGAGTTGCTTTTCCAGATGTCATGTAG
GACCTGCGTTGTATGACCCTGCAGATCTCCCAGCAGATCGCGGAGTAGGTGATGGTCATGACCGTC
GTCGGGAAGACCAAGATGAGGAGGAACATGTACAACTCGTGGGCTCGCCACACTCCAAGCCTGTCC
CAGTCACGCACGCACCAGAAACCCTTGACCCTTTCTCCAACGCCCATCTGAACCTGTACAAATAAT
ATTGGAACTGCAAGAAGAAAAGATGCTCCCCAAGTTGCAATAATTATTTTTTTAGCTTGGCTGATG
GTGCAGACATATTTAGCCTTCATCGGGTGAACGATAGCATAATACCTCTCAATACTCATAGCAGTA
AGCGTGAGGACAGAACAAATGGCAGAAACTCCCTGCATGTAATGAACCATCTTGCACAGGAATTCA
CCCATAGTCCACGTATATGAGAACAGTTTAGCAACCTTAACAGGAATGCAAATCAGGATGAGGAGC
AGATCCGCCGAAGCAAGACTGGCCAGAAACATGTTGGTGGTACTCTTCATCCTCCTGTACACGGTG
ATAGTGAAGATGATGAGGCTGTTGCCGGCGATCCCGATGAGCAATGTGATGGAGTACACGATGAGG
GTAGGCATCAGCTCATCCCAAAAGAATGTGTTAATGGATTCATCAATATCATAAGTGTAGTAATCG
TAGAAGGTCTCTGTCGTTCCGTTTTCAGGAAGGTCAGTCGTAAACTCTGAGTCGTTCATCTTGATG
GCGGCCTGGACCAGTCCTCTTATCAATACATGATCAAAAATCCGTTGCAGTGACTTCAAGCGACTA
TAAGCTCAAAGAATCTCGAACTCTCGTGCAAAATTTTGAGGGTGTTTTTTTGGAATTTCACCCTCT
TGGATTTTAATTATCCTTGTTTTGTTTTTGATTTTTGCCTTTGGATAAGAGCCGTGACAGACTGCT
GATGGTTCGGCCCCGCCGTCACGTGGAACGCGATCTTCAGAGCACGAACAGACAACACTCACCCCT
GAGTACTGAG

Kinin receptor

>Nv_A18
TTATTTGTTTAAGTACAGATAATGAACAACTTTTTTTTACACAAACAATTTTAAATACACCATATT
AATCTACCATCTATCACTATGTAATGGGTAATACAATGGGTTTATTTACATAGATATTTTGTTTG
GGAATGGAACAGAAGGGAAGACAATTCAAATCTAAACTCTAAGGCAATAAGACTTTTCTACATACA
GCTTACAAAAATATCCATAATTTCTAGAAATTTGACAGAATTTTACCTCAGATTTTAAAATCAAAT
CTTTCAAAATATTTTCACTTACTAATATAGCTATCAATGTTATTAAAATTTCTAATGATTCTTGCA
ACAATAATATGACTAGTTACTTAAAGTGTATGTTGGAATTTAATGTTTGGCCTTCCATTTTACATT
TGAGCTGCTATAGATGCCACCATCAGTCAAAGTTTATTTCTTAAAAAAAAAAAATGAATACAAATAA
TGAGTTGATAAATTAAAATTTTAATATTATGTATTGTGATATGTGTATGATTAATATAAATGTTCA
ACACATATAGAATCATATTTACATGTAGTGATTATATACAGTTGACATAAAAATGAGTAATTTTTT
TTTTTTTTTTCAATGTCCAGAATCCCCTGGGTTAATATTATAAAAATAGCTATAACTGAATACCA
AAAAATTATTGTTTTTTAAAAATTCAGTTTTTGACTAGTGTATAGACTAGATTAAATTATTAATAT
TTTGTAACAGATTGTTTATAAATGGACCGATTAAGTTCATATCCTATGACAGCATTTTGATAAGCA

TTAGTAATTCTTATCTATTTATCAATAGCTCAAATTATAATCATTGTTTCTGTAAATCGTAGAGAG
TTAATATTGAACATTATTTTTTATATAGTAACGAACAAAGTATACAATATTGTACAATATCTGTCG
AAAAAAAAAAAATTTATCTGTGAAAAGAATGATCTCCATTTCCTATGAAGATCAGCTTCATCAATCA
TTCCAGTATCTTTATTCAATTTCTCTTGTAATAATGCAAGGTGAAGTAACTGAATAATTGTTTACC
TTATTATTATAATGGTGCTCTTTTATTCTTGTTCTTTTTAGTTATCTTTATTTTCTTAAGTTTTCA
TAATTTACTAATGCCATGCTTACCTGTAAAGTGATTTCAATTTTTTCTAGTTCTTTACATTTTATA
CTCATAAATTCTGTTTTTGGTTTCGTGTGACATATTACTAATTATATTTTATTCAATGTGATATTC
ATAAAATATTTACAACTTCGGGAAACATATTAATTTAAAATCAATAACTGGTTATTCAATACAGCT
TCCAGTGCTTAAGTACACTATAGAAAAGGTTTTATACAAATATGATTATTTATCAAGTAACACAAA
AATATAGCTCTTAAAAGAAGATTTATTTACATTTTTATCTTCTATAAAGTGAAATAAACTATTTGA
AATTTTTACCTACTTTTAGTTAAATATTAATCACAAAAAGTAGGAAAAATAAAATTTCTTTTCTAT
TGGATTATGTATGTATTTATGCTGATTTTCTTAATACAACATTTTTTTTTTTATTTCTATTCACTT
CTAAAAAGAATACATCTTTGCAAATTATTCTAGTTAAAACATTAATGATTAATTACAACTTTTAAT
TAAATTCGTAGCTTTAAAAAATAAATACAAATAGGAAATATTTAGAACATTATTTACACAATACAA
CTTAAATATACTTTGGTATTATTTTGCCTGCATCATTTTGAAAAAAATGGTTTACACTTTTAAAAG
AGATACAGACAAAGTTGCTGGCATTCCATTTTTCATTGTTGATGCTCGTTTCCAGTCAAATCGAGA
ACTTTCAAATCCACTAAGTTCCCTCGTGAGTGGATCGCCCCTATGCTTTTTTTTAATGCATGGTGC
TCTCAAACGAAGCTGGAATTCTCTTTTAAATTTTTCATTATATATAGCATAAACAAACGGGTTATA
GCAACTGTTACTCATCGCTAGCCAGTCGAATCCAAACCAAAATATATTGATGTATTTATACTCATT
TATGTCAGTAATGTTTTGTAGTACGTTATAAGTCTGAAGTGGGAGCCAACAGAGAGCAAACAGAAC
TACAACTATGACAAGCATCTTAATCACCTTTTTCTTATTCCTCATGATGTTTGAGTCACGATCAGA
CTGTGCGTTACCGGGAGCTGTGGATCCCCAAAGTGTGAGAGCTACTCTTGCATAAGCATAACTTAT
GACGATCAGTGGCAAGAAGTATTGCAAGAGTACAAGGGTGGTTCTATAGCTCTCGTACAGTTCTGG
CGGCATGTTCATGTTGTCGCAGTAGTCAAAGATTAGTTCAGAGCTGTTGAGAGGGAAGTAGGGTAC
AGGTGCCTTGGTGACCCGTAGGCCGTAGGCGAAGGGAGCTGCCATGACACCGCCAGCTATCCAGAT
GACCGATATCACCACCCTGAAGTGGACCCGAGAGGCTCTTGCCGTGAGAGGGTAGAGAATTGCTCT
GTAGCGGTCGAGGGCGATGGCCGACAATGTGAAGATTGACACCGATATGCTCAACGTCTTGACGAA
GGGGCAGAATGGGCACATGAAGTTTGGAAGCACCCATCGCTGGAGGAGAGCTGCCTGGAACTCAAA
GGGTATAGCGAAGAGGCCCACTGCTATGTCTGCCAGGGCTAGATTGGCAATGTAGTAGTTGGTGAC
ATTGCGCATTCGCCTGGAGGTGAGTATGACCCATATGACGAGGCCGTTGCCGACGACAGCAGCTAC
GGATATGGATATGTAGCATATGGAGAGGAAGACGGTGAGGTAGGTCGGGGGCTCGTAGAGAGGCTC
GTAA

SIF-amida receptor

>Nv_A19

TAGACTTTCGGTTAAATGTGTTCCATTTTATACATTTTTTTTTTTTTATTTCTTGAAGTTTTACCGA
ACTCATTTTCCATTCACTGTTCCATTTATCTTGAAAAGAGAAGACAAGTTGGTGTGCCCAATCTTC
TCGAGGAATGGTTTCAATTATTGTGGTTTGTGGAGTTTCTTGATGCCAAGCAGCATTGGCAGCAGC
ATCGGCTCTCTCCAGGGGTGACCATCCCCCCACGATGGCGCAAGAACCTACTCCCTCCCTCTCCCA
AGATATTTTATTGCTCATTTTTCTTTAAAACAGAGCTAGGGAAAGTAGAATTATAGAGAGGAGCAA
ACCGATTTTTTTGTAGTAATAGGTCAGCTTTTAGTGTCTTCTGAAAGGTTATCTATATATATATAT
GAAATATATATGAATATTTCAGTAACCCGGTTTCATACTCAAATTCATTTATTTTCTTAATGTGAT
GTTCCGAAACAGTTTTCAATCATCAAACATTATAATTAATTTTTCTTTATGGGAAGGATAGGAACT
ATCGATGGTCATTAGAACTAACGACAGGGTAGTTTAAAACTATCAATATATTGATAGCACTATCGA
TTATAGCCTATCCCTAATTTAGGCACATCCATTCCTTCATCTCCGGATCCGCCCTTAATAGGTGTG
GAGATGGCTTTAGTGGTTGAGTGTGAATACTGACCTCATTACTTCTTTTTGTAATCTTTGGTAAAG
AAACAGTACTTTCAAAACAAGTTTTGAACCCAAGACCCATTCTTAGTTTTGATAAAGATACAATCA
CTGAGGGAATATTGTTTACAATTTTACCTATTAAAATACTAGGTTCTTGGCTATTTGGTGGCCGCT
CAAATGTCAAATAACTACTCGACGGGCACGAATCATGATCGCAATCATCTGGTTGATCGCAGGGAC
AGTAACACTTCCCTGGGCAATCTTCTTTGAACTGGTCAAGTATGATGAAAACAGTGACATCATGTT
TTGTGTAGAACAATGGCCCGAATATTTTAATGGCCATCTTTACTTTCTTTTTGGCAATTTATTGTT
CTGTTACATACTACCAATGATCCTCATCACTCTGTGTTATGTTCTTATATGGGTCAAGGTATGTCA

TCGGCACATACCCAGTGACTCCAAAGATGCTCAAATGGAAAGAATGCAGCAGAAAAGTAAAGTGAA
AGTAGTTAAAATGCTGGTAGTTGTCGTCATCCTGTTTGTTCTTTCATGGATGCCCTTGTACATCAT
CGTAGCAATGTATAAATTTGGGAGAAACAGAAGTGAGTGGGAAGATGAAATGCTGAATGCACTCCT
GCCAATCGCACAATGGCTCGGAGCTGCCAACTCCTGCATCAATCCTGTCCTGTATGCTTTTTTTAA
CAACAAATTTAGAAGAGGCTTTGCAGCAATAATTAAAAGTAAAAAATGCTGTGGCACGCTTCGTTA
CTATGAAACTGTTGTCCGTGCCAACTCCACCTCTACTAGTCTCAGGAAATCTTCTTATTATGTGAC
AAACAACAACAATAATTCCTCCCGTAGGCAACTGAGCCTAGACACCAATGTGTCTTACATATCAAA
TAACTCAGGGGTCTGAGTGCTCTGTTACCTAATCAATTGTGGATTCCATTATTATAAAGCGCTTG
GTAAAAGCGTGAAAATTGTGGCTCCAAGAAGCTGAATAAAGAAACAGGGTAATCACGGTCCAAACC
GAAACACATGACACAGCTCAAACTTGGAGATTGCCCAAGAGAAGATTCTCACTACTTTACATCAAC
TTAAAAGAAATAGATCTCTTAATGGTTTCAACTTAAGACCTTCAAAATATAATCTTTCAATGCTAT
ATTTTTATCTGACACGTGTTTTA

Allatotropin receptor

>Nv_A20
GCATTGTGTAAATAAAATAGTTTCGATCCGAAGATCGTCTCTGCTGAATGTAGTCAGTGTCATCAT
CTCTGGCAGGTCTAGAACCAATGCGATCATCCATATGATGATGATAGCACTCACAGCTCTGGAAGT
TGTAGAGATAAACTTGAGTGGAAAGCAGATGGCGTACCAACGGTCAACCGAAATGAAGGTTAACGT
GAGCACCGATACGGTCACCGAGACAGTCTGAAGGTATAGGATCAATTTGCAAAGCGTCTTTCCCAA
GAACCATGTCTCAGTAACGTCCCAG

Trissin receptor

>Nv_A21
TAGAATTAATAACAATTATTTTGTACGAAATATTTTATTTGGTACACATTCCACAGCAATACAAAA
TCCATATAGATACACAATAAATAGGAATAAATAAAATGATAATTTTTTTTTTTATATAAAGAAATA
TATAACTTTACACATTTACAAGAGGGAGGGGGGAATAGATCGTTATATTTATAAACTCCTAAATAT
TTATAAAGAGACCCTGTACTTCTAGATAATTTAGCCAGAAAATTCCACTACTTGTGAGATGATTGA
CATGTTAGGGATTTTGCACAATTAAATTCCCATAGAAAAAAAATTTCAAGTGCGCAAAAATAAGAC
TACTGCACACATACTTAGGAGATTTTTTTTTTTTTAAATTTAAAATTATATTTTTAATTAGTTATA
ATTTTACACTTTTTTTTTCTTAATGCCTTAAGGTATATTTTTCCATTATTACTAGACATCCTTAAC
CTAATAAACTCTAGACAAATCTAAACAAAAAATGGGTAGTATTATAATTTGTTTAAATTATAAATT
GTTAAAAAATATAAAATTCTTGATTAAAAACGAATGACTAGATTGATAGTAGTATAAAATCTCGTA
TCCCTCTAACTTTTATCACATGGGTGAACCTCACTTATTTTGGTTTATCCAATCTATTTTGTTCTT
ATTCAATTAAACCTAAATTACAAATATTTTTCCATATTTTTTGCAACTTTAAATGCACAGTTTCAC
AATATGTCTGACACGTAAGTTTTCTTATTGAAATAATATTTTGGTTTTGTCTGATTTTATTAATAA
ATATTTTTTATAAATTGAGTACATTAATTATTTTGTATGGATAGAAAGGTGATTAATTCATTACAG
AAATTAAACACTTGTTCATTTTATGATGTACTAATAAATTCATTTATAATTAAGAAAGAAACATAG
GTTAAAGAGATTTTTGGAAGAACTTTGGAAATTGTACCGTATTTGAGTACAAAGTATTTCAATTTC
ATCACAAATTATTTTTTGTGTTTAGTTTTAGTTCCAATATAATTTCATACATTTAAATGAATTTGA
AATTTTACACTGTATTTAGATTTGAAATGTCATGCCAATTCATTAGGAAAAAAATATGACATAAAA
TGATATATTTTTTTTTAGAATTTAAGCATCTTTTTTCTGTTTTAATGATAAATTTAGGAAAATGT
AATTATATTAAGGTAGAACGGATATTAATGGAATAGTTTCTGATAGAAGATTGAATTTTTATGTTT
CAAATTGATTCAGATTTTAGTTTGATAAATGTTTACAAAATTTTGTAAATTATTGCTTGTGGTTTA
TTGCGAATTTTTAATATCCTCTAAATAACACTTGATTTTTCTTTTAATTTTTCTGTAGACATTTTG
ATTATTAAAAAGTTTGTTATCCTGTTGCCATAGATATTGTAATTTTCGGCTCAAATCTTGATAATT
GAAGATAGAAAATAACGTTCACTTTATGTTAGATATAAATATAAATAATGAGAATCATTGGTTTTA
TTTATTTGTCTTCGGGAAGAGAGTTGACCCCCAAAATTATTATATAAATATATCAGAAAACCCACA
AAATAAGAAATAAAAATTTAATATTATATTTTATACATTTCTGAAAGTGGAAGGTCTATGGGGTAC
ACTTGAAGTAGGTATCTAAAGCTAAACACTGTTGATTATGTACCTGGATGGGTGACTGTCCTCGAA
AATTGGTCAGGAAGGGACAGGTTGTCACTGCTTGACGTAAATCATGGTCTAAAGTAATGTGAAGTT
GGGCGACTCTCTTCCAAGTAGTTCGTATACCCGCTGGAGTACATCTGTCCTTTAGATTTACTGGGT

CGAAGTACACGCCTTAGTCTAGTACTGAAGTATCCTAGACCTTGTCGTCAGTATCCGATGGGGGCT
GTGGCTCTGTGGCCTGTTGCTGCTCGGCGACCCTTGGGACCCCCACTTCCTCCATCGTCGTGGCCC
TGGCCACCGAGTTGTTGAGGTGGATCTCGACGGCGGAGGAGGCGTTGGAAGCCCCGGTCGTCCTCT
GCCTGCTCACCCGGTTGGGCCCTCGAGAAGGGAGGGCCAGGACGATCTGGTGGCAGCATGACTCCA
GTGTCTTCTTGAACGCCGCCCGGAACTCTCTGTTGAAGTAGGCGTAGATGATGGGGTTGAGGGCAG
AGTTGAAGTAGCCTACCCAGAAGACAGTGGCCACCACCATGTGTGGGCAAGAGCACACGTCATGTC
CGCACAGGGATGTGATCACGTACCAGAGGAAGAAGGGAAGCCAGCAGGCGAGGAAGGCAGACATGA
TGATGCCCAACGTCCTAGCGGCCTTCCGCTCCCTCTTCATCTTGCTGGTGGTAGCCAGAGGCCTCA
GGGTCTCCGGGCTGCCCACGTTCACCCCGGCCGCTATGCCGTTGATCTGCAGGTGTTTGTTGAGGA
GAGCTGTGGCCACCTTACTCCTGTACAACATTCGCTCCTGCCTATCCGCTTCGATGTAGATCCTGT
AGTACATGAAGATCATGATGACTCCAGGCACCCAGAACGACACGCTCGATGATATCACCGCGTACG
GTTTATTCACTAGAAACTCACACAGGTCGGGATGCAACTTCCTGTATTCCAGGTGTTCTTGCGTCG
TATACCATCCCATGAAGATTGGGAGGAAGGACACGAGAGCAGGGGAGCACCAAACCACCGCGAGCA
TGATAACCAGTTTACGCTGGGTCATAATGAGCGGATAGTCAAGCGGTTGTACAATGGCATAGTACC
TGTCTACGCTAATGCAGCAGAGGTGGAGTATGGACACTGTCGAGAAGTAAACGTCCAAGGAATTCC
AGACGTCACACATGAAGTAGCCGAAGAGCCATCTTCCGGTCGTCTCGACACTGAGGTTGAAACACA
TGGCCCATATGGCGACGAGCATGTCCGCCAGGGCGAGGGACACGACGAAGTAGTTAGTGATGACGC
GGAGCTTGCGGTGGCGCATGACGCTGACGATGACCAGAAGGTTG

Tachykinin receptor

>Nv_A22
ATTTAGCGATATACTGGCTAGCCATGTCCAACTCAATGTACAATCCTATGATATATTTTTGGATGA
ATACCAGGTTTAGACGTGGTTTCAAACAATTCTTTTCATGGTGCCCATACGTTCAAGTACCTCCAG
AAGGTTTAACTCGAAGGGAAGCAGTCACCACGAGATTCAATTATTCTTGCTCAGGTTCACCAGAAG
CCAACTATCGCATCAACAGAAATGGAACTTTACAACGGACCTCAATGGCTAACTCATCATTTGAAA
CTGAGAAGACAATTTGTGAAAGCTACAGCTATGCTAGTTTTAAAAGGAAAAGATGGAATTCTAACG
ACCCTTCTTGACAGGAACCTCGCATCAGACGGACAAATTCATCAGGACAAACATGGATCTAACTGG
CTAGTGATGCTGG

>Nv_A23
ACCTTCAACTACACCTACATGGTCAACAGCGATTGGCCCTTCGGTCGCCCCTATTGCAAGATCAGC
CAGTTCGTCGCTGTTCTCTCGATATGTGCCAGCGTCTTCACTCTCATGGCCATCTCCGTTGACAGG
TACATGGCGATCATGCACCCCCTGAGACCCCGGATGGGAAGGAGGATGACGCTCTGCATCGCAGCC
GGGATCTGGGTCATCGGTTCGGCCTTCTCCCTCCCGATGCTCA

Short and Long Neuropeptide F receptor

>Nv_A24
AGGAGTATAGTGGTGTGAAGGGTACGGCCAACGTACAGAGGAGGATGTCGGAGAGCGCCAGGTTGG
TGATGAAGCAGTTTGTGACAGTGTGCATCGCCCTGTTCCTTCCGACGACGTAGCAGACGAGGACGT
TCCCGAAGAGGCCGAGAACGAAGATGGAAGTGTAGAGGAGGCAGAAGACGGCCTGGACTGCCTTGT
CTTCGATGATGTCCAGGCCGGTTTGGTTACCCACGCTTACGTTCATCTTCCCATCACACTGTTCTT
CCGTATCCTAGAGCCATGCCCGCCACCAGGCTTAGAGACGAACTATAAGACACGACCAGAGGCGAG
GACGGACGAG

2. Neuropeptide receptors Family B

Calcitonin-like diuretic hormones receptor

>Nv_B1
AGCCGTCCAAGAAAAGACAGTCAGTATCCTCACTAATTTCTGCTCGCTTGTGAAGGCGGACACCAG
GAGAGTGTGGAGGTAGACTCCCTCAGCCAGCATCCAGGCATAACAGCTCAGCAGGAAGTAGTGAAG

AATCACATGCAAGCACTGGCACCCAACCCCATTTTCGATGACGACTTCTGGATACGGCACTATTAG
CCTGTACCACAGCAGCCACAAGAAGTTGTTTGCAGCGAACGCAGTGAAAAGGTTCATGTGAAGTGT
GTTCCTGGGGCATCGTAACGATTTGAAATATGACAGTATGAAGAGAGATAGTAAGAGAGCTACTAA
CGATATCGAGTACCCAGTTTGATAAATATTATTGACAGTCTGCCTCAATGTGAGATCTTCAATGTT
GATGCAAGTTGTGTAGTTGGACCATATCATACCACTGTCCGGGTGCCTGAACCAGGTTCCATTTTG
AGTGCATATTTTATGCGCCAGGAGGT

>Nv_B2
GCATTTTCGGAAGGCGAAACGCTTGGTATCGAAACCGGTGACGAAGTTGGGGCAGGGTGCAAGCGC
GATCTCCCCGGAAGGGGTTGTGTTCCAGCAGGACCATCCGTCGAAGGTGGCCGCGCAGTAAGGTCG
TAGTCCAGTTCGATCTGCGACCTTAGTCGGAAACATATCCAGCTGATGTCTCTTCCTGATTTCACA
CTCCCTTTTTAACTTCATGAAGAATTCGAAGTTCGCTTTGGTGGAGGACGAAGAATTGTCGT

Pigment-dirpersing factor receptor

>Nv_B3
ATCGAAAACAACCAAAACGCAGGGAATTGATAATACACCAATTCTTTGCGAAGCATCATATGTCCT
ATTAGAATATGCCAGAACTGCTATGTTTATGTGGATGTTCATTGAGGGTCTGTACCTACACAACGT
GGTCACAGTAAGAGTTTTTCAAGAAACCTTCAGATACAGACTATACAATTGTGTTGGATGGGGTCT
GCCTCTAGTAATGACAACTGCCTGGGCAGCTTCCATAGCCTCCAAAATGAAAACTAAGTGTTGGTG
GGGTTATAATCTTACCCCTTACTTTTGGATACTAGAGGGACCACGTTTTGCAGTAATTTTGCTGAA
TTTTTTGTTCCTATTGAACATTATACGTGTACTCATAGTAAAATTAAGAGAAAGCCACACAAGTGA
AGTAGAACGAGTAAGAAAAGCGGTTCGTGCAGCTGTGGTGCTTCTGCCCTTACTTGGCATCACTAA
TATTGTCAATATGATGGAAG

CRF-like diuretic hormones receptor

>Nv_B4
GAAATATCTCAGAAAATATAAATTGGGCCTGCATATTGTAATCGAGAAAATTGTTTAGAATTTCAT
CCTATTTGTAAAAGGCTATAATTCATAATTTCATCTTAACGCGACGCCGCGTTAGGGAGGCGAAAA
TGAAACAATAGTGTTGAAAACCGAATGATAAGGTTTACCTGTGGCAGTAATCATAACTTGGGTCAT
CGCTAGATCAAATGCAAATGATAATTCCGACCTACAACCGGGAACGAAACAGTGCACTTGGATGAA
CCAGAGTTGGAGCGACTGGATCTACCAAGTTCCTGCAATTTTAGTTCTTGCTATCAACTTACTGTT
TCTAGTTCGCATCATGTGGGTGCTGATCACTAAGCTGAGGTCGGCGAACAACGCAGAGACGGAGCA
ATACAGGAAGGGCTGCAAGGCACTCCTAGTCCTCATCCCCCTGCTAGGCATCACCTACATCCTCTT
CATCGCCGGCCCCCAGTCAGCCGTCTACAGCAACATCAGGGCCCTACTCTTGTCAACACAGGGACT
GTCGGTGGGCCTGCTGTACTGCTTCCTGAACACAGAGGTCCAGAACACCCTGAGGCACCGGTGGCT
GAGGTGGAGGGAGGAGCGCTCATTGGCCACCAGGGCCTATGCCAAGGATATGTCCCCCAACACCAG
GACAGAGAGTATCAGATTGTACAGCAGGCATGAGATCATGCCATACAGGAAACGGGAATCGACTGG
AAGCGAGTCGACAACGATGACCCTCGTGCACTCTTCTAGGCTGTCCAACGGCCCGAGATCTTCCTT
CCTCCAGCCGCCGTCAGAACCTGTCTGAGTTCATCCGCAGTAAAACCTCCGGCTATGAAGAACAAG
TAATTTTCAGTATTTATACTCGTTTGTAATATAATTTAAAACTACTGAGCTTTAGCTCAAAGGTAT
TGTATATTGTTGTAATTAACTGTTGTACCTTGATGTGAAAAAAAAAATGTTTTCATTGTGTATTTT
AAAA

>Nv_B5
ATCTAATATAATATTAATTTTTAAGTGTTGAAATTAACAATAAATTAATATTGATGAATGAAAAAA
AATCCTATCCATAATGACTGATGAAAAAAAAAAATCTTTAATTTTATGGTTTCTTTTGGATTGAGA
TCTTAGATATGCCCTAACCATTTGACACAATATGTATTAGGAAATTAATCCATAAAAAAAAAAGTA
GACCTAACATAATGTTATCTTTACAATTTATGGAAAATATTTTATTTTACAGTAAAATGGAAAAAT
GATTTCAGAAAATAGTACCTATCAATGTAGTTGAAACATTTGACAATTTAAAAAAATAAACAATTA
AACTATGAGATTTCTTCATTGTTTACTTATCTGAGGATGTATATTCAGGAGACAGAAGATTGGTTT
CAAATAGGACATTGATTTCAAACTGGCTCTGATGGAGGCTGCAAGAATGGTGATTTGGGTCCATTG

GACATTTTGGCATTAGCTTGAACTACAGTCATGGTTGTAGTTTCACTACCCGTAGACTCGCGTTTC
CTGTAGGGAACGATCTCATGCTTACTGTATAACCTACAAGTCACACAATCTGATACTCTCAGTCCT
GGTATTGGGCGAGGAATCCTTGGTGTACGTCCTGTGAGGTAATGATCTTTCTTCCCTCCACCTTTG
CCAACGGTGGCGGATAGTATTTTGTACTTCAGTGTTGAGGAAGCAGTACAACAATCCTACTGTCAG
ACCCTGAGTGGACAGAAGCACAGCCCTGAGGAGGTGGTAGACCTCGGTATTAGGGCCAGCAATCAA
GAGTATGTAAGTGATACCCAGAAGAGGTATGAGGACAAGGAGAGCCTTGGAACCCTTTCTGTACTG
CTCGGTCTCTGCGTTGTTGGCTGACCTCAGCTTGGTTATCAGCACCCACATGATCATGCAGAGAAA
GAGCAAGTTGACCACCAGGACGACAAGAACTGGGATGATGTAGATCCAATCTGTCATGCTGGTGTA
CATCATCGTGCACTGGTTCATCGTTTCCGGAACTCCAATGACGGAAGCGTTAGGGTCTTGGACGGT
CGTCCTGGCGATTATCCACACTATTATGACGGGTATAGGAGATCCCCACCCTATAGCTAGGTAAGC
ACGTAGTCCAATATTCTCTCTGGTGAAAGTCTCCACAACCAGCATATATAGGTACAATCCTTCCAC
AAACATCCAGAAAAAGTTGGTCAAAAGGAAATAATGTAGAAGAGTGAATAGAACCAGGCACGATAT
AGAGTCTGTGTGAATTGATACCTGAACAGTTAGCGACAGGATCCACATGAAGTCTGCCAGGACGTA
AGTGAACATCAAGTTTGTGTGAATCGTGTTCCTAAGGCACCGCATCTCT

>Nv_B6

AGTAAATCCTATAAAGAGGTTGCTCTATTGGATTGATTACGGCCAATATCCCAGGATAGTAAAAGC
TTACCTAGATGGCTCTAATTCTTCTCCAATTGTCTCGTCAGGAATTAGTACACCGAGAGATCTTAC
GATTGATATGATGACACATGATGTTTATTGGGTCGATGCCAGATTAGACATGATACAAAAGGTTTC
CTCCAGTGGTGGTAACAGACAGATTATTCGTCGTAACCTTCCGAATCCCATGGGCATCGCTGTTTA
CATGGGATCAGTTTATTGGGTGGACAGAAATCTACAAACAGTTTTCAAAGCTTCAAAACTCCCCGG
TAATACATCGCAGCCGATACCTGTTCGGAAGGGTCTGCACCGTTTAAGAGATATTGCGATTTACGA
CATCAACAACCAACCCCCAGATGATAACAATCCATGCAAAAAACTAGGTAATGGAGGTTGTCGACA
ATTGTGCTTCTCTTACCCTGCTGATTATTCCCTGAATGCATTCAGATATCGATGTGACTGTTCAAC
TGGAATGCTAGTAGATGGTACAAGGTGTGAAAATACCTTAGACTATGTTGTATTCTCTACAAGAAC
TGAAATAAGATCTATTTATCTTGATCCGAAGTCTACAAATGTGCCATTCAAGCCTGTTGGAAATCT
TACAAATGTGGTTGGAGTCGAATTTGATCGTGCCGACAAGAAGCTGTTCTATACCCAGATAAGACC
TTTTGCCAAAATCGCCTCTGTGCCCTCCGATAACCCAGGAGCTCTGAGCACAACTCTCATAACCAG
AGGCATCAATCCCGAAGGTATCGCTTACGATTGGACCCAGAAAAAGATTTACTGGACAGATTCGTC
AAATAATTCTATTTATGCAATGAACTTGGATGGATCCGATCTTGTAATGATCACTAGAGTTGAAAG
GCCAAGAGCAATTGTAGTCGATCCTTGCAATGGAACTCTTTATTATACTGATTGGGGAAGATTCGG
AACATCTGGAAAGATATTCAGGACAACTATGGCTGGCTCTTTGAAACGAGCTATAATTGACCGAGA
TCTCTCTCAGCCTAGTGGCCTTGCCCTCGACTACGAAGACAAGAAGCTGTATTGGACAGATGCAGT
CAGAGAAAAAATTGAAAGAATAAACTTAGACAACGGTCCGCCTAAAAGAGAAGTTCTTATATCTGC
TACGATTTACCCATTCGCCATTACTGTTCTCGACGATTATATCTATTGGACTGATCTTCAGCTGAG
AGGAGTTTACAGAGCTGAAAAGCATACAGGTGCTAATATGGTAGAGCTTGTCAAGCGATTAGATGA
TTCGCCGAGAGACATCCAGGTATTTTCCCCTAAAAGACAGACCTGCAATGTCAATCCATGCCACAT
AAATAATGGAGGTTGTGCCAAGAGCTGCCACCCTGGTCTCCATGGAAAGGCTGAATGCAAGTGTGA
TGATAATACAAAGCTGGTTAATGATGGACGGATGTGTGTTGCGAAAAATCTTACATGCGAAGGAAA
CAAATTTGTCTGTGCAAACGGTAAATGTATTTCAAGAATGTGGGCTTGCGATGGAGATGATGATTG
TGGTGATAACAGTGATGAAGACTTTAAATTCTGTGCTTTCCATTCTTGTAGTCCAAATGAATTCCG
ATGTGCAAACGGACGTTGTATATTCAAATCCTGGAAATGTGATCATGAAAATGATTGTAAGGATGG
ATCAGATGAAATTGATTGCGATTATCCAAAATGTGCTGAAGGAGAGTTCACCTGTTTGAACCAGAG
ATGTATCCCTATGGCACAGGTTTGCAATGGTGTTAATGACTGTAAAGACAATGGTACCTCTGATGA
AACTCACGATAGGTGTCCAAAGAACACAACATGTCCGACTGGCCACCTGAAATGCCAAAACACCAA
CATATGCGTGGAACCTTATTGGCTCTGTGATGGTGATAACGATTGTGGGGACAACAGTGATGAAGA
TTCGTTCCACTGCGCTAAAACGACTTGTCCACAAAATAGCTTCAGGTGTCCTAATCACCGTTGTAT
CCCAGCAACTTGGTATTGCGATGGAGATGACGATTGTGGTGATGGCTCTGATGAACCACCAGAATA
CTGCAAAAGCGAAGGTCGCACTTGCTTCGGTGACTTGTTTACTTGTGATAATGGCAACTGCATTCC

GAGAATATATATTTGTGATGGTGACAATGATTGTTTGGACAATTCAGATGAAGATTCTAGACATCA
GTGTCATGACAGGAAATGTGATGAAGAGACAGAGTTCACTTGCCAAAGCAACAAGGCCTGGGGAAG
GGCTCAATGCATCCCGCGGAAATGGCTGTGCGATGGAGATCCCGATTGTGTTGACGGAGCTGATGA
AAATGCTACTGCTCTCCATTGTCCTCCTCAACCTTCTTGCGAGCCCGACCAGTTCACTTGCGGCAA
CGGAAGGTGTATTAATAAGGGCTGGACTTGTGATCACGATAACGACTGCGGTGATGGCACTGACGA
AGGAAAGGAATGCCATTCTGTTTACAAAACTTGCTCACCACAAGAATTTTCTTGTCAAAACTTTAA
ATGCATACGAATGTCATATAAATGTGATGGAGAAGATGATTGTGGTGATAACTCGGACGAATTTGA
TTGCAAAAAAGAACAAACAACATGCCCAACCGGTCAATTTAAATGTAACAACGGCCAATGTATTGA
CTACCAAAAGGTGTGTAACAAAGTGGCAGATTGTACCGATGAATCTGATGAACCTTTGCATTGCAA
TATTGATGAATGTGCTAAAGTGGAAGTGAACCAATGTGGTCATAAATGTGTTGACACTCTCACTGG
CTACTATTGTGAATGCAATCCTGGTTACAAACTCCTCGATGATGGAAAAGCTTGTGCTGACATTGA
TGAATGCACTGAACAATTGGGTGTCTGTTCTCAATATTGTTCCAACACTCCTGGTTCTTATTATTG
CAAATGTGATGAAACATATTATGACAGAGCTTCTGACGAGCATACATGCAAAAGAAGAGACAGGAA
TGTTCCGTGGATTGTTTTCACAAATAAATATTACATTAGAAACATGTCATTGGATGCGTCATTGTA
CTCTCTGATCCACCAAGATTTGACTAATGTAGTCGCTTTGGATTATGATTACAAAGGAAATACGAT
TTACTTCTGTGATGTTACTGCGAAGACTATATTCAGAACTGAGATTGGTACAAATGAAAAAGAACC
AATTATTCGGCATGACAGCCACGGTTTAGAAGGATTATCTGTTGATTGGGTTGGAAGGAAGATTTA
TTGGTTGGACCGTCATAGTAAACACTTAGATGTAGCTGAATTGGATGGTACTAACAGAAAAACATT
GAAGGCTGGTATCCAGGATCCGAGAGCCATTGCTGTACATCCTGGCATTGGCTATCTATTTTTCAC
TAGTTGGTACTTGCAGGCATTTATTGGAAAGATGGGTATGGATGGATCGAACATGACTCGTATATT
GACTCATGAAGATGGGTTGGCCTGGCCGAATGCTCTCACTATCGATTTCTTTTCGGATCGTATTTA
TTTCGCTGATGCTCATTTAGATTACATAGCTTCTGTCGACTTTGAAGGAAGGCACAAGCATATCGT
TTTGAAAGGAGATAAAGTACCACATATTTTTGCTATAACATTGTTTGAAGATTATATTTATTGGAC
TGATTGGAATCTTAAGGGAATTTTAAGAGCCAACAAGTTCAATGGACATGATTACAAAGTATTAAG
AAACACAACCCATAGGCCTTACGATGTTCATGTTTATCACCCATTAAGACAGCAGGCATATCCTAA
TCCTTGCGGAGGAGATAATGGTGGCTGTTCTCACCTGTGCTTGCTGTCACCTGTGCCCGGAGTGGA
CGCTTCGCCTGATGGATACGGCGATGCTCAGAGCCCGGTAGACTTCAAATGTGCCTGCCCTAATCA
GTTCATACTGGGTTCTGACCAGAAGACCTGCATAGCAAATTGTACCAACGGGCAGCATAGATGCGG
AGGAAACGATGATAGGTGTATTCCCTGGTTCTGGAAATGTGACGGAGAAAAGGATTGTAAAGACGG
TTCTGATGAACCAGCGGAATGTCCAAAACGAACTTGCAGAGCAGGAATGTATCAATGTGATAACGG
CATCTGTACCGCATCAGCAACAATTTGTGATGGAAACAATGATTGCTCGGATTGGTCTGATGAAAA
GTACTGCAATTTGACTTGTCCTGATTTAGAATTTAAATGTCGATCAAATGGAAGATGTATCCTCAA
TGCTTGGGTATGCGATGGAGACCCAGACTGTAAAGATGCTTCAGATGAAGACCCTGCCCACTGTCA
TACTAGAAAATGTGATAATGATACAGAGTTCACTTGTAAAAATGGTAGATGTATTCCTAAGTTATG
GACCTGTGATTTCGACAATGACTGTGGTGATGATTCTGATGAGCCCGCTTATATCTGCAGACAAAA
GAATTGCACTGATGGATGGCAACGTTGTCCTGGTCGAAGCAGTTACCGGTGTATTCCAAAATGGCT
TCTCTGTGACGGAAAAGATGATTGCCGTGACAACAGTGACGAAGATCCTGCCAACTGTCTTAAGTG
TAACACAACTACTGATTTCCAATGTGCTAACAGTCGTTGTATACCTAAGCGTTGGATGTGCGATTT
TGAAAATGACTGCGGAGACAACTCTGATGAAACCGAAGCATTGTGCAAAGGTTCTTATAGGCCTTG
CTCTGAATCTGAATTCAAGTGCGCCAACGGCCGTTGCATACCTCAGCATTACAGGTGCGATCATGA
TGACGACTGTGTTGACGGCTCCGATGAAGTGGGTTGCTCTAACTTCCAATGCAAGAACGGAACGTT
CCAATGTGCCAGTGGCCACTGCATCGCTTCCTATTTCCGTTGTGATGGAGATATTGATTGCCGTGA
TATGAGTGATGAAATGGGATGTCCACCTCTTCCGAACGGGCGATACTGCAGAGAAACCATGTTTGA
ATGTGACAACCATATTTGTATATCTCACCAAGACAAGTGCGACGGAATGGACGACTGTGGTGATAA
ATCAGATGAATCTCCAGAACTTTGTGCTCATGTCCAATCTTGCTTCGGCCGAGGACGTTATCAATG
TGAAAACGGTCGATGTATTACATTCGACGTCATGTGTGATGGTCATGATGACTGTGGGGATGCTTC
TGATGAGAACAATCATTCCTTATGTTATTCACCACCTCGCCGACCCCATTGCACACCTCAAGAGTT
TGCTTGTGACAACGGTAAATGTATTCCTGAAGATAATGTTTGTGACTATTCTGATACGTGCGGTGA
CAATTCAGATGAGATCGGCTGCCATCATGGAAGTTTGTGTCCTGGTGACGGAACAATGGGTGGTTG
TGAACAATCCTGTAGAAACCTGACCAAGCAAGCATTTATCTGTGGTTGCCATCGGGGATATTCCAT
TGATCCAGACAATAGGAAGAAGTGCATAGATGTTGATGAATGTGCAAATGGAGCCCACCAATGTTC
TCATAAGTGTACCAACTTAAACGGCACCTATGCTTGTTCTTGCAGTCCTGGTTTCTCGCTATTGGA
TACTATTAGTGGTGTTTGTAAAGCTGATGATCCTAAGGTTTCTGTCATATTCTCGAATGGAGAGGA

GATTAAATCATATCAAGCACATATGCGACACGAAGTAGATGTAATTCGCAATGAAAAACGCATTGA
AGCTCTCGATTACAATCTCAAAACTGAAATGATATTTTGGGTTGACAGTTACGAAAAGAAGATTAA
AAGAAGTTATATGATAGACGCTTTGGAAGGACGTGTTAAAGTTGGATTCGCTCAAGATCTAACTGT
CAAAGGTAATGCCAAACCAACAAGCATTGCAGTAGATTGGGTCGGTGGAAATCTATATTGGGCCGA
GACAGACCGATCTGGTACTAAGCCTAAAGGGCGCATAGTTGTAACAAAACAAGATGGCCGATACAG
GCGCTCCTTAATAGACAGCAACCTAGAATATCCAACATCAATAGCAGTTGATCCACAATACGGTCT
AATGTTTTGGTCAGATGCAGGAGATGATCCAAAAATAGAAGCTGCTTACCTAGATGGCTCTAGGAG
AAGAGCTTTGGTTACAGAAGGAATACGTCACCCAGCCGGTCTCACCATTGATTATGCAAGCGACAA
TCACCCAATTTATTGGGTCGATACTAAAATGAATTTGATTGAAACTGTTCATCAAGACGGAACCAA
ACGAGAAATTATTCTCAAAGGAGATGCCCTAAAACATCCATTATCCTTGGATGTTTTCGAAAATAA
TCTATACTGGGTCGCCAAAGATTCTGGAGAGCTTATAAAACAGGACAAATTTGGTAGAGGAATACC
AGTATCACTTGCCAAAAATCTAGTTAATCCAACCGATGTTAAAGTTTATCACAAAGCAAAATACAA
TTTGAGTATCCCTAACCCATGCGAGAAAAAGCCATGTGATCATCTTTGCCTGCTACGGCCTCGTGG
AGATTATTTGTGTACTTGTCCTGACAACACGAAAATGATACAATCTGAAACGGAAACAATATGCGA
TTCAGCTACTGAAGAGCCTCTTCCATTACCAAGAGTGTGCCATTGTCAGAACGGTGGTCTATGTCA
GGAAGATGATACAGGAGGCTTGATTTGTTCATGTCCTAACGAATTCACCGGTAGTGCTTGTGAAGT
TTACGTACAAATGGTCCGTACGTCAGGTCCTTCGATTGCACTAATAGTTCCTCTCATAGTACTGCT
GGTCTTGGCATCTGCTGGTGCAATTTACTACGTGGTTAGGAAACGGCCTTTTGGAAAAGGACCAGT
TTTGAGTAGTTTAACGAATTCGCAGAGTGTTTCTTTCAGACAAGGAACAAATGTAGAATTTGGATC
TCCTGCATTCAGTAGTAATGGTCCATCAATACCGGATGCAATAGATGTTGAATATAACCTGTCCGA
TATCAGCGGTAAGAATAGAGACTTCAGTAACCCGATGTATGACGCTCTTGGGAACCTAGAGGCCTC
TCCCGCTGAAACCAACGGGACTGGGGGCCTATATGACGATATGAAGACTGGAAGGGCAGTTATAGA
GCCATCCTCAGCTGTTTTAGCGCCCTCAGTAGTAATGAGTAAGTCTCCTGGCAAGGGACGAGGTCG
ACACCGAGACCTGGATCCTTCCGCAGACACAGGAAAAGATACCCAGAAATTGGTAGAAGAAGACAA
GTCAGACTGTTGACACAAGAAAGCTGCATAAAGAGGTCATTCGCGCCTCGAATGTGCACCATGCGC
TGTGATATATTTACACCGTGCACAACCACAGACAGTCTTAGAGAAGACATATCATTTTTATGAATA
TGAAAACAAAGAAGTCTAAATACAAAAAAAACGTTTAAAATGATTCGAATATTACCTAGTATTGTA
AATACTGGAGCATACAAATGAAGGACTCGAAATGAACCTTGAAAATATGTAAATTTATTATTATAT
TTTTTTTTTTAATTTAGGAAATGTAATTGTCGCAATGATCTGTAAGATTTCATAACAGAAACACAA
TGAATGTGAGCAAATTCCGTATATACATATATATATGTTTCAAATGTATGTATGTATATATATTTT
GTATAGTATCTGGTGCTCTTTTAATTATAAGTTGTACAAAATGAGATGAATGGGGCTCCAAATAAT
TTCAAGGCACTTATCCGTCCAGGCTTGAATATTATTTTTAACTTTCCTTTGTTCCTACTTTGAATA
ACATTCCCTTCGTTGTGTATTATACTTGTTATCTGTTCACTGTGTTATGTTGTTTTCAGTTAAAAC
TTGAAAGTGCTTGTAATTTTTATAGGAATTATATTTTTCTTAGATACAATTTCCTACAGATAAAAA
AAAAATGGAAGAGTGATTTTTCAATAAAGGAGACGATATTCTTTATTCCGAGTCGATATTCCAAAA
AAAAAATAAATAAATGTTATTTTTATATGATGTTTAGGTTCTGGTGTGGAG

>Nv_B7
CTTGTAACAGTACTTCATCACATTTTACTCGACCCAAGATCCTTCATGTGTTGTTAGTGGTACAGG
TTTATATATATATATATATGTATATATAATTTATTTTATTAATAATTATTTCGTATTAAAAGATTA
TAATAGTACAGCTATGAAGAATCTAAAATGATGCTATGTAAATATAATATTTCATACTGATGCTAA
AAAAAAAAGTAGTAATAATTTTTATTATAATCTTTTAATGCATCCATCCAGTTGACCAAAAAGAAA
AAAATATAGTTGAAATATTAAATGAAGATATGTAATTTTTACATATATTTATTAAAAAGGAAGAAA
CTAAGGCAAATATAACATTTGTGTAACCAAAAACTGATAGCTTATATTTAATTATATATAACAATA
ATAACATTATTATACAATGTAGAAGCGTTTTTTGTCACAGCCCTAAACATGGGCAAAAACAGTTAT
AGTGGGGGAAGTAATCCCTTTCGTTCTTCCTCTTCCAGTAGACATTCATACACAGGATTGGAACCT
TGTGTTTGAACTAAACAAAATTCTCTGTTAGCAGAATCTTCTCCTTCATACATAGGATTAGTTATT
TCAACATTCTCTTCCATGCGAGTATGAGCAAACTGTCTACCACGACCTTTAGTCAAAACATACAAG
ACAGCTACTGCACAAGCTGCAACAAGCCCGATAACAGCTAGAACCCAAGGCAACCAACAGAGTAGG
CAAGGACCATTCATTGAGTTGTGATAAAAATCTATGTTGCAATGTGCTCCCTCACATTTGGTGCAA
AGCAGACCAGAGTAACCATTTAAACATTGGCACCCATTTTGTGTACAAATACCATGATTCTCACAC
AAGCACTCTTCACAGTTTTTACCTGTATAGAAAGGTAAACAGATGCAAGAGCCATTATCCTGACAT
TTTCCGTTGTTTTTACATCTGCATTCTGGAATACCACACTTATCACCATGGGGGCACAAGCACGTG

ATGTTCTTAGGCCCAAGGAGACAGAGTGCTCTCCTCTGGCAGTGACAGTGCACAGAAATATGTGGC
TGCTTTTGCTCTTGAACAATATGCAGAGCAATTGGGCGGTCAACTGTCTTATCTACTATGACTGTC
TCATTGTTCCACCTATCAATGCGAACAATTTTATTCTTGATATACATTGATATATATACAGATATTCT
TCAAATATATCCAATTTGTGAGGTTTCCAGTTGTGCTCCCCTTTATAGTGTACAATTCGCTCTGTA
CCTTCAATGAGTATGGATACTATGGCATTAGATCTAGGGTCAGCCCAGTAAAGTCGAGATTTTACC
AAGTCTAAGGCTATACTTGATGAAGTATCGTTGACTAACAGTGAGCGGTCAGTACCATCCATACCA
GCCTTGTAAATACCACCAACAGAATTCCAGAACATCAATCCCCTGTTTGGATGCAAACAAATATCC
CTTGGATCTTTCACTTTAGTTACTATCTTTACTAAGTCACTAATCTGATTATTGATAACAGCAATA
CTACCATCCTCACCATAGCCTAGGGTCATATATAGATTCTTGCCAGCCCAATCCCAAGCAAGTCCA
TGACTGTCCTCATCATTCTCCAAAATTTTCTGACATCTACCAACAGCTGGCTTGTTATTCATAGCA
CACCTAACTATAGCACGACCATAGGAGATATAAACCACAGAAGCAGCCACATCATGTGTTACAGAG
GTAGGTTTGTCTTCAGTGGAGTTAGGATTTATGTCAGAAAGCCAATTACTCTTATACGGGTCATAA
AACCTGATGCCTGAGTCTCCAGCATACAAAAGTCTAGCTGCAGCACCATTAGCTCTGCAAGAGTTA
GTAGAGTTATTAATCTTTGTATATCCTTCAGCACATTCACATTCCACTTTATGAGCACCCCGCACA
GCACAGATCTGAGGACAGTTGTAACAAGGTTTGTTTGGGCAATTTTCTTCATCAGATCCATCTGGG
CAGTCATACCAGCTATCGCATAGCAATGAATGTGAAATACAATGACCAGAAGAACAACGGTGGCCA
TCACAACTTAATAATCCAGCACATGCTTCAGACGACTCATCAGAATTATCACCACAAGTATTTACT
CCATCACAAACATTCACTTTTGGTATACATTTGTCATTCAAGCATCTAAATCTGCTAGGTGGGCAG
ACAAATGATGCGCAATCTTCCTCATCCTGTCCATCTGGACAGTCTTCATGAGCATCACAACGCCAT
GCACTAGGGAGGCAGTGACCAGAACAGAAAATATCACCATTTGAACAAGTCTTATTACAGCCTTCC
TCATCTGACCCATCCAGGCAGTCAACATCGCCATCGCAACGCCACTTCATGAAAATGCAGGTCTTA
GGTTTTCTACATTCAAATTCTGATCCTGGAGAGCACTTTGTAGGGCAATTCTGCTCATCTGATCCA
TCATCACAATGGTGTTCACCATCACACTGATGTAAGCCACTAATACATTTTTTATCTTTACACATG
AATTCTGATTCACTGCAATTTCTTGGATTACAGTTGATCTCATCTGAATTATCATGACAGTCATCT
TCATAATCACACTTCCATCTACCAGGAATACATTTGTTGTTTTGACACTTGAAATATGTTGGTTCA
CAGGTATATGCTGTATCAGCACATGACTTAGGCTCATCAAACCCATCTGGACAGTCTGCTACTCCA
TCACACACCCATGATTGTGGAATACATCGTCCTGAACTGCATGCCAAAAGATCAGTAGGACAGGTT
CGGTTAGCACACATTATTTGTTTTTCATCAGAACCATCAGGGCAGTCCTCAGATCCATCACAAATC
CATACTTGGGGGATACATTTTCTACCACCACATTGAAAATGATCTGGAGAGCAAGTAACCTCCTGA
CAATTATCTTCATCTTGACCCAAGGGGCAGTCCAATCTTCCATCACATTGTTTTTGTCGAAGAATA
CATTTGTCCTTAACTGTATTATTACCCACACATTTAAATTGGGAATTAAGACAGGGATACTTTTCA
CAATTCTTCTCATCAGAATTGTCTCCACAATCGTCTTCACCATTGCAAACCTGTCCTGGGTGGATA
CATTTTCCACTGTTGCACATATGCTGGCCAGGGACACATTTAAATGTTGGACAATCATCGGGTTCA
TCAGAAAAATCTCCACAATCATTTTGAGTATCACATTTCCACCAGAAGGGTATGCATCTATAGGTA
GACTTGCACTCAAACTGAGCATTTGTACAGTTTGAAACACACTTATGCTGGTCTCCACCAATATAA
TAATTCTCAGGACAATCACAAGTCCTACCACCACCTGGTTTAAGTAGACAAAGGGTGTCGCAACCA
CCTCCATGAGCACATGGATTTTTTTTCAGTTTTACCTGCCTTAAAGGATGGTAAATGCGGAGATCC
ATTGGCCTGTGTATCATATTTACTAATCTCTTGCATCCAGTCCCATTTCCAGGAAATCCATACTTA
TCACATTTTTCAATTGACTTGGTTTCCCAATCAGTCCAGTATATTGAATCTTCAAAAACTGCAATA
GCGAACACATGATGCAAACTCAAGGATGGTATTTTACTCCTAGATAAAATAATCCTCCTATTTTTG
CCCTCTAAATCTGCACAAGCTATGTAATCCTCCCTGGCATCAGCCCAGAAGATTAATTGATCATCA
TAGTCAATGGTAAGTGCATTTGGCCAGCCAAGCCCCTCTTGTATAATATATCTCTGTTGAGAGCCA
TCCATCCCAGCTTTTCCAATATGTGGATTTTCTGACCAGTCCGACCAGAATAAGTATCCCTCTTGT
GGCAACAGGGCAATAGCTCTAGGTTCTGAAAGACCCGTTTTGATCAATATTTTTCGATAACTTCCA
TCAAGTTTTGAGACTTCTATTGTATCTGATTCTTTATCACACCAATAAAGGTTTCTACCAACCCAA
TCCACAGCTAATCCATCAGGGTTGTCCAAGTTTGAAGAATGAAGAGTTTGTATCTGTGTATCGCTG
GGACAAAGTCTATTAATGGAAGCTCGTAGAGCAGTCACGTCTGACCAATAAAAACAACCGTCCAAC
CAATCATAATCAAGAGCAACTGCATTGGTCAGATTACTAACAAGAATAGGTGTTGTAGAATGGCCA
GTTGAATCCACTCTTCGAATATAGTACTTATTGGCAAATATTAGCTGAACTTTATCAGTTGAATTT
AATTTACAAGTCCGCTTATCAGGTTTTATTTCATAGCCATTATAACAGCTACATTCATATGATCCG
ATTCTATTAATGCATTTCTGTGAACAAGGACGTGAAGTATTAACATCATTACATTCATCAATATCT
CCACATCTTGTACCATCATGGAGATAAAATCCAGGTAGACAAGAGCATTTAAAGCCAACAGGGAGA
TCAGTGCAAATATGTTGGCATGGATTTGTAGCACTGTCACATTCATTAATATTACATAGCTCCTCA

TCAGAAAAAATCAGAACAATCATTAATACCATCACAGGTCAGATCCATGCTTATACAAATATGGTTT
TGACATCTAAATAATCCATTTTCTTCACAAGAATAGTCCTTGCATTCCTTATCGAAGATAGCTTCA
TGGGATTCATTGATACATTTGTTTTTTCCACTGCATAATTCAACTTTTGATATGCATTGACCATCA
GCGCAATGAATTTTATTCTCTGAACAAGGTTCAGCATTTGAAGAACATGTTTTAGGTTCATCAGAA
TTGTCACCACAATCATCATCTCCATCACAATAATATTCCTTCTCAATACATTTGTGATTAGCACAC
TTTATCTGAGTACTAGAACATGAGAATCCAGTTTTGTCAAGGCAAGTTACAATAGATTCATCAGCA
CCATCTGGGCAATCATTTTCTCCATCACATAATAGTCTTTTGGATATACACAAAGATCCACAAGGA
AAACCAGTACAGACTGATCCACAATCCAGTTCATCAGATCTATCAGTGCAATCCAGTACACCATTG
CATCTATTAGTTAAAGGAATACATTCTACAGATGAATTGCATTTAAACTGTTCTGGTCCACATACT
CGAGTACAATTTTTTTCATCAGTGCCATCTTGACAGTCATCTTCACCATCACAAACCCAGGATTGT
TTAATGCATTGTCCACTCGCACACATAAATCTATCAGATGAACAAACAGGACCTACTGTAGTCTGG
TGATGGGTGCAGTTGTGTTCATCAGAATTATCAAGACAGTCATTCTGGCCATCACATATCCAACTA
GGCAAGATACAAGCAGATGTTGTATTGCAAGGAATAAAGGGGCCACCAGCACCATAGAATTGGCTA
CATTCTTTTATAGGACATCCCATTTCATCTGAGGCATCTTTACAATCTTGATCCCGATCACACCTA
AACATCATTCTGATGCATTCTCCTGACTTGCATTTGAATCTGGAATCAGGGCATGGACAATCTTTT
TCATCCTCTCCAGAAGAACAATCTTCAACTCCATCACATATTTTTGACCTATCAATGCAATGACCA
CTGTAACAGCGCATCTCTTTTTCTAAACAGTTACGAGTTACACAATAGCTAATATTTTCATCTGAA
CCATCAGGACAATGTCGGATACCATCACAAGTGAGTACATGAGGAATGCAAGACCCTTCTGAACAA
GTAAATTGTTTAGGAGAACAATCCATTGTGATGTTGATGCAGCGTTTGCCATCCACCAGGATTCTT
CCTGGATGACATGAACACTGCTTGACACCTCTTGAATCAAGATGGCAAATATCTTCACATTCACCA
TTCAAAACTCTGCATGGATTATAAAAACAATCATATGAAGTGTTTGCAACCGCAATGATACCCATA
GGTCGAGGGACATCTCTCCGCAACCAAACCACTTCTTCTCCAGTGTACTTATTTGCTCTCATAACC
GAATGTTGAATCCAATCAGTCCAAAATATGAATTCTCCATAAACAGCCATGTCAAAAGGATGCTGT
GGTGTTGCTTTAGAAAGTACTATTCTATTAGTACCGTCATATTCAGCCCGCTCTATTTTGTCCAAT
CGTGCATCACCCCAATACAATTTTTGAGCACCATGATCTAATGTGATCGCGTTAGGCATTCTTATA
TCAGTTGTTATGATAGATTCAAGACCATAACCACTTGTAAAAGATCGTTGGATGCTAGGGTTAGCA
GAATTCCAGTTGGTCCAGTACAATCGTGAATCACAAGGATCAACTGCGATTCCTCTAGGTTTATCT
GATGATGTCAATTTTACAATCGTCAGAGGACCAGCTGATATGTTGCTAAGATCGGCTTTGTAAATT
GTTGCAGCATTGTTGCATGTCCAATAAAGAGCGTTTTCTACAGGGCCATAAGTTATTCCTTCAACA
CTACCTTGTCTCTCAATGATGACTCTATGGTTAGTTCCATTGAAGTGAACAGCATTGATTGATCCC
CTTTGTATATCAGAATAAAATAAAGTTTTTCTTTCATAGTCATAAGCAAGACCAATAGCATTACGC
ATAAGATTTGTATCTTGAATGGTAGGGAATGGCGCATTAGCATTATTTGCATCTGTTATATGAATA
GATTCAATCTTGATAACTTTTGAATACATAAGAAAAATGTCATAATCCAAGCACTTAGTTCTATCC
TCAGAAACATAGCCATGAGGACAAACACAAATAGGTGAGGAACCATTAAAAAGGCATAATGCAGGA
CAAGTATGCTTAGCACATGGGTTGGTACCCTTTTGACGGGACATAGAAAACACTGTCATGTCTTTA
AGAGAGTCCCCTAAACCTGATGCTAATATAGAAGTCATGTTTCTATTGAAGTTGTATTTCTTGATG
CTCCCCTTTTCTTCTACCAAGTCAATCCAGTAGATAGAACTATTGAAGTATGTGACTGCAAATGGG
GATCTAAGACCATCAGAAAGTATCATTTCTAAGGTAGTACCATCATAGTTGATTCTAGATATGGTT
CCGCTGCCACTATTACACCAGTAAACTTTCCTTGAATCATAATCGAGAGTAATATCATTCACAACA
CTTCCTTCTGTTAGGTTGAAGATATGGTGAATATTGGAACCATCCAACCCAGAGGCTGCAATCCAA
GGTGGTTTGGCATTGTCAGACCAAAATAGTAACCCTGCCACTGGATCAACAGCTAAAGAATGAACA
GTCTCAAGTCCTGAATGGACAACAACATATCGATGACTTCCATTCAACTTCGCTACTTCTATCACA
TTGAGCTTAGGATCAGCCCAGTATATGTTGCCTGATACCCAGTCAACTGCCAATCCTGTGAGCCAG
TCAACAGGAAGGGAGTCAACAGGTTCATGATGACCAATTACAACTTCACGACCGGTACCATCTCGA
TGGACTCTTGTGATTGTACCATGGTCACCATCTGCCCAGTAGATATAATCATTAGCTGCATCATAG
TCAATGCTGTCAGCCATCGATACTCTTGAAATAGGCCCAAGCACTTGATCACCAGTATCATTTTCT
GCTAATGATAGCCCACGCATTTCCCAGTTGATAGAATAAATAATCAGTTCATTTATACCATGACAT
TTGGTAGAATCTTCGGAATCCTTAGTGTATCCAGCAGCACAAGCACAAACCCTTTCAGAGGTAGAT
ATAGGAAAACATAAGTGAGAACAGTTACCCCTATTTATTGCACAAGCATTTGTTCCATTTTGAGAA
GAAGGATTATATATCTTTAATGACATCACTATGCCAGTATTATTCCTTATAAGAGTCGAGTTAGAA
CCTGACGACTTATCAATGCTATATATTGCCAAAGTATCCTGGGCAGCATAATATAGAAGATCATTG
AATAATGTTGCACATAGTGGAGCAGCGTCTTCTCTTAGTGGTAGTTTGGTCAGCTTCCTCAAATGC
ATGTCATAAAACTGTATTGTCTTGCTTTTGACATTCACCCAATAAAGTCTGTTCTTATCAGTATCG

ACAGTGAGGCTTGTGGTATCTATTAAGTCATGGTTGTGATCTGAAGACAGAAATGCTTGAACATTG
TGACCAGACATATCACTTGATTTGAGATAAGTATTTGTATTTTCCTTCTGGGCCCAATAAAGTTTT
CCACGTAAAGGATCTACAGCAAGTGATCTTATTTGGTAATCCTCCCAAATACTAGCACTACTATGA
TCTTCTGTTGTATATACAGTAGTGATAAATTCTCCATTGAGGGTACAAGCAAGAATTTTGTTTCTA
TTTGTACCAACAGAGCTTAGAAACATATTCTCTGATATCCAATCAATTGCAAAACCAGTAGGATGA
TCAATTCCTGTATCTATAATGCTCTCAGGAGTGCCACCACTAAGATTAGTGCGTCGAATGACATTA
GCCTCACTATCTGCCCAATAAATAGATCTGTTCTTAGCTAAAAAATCCAATTGAACAGGGGCCTGC
ACCTGAGGTATACTTATAGTAGGTATAGTGTGATAATAAGGTTCAGACAAGTCTACTCCACGAATT
TCATTGGCACGAGAGAATAACATCACAAGATTATTTTCAACACAGGTCTTTTTGTCGTCAGCAAGT
TTCATTACGTGAGGGCAATCACACTTGTAGATTGAATTGAAATCCAACAGACATAGGTGAGAACAA
TTACCATTATTGATACCACAAAGGTTTGCCACTCCCCTAGGCTGCCTGCTTGGGTGCAGTATTTTG
ATATCAAATGGCTGAGAAAGGGTCCTCTGCAGAACTGTAACTTGTGATCCGGTCCACTTATTAGCT
CTAATTACAGAACTGGACCTCCAATCAGTCCAGAAGACATAGTTTTCAAATAGAGTGATAGCAAAG
GGATGCGATGTCATTTCATGTCCTTTCAACACCTCATGGTGATCTGTGCCAGCATAAGTAGTAGTA
TGTATTGAGTCAGATCGGGCATCAATCCAGTAGATTCGTTCCAGGACATAATCCAACGTCAGGCCA
TTTGGCCAAGCACCATTGCCAATATGATCAATATAAACCACAACTTTTCTCCCATATCCAGCCATT
G

>Nv_B8 (orphan)
CACTACTGCAAACACCCGTATTGCCTTATCGGCAGGGTAGGGTGTAAATAAAATAACTCAAATAAA
CTTATATTAAAAAGAAAAATATATTGATTTATATTTATTCAACATTTATTGTATTGCGCGTATAAT
TATAGATATATGAAACATATTGACTGATTGTATGTAATATAAATGTGCTGCATGGTAGTAATAGTT
TTTTTACAGCACACGTGCCGCTTTGCTTGTTTTGCTTATGCATGATGGTAGCGCATTGAGTAGCCG
AACGAGCCATCGCTTTCTTGGTTATTATAGCGAGATTGTATCGCAGGTGGTTGAGGAGATAAAGTT
TAACCAGCTGGTGCTGTACGAGACTTGCATCGAGAAGTGGTCGAGCCAGCGGCTCAAGGTGGGCTA
CGCGGCCTGCGTCCTCATGGTCCAGGCCGTGATCCCGGCGCTGGTGGTCGGCTTGGTCCACGCCCA
GATCGCCGCCTACCTCAACGCCCACGCCAGGACCCAGAGGGACTCCAGGAGGGCTCAGCGCGAGCT
CCAGAGGAACAGGAGGACAACCCTTTTACTGTCTGGTGTGGCGGTGCTGTTCGCCGTGAGCTGGCT
GCCGCTCTCCGTGTTCTCGCTGCTGGCGGACCTGTGCCTGGTCTCTGCGGACTCGCTCTACGTGAC
GCTGGCGGCCTGCCACGTGATGGCGATGACCTCGGCTGTCTCCAACCCGGTGGTCTACGGCTGGCT
CAACTCCAACTTTCGGAGGGAGCTGGTGCAGGTGCTGCCGCGGTGGTGCAGGCGGCCGGCCGTCGA
GAGCAGCCAGGAACCCAGCCCCACCCTCCTCCTCTGCCAGAACGGCCAGAAGCCGCAGCACAACAA
CCCCGCCACCACCTACACCGCCCTTTGAGCCCTCGTCAGCTCGTCAAACAAGGGTGTGAAGTTTCC
ATCAAGAATTGGGCTAAGCCAACTTTGAATTTGTAAAATTAAGAGCTTCTTCCTTAAATGTTCTGT
CTAATTATTGTAGGTCATAAGAGAACTGAAAAGTGAATTTGATTGTTGAATTTAATATATTTAAGG
CATGTGAAATGTTGTGATACGAATCAAGTGGGTATATATTATAAATGTTAATTGTTAGATAGTGTC
TACATTTGATGTTCTGTGTTGTAGCCAGGTTCTTCTTTTATAAATTGGTTGCGTTCGAAGTTTGAA
TTACAAAAAAAAAAAAAAAAATCAACCTAATTTGTAAATAGGATTTAGAAAAGTGCCGGCAAACAT
GGAACACGTTAATTTTCTTTCTTATTTGGAAACAACACTAGTTAAAATTGTAAACTTTTAAAAACG
ATGATTTTCTTAAATTAGTTAAATATTACTATAATTTTATGGCAGTTATCATTCGGTTTATACTTT
TTATGCATTAGTGAACATACTTTACCATCATATTTTCCTTATCATTTATTTATGAATAAATGCGCT
TATAAGTTAGTTACATAAACTAAAACCACTGCTCAATGTTAACAAAGTTACGCTTTGAATTCGATT
AATATTTTCTTACAAAATTTTAAATATATTTTACATAAAATAAAATATTTATTTAAATAAACAAAC
CGTATATCATTTTTTTCTTGTTTCTCCTACCCCATTTTTTCCTTGGAAAAGTTCAAGAATTCATAAC
AAGTTGATACGTAGAAATTCTATCGTTCAAAAGGAAGCAATAAAACCGAACTGCATCACTACAAAT
TCTTATAGTAGAATGTTGTTTATATTTAAGTTTTTTCTAAAAAATAATTTCAACAACGTCATGCTA
GTGATCGATTTTTAAAATGCATGCTATCGCATTCATGCTAACCAGTATTAAAACTTACTACTTTTC
AATATGTAACTTAGCAGCATTGAATGAAAATAATGTTTACTGCTGAATACTTTGTTCTTCTTTTTT
CTCAATATATTTAATTCATATATTTAAAACATCGATACAATATTAAGCAGATAAATATTTCAGTGT
TAAAATCAACTATGTAATATCTGCAATAATGCTTTAATACATTTTATTTATTTATGCACACCCTCT
AAAGTAATGACCATTTATATTGAAAAAACAACAACAAATAAAATGGTTGTTGTTTTGTTTCCCAAA
TTAGAACTGAATACCAAAGAATTAAAATTTAGATGTAATATAGTAAAATATTTTAAATGAAGAGAA
CTTCTGATTAAGCACTTAAATGGTTCGCTAGTCAGTATTTGGTTTTTGTTGTGCCCTACCTCGCCT

TCGGCTATGCTGGGGTTTTCCCGTCCACGAGGTGAGGGAGGGGAATTCAAAAGACCCCCAAGACAC
CGGGACGCGAACTCGTAATTTTATGGTCTACAACTTTCGATATCATGTTGCCAAGCAACGCGCTAT
GCTGTGCACACACGTTAGCCCGTTCGGCCACCCCGCTCGTATTGTGTTATTTTTTAATTGTTGCTA
CGGTTACAGCTTAGCAGTATCATTATTAATTTTTAAACATTTATTATGAATAAATACCGATTCCCA
ACGACAATATGCTTTAGATCCCAAACGTCAGAATGCTTCTTCTTGAAATGATAGTACCCGACATTT
TTAACAGTAAAAACAGAATTAACGGTTTTAACCATGTCTAAAAGAATAAACTTATTAGGTGCGATA
TACATACATGTATATATAGAGATATGCAATATGATCATCGCAGATTTTAATATTGGTAAATTCTGC
GTTCCCCTAGTGGGAGTAAAAAGATAGGATACATTGAATTTTATATATCATATTACGCCTTTCTAT
TTTAAAAAATCTAATTTATACAATATTATGGATTTTGAAAGGTAATCTAGAACTATAACAATCAAT
TCATATTTAAATATTTCGAAAGCTAAATCTCCAAGCAAAACGTTTTAAGTAATAAATTAATTCCCA
ACTACTTTTATTATTTAACTTTTGGATGATAGAATAATACTGATGATAGGAGGAAGAATTATTTCA
ACAACAGCTTGATCTTTAAGATAAAAGAAGAACTGTTTGGCATAATATATATTAATTAAATTTTAG
AAAACTTTTCTATTGTTTGTATAGAGATTGTTGTTAAATATTAATTAGTATTATATAATTGTAAAT
ATAGTATATGTATATAAGATAAATGTGCTAAGATAAATATATATACCCCTAACCACACATATATAT
ATATAGATGAGATATAAGGGTTGCTGTACATAGACAACCGGGGTCGGAGGAAGGCGGGGGCTGAGC
CAGGCCTGAGCTCGGACGCCCGAGAGCAGGTCTCCCCGCTGTCCCCCTTCCCCTAAACTCCCTACA
CTCTATATGCACTATACACTATACACTGTACATATCTCTACTGTTGACTTAGTATCTACTAGTTTA
TAGTAATATTTATCTATATAGCCCTTTAACCATATCTTGTTAATCTTATTATTGTTTGTGTTGGAA
TAAATGTATTTTGAAAATGTAAAAAAA

>NV_B9

GTTTTACTACAACTCCTTTCTGTATCCCCTGGCTGCTGATAAAAGCAGTGGACATGATGTAAACAA
TTTAACTATAGAAGTTTCCTGTTCTGGAAATGTCGATATGAATTCCTCATATATGAATCCTGGAAG
AGCAGAGAAGAAAAAAAAAGGACTTAAAACGACAAGATCAATGGAGTCATTATGAAATTATGCTTGG
AAGCTTTATGAAGCAAGCGCAATTTGTCAGTCAAGTAAACCAAGTAAAGGGTTGGATGAAATTATT
TTTTCTGTGTGTTCTTGTGTGCTACGTATCTGCGATCCAGTTAGAGCCATGTGAATGTATCTCGAT
TCAAAGTTTAGGATTTAAATGTCATTGTGAAGGAAATATTTCAAGCATACAAGTAAAATTGAAGCC
TGGAGATCTTTATTCTTTAAGGATTACTCATACTTCGTTGGTTGAGTTCTCTGGATTTGTTAAGCT
AGGAGCCAAAGGACCTATGGATATAATGGATTTGGAAAGCAACAAAATTGAGCGCATTAGGACCAA
CAGTGCTAATGTACGAACAGAACAACTTTTCCTGGACTACAATAAAATAAGATGTATTGATGGATG
GGCATTCAATGGCTCTGAAATCGCGAGATTAAGTTTGAGAGGAAATATCGAGCTGTCTGAGTTATC
GGTCGATGCATTTAATGGCATTCACAGTTTGAGAGACTTAGACTTGTCAGATACATCTATAACTTT
CTTACCTCCAATAGGATTAGAGAAACTTGAAATATTGAGGCTTACAAATACCTTTACATTAAAGGT
CATCCCATCGATTTATGATTTAAGTAAATATTTGCAACTAAAAAAGGAACTTTGTACGCCCGAAAC
CAAGCGATCAAAAAGGAACATTTACGGTGGCTTCGGAGCAATCATGGATACGACTTCTCAGGATTC
CAAGAACCAATATTCTGATTTTTCACCGAATTATAATGAAACGATTTTAGATGAAACGTTTCATCA
AATGCATACAGATATTCCCAGGGATAAAATTGAAGCACTGTGTGGCAACTTAATAGGCAGATATGT
GTCTTGCTCTCCTAAACCTGATGCTTTGAATCCTTGTGAAGATATGATGAGCTGGTTATGGTTGAG
GGTTTCAATTTGGTTCGTGATATCAGCAGGAATTGTTGGTAATGTAGCTGTCCTTCTTGTTTTATG
CCTCTCGAAAACTGAGAAATCTGTACCAAGGTTCTTGATGTGTAACCTCGCATCCGCGGACCTGAT
AATGGCAACATATCTTTTGATGTTAGCGATAATGGACATTATTTCGTCTGAAACATATTTTAATTA
TGCTTACGATTGGCAAAGAGGTTATGGCTGTAGGATGGCAGGATTTCTGACAGTATTTGCTAGTCA
GCTATCTATTTTTACGTTGTCACTATTGACGATAGAACGTTGGTTCGCAATCAGGCACGCGCTGTA
CCTAAATATGCTTGATATACAAATCACATCACATATTATGATCGGTGGATGGGTTTATTCAATTAT
AATGGCCTTATTACCGTTGATTGGTGTTAGTAGCTATTCAACGACAAGTTTATGTCTGCCAATGGA
TTCAAACGACTACATCTCTCAAGTATATATAATTACAATGATTGTTGTAGCAGGATGTGCCTTTCT
TCTAATGTGCATTTGCTATACTCAAATTTATCTATCTTTGAGTTATGAGACAAGACATTCAATAAG
TGAAGGAGCTATAGTTCGAAAAATGACAATTTTAGTGGGAACAAATTTTTTATGTTGGGCACCAGT
CGCTTTTTTTTTCTTTTACAGCGTTAGCTGGGTATCCATTAATCACTATATCACAGTCAAAAATACT
GCTCGTATTTATATATCCTATCAATTCTTGTGCTAACCCTTATTTGTACGCCATCTTGACAAAACA
ATACAGGAAAGACTGTTTATCGATACTCTCAAGATACTTCTTCTATAAACGATCTCCTAACAATAA
CATGGGCTATTCCAGACCAGTTTCAAACGAAGTACCTGGACAACAGACGACAGCAGAAACCTTACT

TTAAGTTGTAATTAAGCACTGATTTTTATTTTGTAAACTGAGTAATAATATGTTTCTAAATAATAA
AAGTGTTACAAAAAAGAATTACTTTAACAACAAAAAA

>Nv_B10 (orphan)
ATTGGAAGGAAAGAGCGAGCGATAGGGATGATTGGCATGCTATAATCCAGAAAGCCAAGGCCCACC
CCGGGCTATAGAGCTGTACGGTTCACTCATTCCTGATTACAAATCTTGCACTTGGAGATCTTTTGA
TGGGATCGTATTTGTTACTTATTGCTGTAGTCGACTGGCAATATAGAGGTGTTTATTCAATTTACG
ATACCAGCTGGAGAAGCAGTAAACTTTGCTCACTCGCCGGTTTCATATCAACATTTTCAAGTGAAT
TATCAGTATTTACATTGACAGTTATTACGTTGGATCGTTTCTTGGTTATTATATTTCCATTTCGTG
TTCGACGCTTGGAAATGAATAGAACAAAGCAACTTATGGCTTTTGGATGGATAATAGCTATAGCTA
TTTCAGCAGTTCCTCTTCTTCAAATTGATTATTTTAAGTAAATATTAAAATATTCCATAGCCATTA
CTATTACTTTTTATTGTAGATATGTATTTGTCCTTACTTCATTAACTCAAACAAAAACCTTTTTTT
TTTTATCAATGAACTTCAAATATTGAATAAGTCAAAT
